# Reviews in Educational Psychology (Frontiers in Psychology 2010–2024): typology, topics, regional comparative and methodology toward digital and AI approaches

**DOI:** 10.3389/fpsyg.2025.1588242

**Published:** 2025-04-30

**Authors:** Alberto Díaz-Burgos, Jesús N. García-Sánchez, María-Lourdes Álvarez-Fernández, Sónia Brito-Costa, Douglas F. Kauffman, Ting-Chia Hsu, Jesús de la Fuente

**Affiliations:** ^1^Department of Psychology, Sociology and Philosophy, Universidad de León, León, Spain; ^2^Education School, Instituto Politécnico de Coimbra, Coimbra, Portugal; ^3^INED - Center for Research and Innovation in Education, Polytechnic Institute of Porto, Porto, Portugal; ^4^School of Clinical Medicine, Medical University of America-Nevis, Devens, MA, United States; ^5^Department of Technology Application and Human Resource Development, National Taiwan Normal University, Taipei City, Taiwan; ^6^Department of Psychology, School of Education and Psychology. University of Navarra, Pamplona, Spain

**Keywords:** psychology and education, systematic review, meta-analysis, sustainable, development, regional comparative

## Abstract

This study presents a systematic review of reviews published in Frontiers in Psychology (2010–2024) to examine methodological and conceptual advances in educational psychology. The objective is to synthesize research trends over 14 years and explore global challenges, such as the digitalization of education and the integration of emerging technologies. Following PRISMA guidelines, a comprehensive search was conducted in Frontiers in Psychology, Web of Science, and Scopus, identifying 392 reviews. The selection process involved duplicate removal, title and abstract screening, and full-text evaluation, applying predefined inclusion and exclusion criteria to ensure methodological rigor. Data extraction and classification were carried out using an Excel-based structured database, analyzing publication years, methodological design, data sources, statistical and qualitative analysis methods, validation approaches, theoretical frameworks, thematic areas, geographical distribution, study limitations, reported results, practical applications and study populations. The methodological analysis highlights the predominance of systematic reviews, the increasing adoption of qualitative and mixed-method approaches, and a growing emphasis on digital tools and artificial intelligence. The study also reveals significant regional disparities in research output, with some regions being notably underrepresented. Beyond identifying trends, this review of reviews illustrates how psychology adapts to contemporary educational challenges through interdisciplinary methodologies and evidence-based strategies. The findings provide valuable insights into the evolving challenges in educational psychology, reinforcing the role of Frontiers in Psychology in driving methodological innovation and scholarly discourse. Furthermore, they contribute to the advancement of inclusive and sustainable educational practices aligned with the Sustainable Development Goals (SDGs). Future research should focus on meta-analyses of emerging trends, longitudinal methodological studies, and strategies to address regional imbalances, fostering a more globally representative perspective.

## Highlights

- This study synthesizes and critically evaluates educational psychology reviews published in *Frontiers in Psychology* (2010-2024).
- By analyzing 392 studies using PRISMA guidelines, the study identifies methodological patterns, validation techniques, and key constructs explored.
- The research compares educational psychology trends across different regions, highlighting methodological and thematic variations.
- The special issue provides an innovative and integrative approach to educational psychology, emphasizing self-regulated learning, inclusive methodologies, and the impact of family and social contexts on academic achievement.
- The structured database developed allows for further meta-analyses, longitudinal trends exploration, and interdisciplinary applications in psychoeducational research.

## 1 Introduction

Psychological reviews are fundamental tools for synthesizing and analyzing accumulated knowledge in specific fields, providing a robust foundation for advancing research (Higgins et al., 2024; Luyten et al., 2024). However, despite the growing number of reviews in psychology, there are still notable gaps regarding their methodological rigor, thematic focus, regional distribution or practical applications in educational contexts. In particular, the extent to which these reviews address contemporary educational challenges, integrate interdisciplinary approaches, and contribute to evidence-based practices remains unclear. This study seeks to bridge these gaps by systematically analyzing review articles published in *Frontiers in Psychology*, identifying predominant trends, methodological advancements, and areas where further research is needed to strengthen their impact on global educational goals.

*Frontiers in Psychology* was selected as the focal journal for this study due to its high volume of published reviews, its interdisciplinary nature, and its impact on advancing psychological research. As one of the largest open-access journals in psychology, it provides a broad and diverse collection of systematic reviews, meta-analyses, and theoretical contributions spanning multiple subfields, including educational psychology. Additionally, *Frontiers in Psychology* is recognized for its commitment to methodological innovation, often publishing studies that explore emerging trends in research design, data analysis, and technological advancements. Compared to other leading psychology journals, its open-access model facilitates greater visibility, accessibility, and international collaboration, making it a valuable resource for examining global trends in review methodologies. Given its strong presence in educational psychology research and its role in shaping methodological discussions, this journal serves as an ideal platform for analyzing the evolution of review practices and their contributions to the field.

While systematic reviews and meta-analyses have been widely used to synthesize empirical research, comparatively fewer studies have focused on how review methodologies themselves evolve over time. A key innovation of this study is that it constitutes a review of reviews, a methodological approach that has been applied in various disciplines but remains relatively underexplored in *Frontiers in Psychology*. Unlike previous systematic reviews or meta-analyses that focus on empirical studies, this study provides a comprehensive synthesis of the methodological and conceptual evolution of review articles published in the journal from its inception to the present. Although *Frontiers in Psychology* has published some reviews of reviews, no prior study has systematically examined how these methodologies have evolved within the journal as a whole. This unique approach offers a deeper understanding of editorial and methodological trends shaping psychological research, particularly in the field of educational psychology. By examining the evolution of review practices, this study contributes to a broader understanding of how systematic reviews and meta-analyses have adapted over time to address emerging challenges in psychoeducational research (Prothero et al., 2018; Topa et al., 2022; Troncoso Skidmore and Thompson, 2010).

Grant and Booth (2009) identify various types of reviews with specific objectives: narrative reviews synthesize literature qualitatively to provide an overview; systematic reviews apply rigorous criteria to identify and evaluate relevant studies; integrative reviews combine qualitative and quantitative methodologies; meta-analyses perform statistical analyses of combined results; critical reviews evaluate literature from a theoretical perspective; conceptual reviews develop new theories based on previous studies; and rapid reviews synthesize information within short timeframes. These typologies ensure the quality and relevance of conclusions by adapting to different objectives (Kreuder et al., 2024; Su and Yang, 2024).

Recent literature highlights systematic reviews and meta-analyses as pivotal in addressing complex questions and generating reproducible evidence. Updated classifications emphasize qualitative reviews (Graham, 2018), PRISMA (Preferred Reporting Items for Systematic Reviews and Meta-Analyses) systematic reviews and added quantitative reviews (Díaz-Prieto and García-Sánchez, 2016), meta-analyses (Botella and Gambara, 2006; Peng et al., 2019), reviews of reviews, meta-analysis reviews, and meta-analyses of meta-analyses (Hempel et al., 2014; Bouskill et al., 2019). This classification has been adopted in the present study to analyze selected articles.

Methodological rigor in academic reviews is often ensured through frameworks such as PRISMA (Page et al., 2021) and SALSA (Search, Appraisal, Synthesis, and Analysis). PRISMA provides clear guidelines for identifying, selecting, and evaluating studies, promoting transparency and reproducibility, while enabling the creation of visual maps illustrating relationships between studies (Miller et al., 2018; Scott et al., 2018). SALSA, on the other hand, focuses on comprehensive literature searches, critical appraisals, synthesis of findings, and pattern analysis, offering flexibility to integrate diverse types of evidence (Bathaei and Štreimikienė, 2023; Mengist et al., 2020). Both methodologies are essential for systematic reviews and meta-analyses, and their combination with tools for ensuring the quality of researches selected for analysis, such as the Research Quality Model (RQM) ensures robust evaluations (Moher et al., 2009).

Complementing these methodologies, frameworks such as PICOS (Population, Intervention, Comparison, Outcomes, Study design) provide a structured approach to formulate clear research questions and establish rigorous inclusion and exclusion criteria. This framework enables researchers to define the target population, intervention under evaluation, comparison group, expected outcomes, and methodological design, ensuring the alignment of selected studies with the research objectives. Additionally, tools like PROSPERO, an international registry for systematic review protocols, enhance transparency by preregistering the objectives and methods of reviews. The MARS (Meta-analysis Reporting Standards) guidelines further standardize the reporting of meta-analyses, promoting consistency and reliability in findings. For qualitative reviews, the SPIDER (Sample, Phenomenon of Interest, Design, Evaluation, Research type) framework facilitates comprehensive searches and analysis in non-experimental studies. These strategies, when integrated, provide a robust and adaptable foundation for validating the methodological rigor and reproducibility of systematic reviews.

These methodological frameworks are not only essential for ensuring transparency and rigor but also pivotal for addressing contemporary challenges in an increasingly digitalized world (Lim and Kumar, 2024; Passas, 2024; Pradana et al., 2023; Rojas-Sánchez et al., 2023). The integration of advanced technologies, including artificial intelligence and specialized software, has transformed the execution of systematic reviews in psychology. Tools such as Comprehensive Meta-Analysis and R enable researchers to analyze large datasets, conduct sophisticated statistical analysis, and visualize trends more effectively (Ciampa et al., 2023; Wei, 2024).

In particular, reviews play a crucial role in synthesizing evidence related to psychoeducational variables. These reviews not only highlight trends in the literature but also facilitate the identification of patterns and the development of evidence-based interventions tailored to evolving educational and psychological challenges (Farias-Gaytan et al., 2023; Utaminingsih et al., 2023). By leveraging these tools, researchers can address limitations in existing studies and propose new frameworks that advance the understanding of psychoeducational processes (Basilotta-Gómez-Pablos et al., 2022; Díaz-Burgos et al., 2023; Gutiérrez-Ángel et al., 2022).

Given the growing role of reviews in shaping psychoeducational research, it is essential to examine not only general trends but also how specific initiatives contribute to methodological advancements in the field. In this regard, special issues play a key role in consolidating innovative perspectives and fostering interdisciplinary dialog. As part of this evolving landscape, a recent special issue in *Frontiers in Psychology* serves as a particularly relevant case, exemplifying innovative approaches to educational psychology and methodological integration.

Aligned with these developments, the present article examines review articles from *Frontiers in Psychology* to explore their contributions to educational psychology. Particular emphasis is placed on a special issue that offers an innovative perspective on the integration of tools within educational and collaborative environments. Titled *“Reviews in Educational Psychology,”* this special issue directly aligns with the core focus of our systematic review, making it particularly relevant to our analysis. By highlighting this issue, we aim to underscore its significant contribution within *Frontiers in Psychology*, as it explicitly reflects the journal’s emphasis on systematic reviews in educational psychology.

The methodology employed in this study synthesizes predominant characteristics, identifies research trends, and critically evaluates how the special issue advances knowledge in this area. By addressing the intersection of psychoeducational variables and learning processes, the special issue provides valuable insights into fostering holistic development (DeCuir-Gunby and Schutz, 2024; Mayer, 2024).

Categorizing review findings provides a visual and systematic representation of the analyzed information. Systematic reviews focus on exhaustive information collection under defined criteria, synthesizing patterns and results with high reproducibility. Meta-analyses, by contrast, integrate quantitative data from multiple studies to conduct robust statistical analyzes. Both typologies have evolved to address specific research questions, contributing to methodological diversity (Agrawal et al., 2024; Arya et al., 2021; Paul and Barari, 2022).

Beyond meta-analyses and systematic reviews, several other methodologies were frequently employed by the authors. Scoping reviews emerged as a prominent approach, particularly for mapping the breadth of existing research and identifying gaps without the stringent quality assessments typical of systematic reviews. This flexibility makes scoping reviews ideal for exploring emerging areas or complex fields.

Literature reviews were also commonly utilized, providing a narrative synthesis of current knowledge. While less structured than systematic approaches, they are invaluable for establishing theoretical contexts or summarizing broad research areas. Similarly, critical reviews gained prominence for their ability to rigorously evaluate existing studies, highlighting methodological limitations, biases, and areas for improvement. Finally, configurative reviews, which integrate qualitative data to generate new interpretations or theoretical insights, and synthetic reviews, which combine findings from multiple studies into a coherent framework, were notable for their contributions to advancing theoretical and practical understanding.

A complementary approach involves analyzing reviews by geographic region to highlight how cultural, economic, and social contexts shape research priorities. Regional differences may reflect local needs, available resources, and academic traditions, offering a more inclusive view of psychology (Kumar et al., 2024; Morris et al., 2025; Susilawati et al., 2025). The analysis of topics covered in reviews further underscores the diversity of psychological research, spanning educational psychology, social psychology, clinical psychology, and quantitative psychology (McPhetres et al., 2021; Möller, 2024).

Finally, these efforts align with the United Nations’ Sustainable Development Goals (SDGs), particularly SDG 4 (Quality Education) and SDG 3 (Good Health and Well-being). By integrating technology with socio-emotional skills, these initiatives foster equitable and inclusive education while respecting psychological and emotional health (Castaño Muñoz et al., 2023; ElSayary, 2023). The special issue analyzed in this study exemplifies how digital competencies can transform educational and psychological landscapes, providing a roadmap for future research and applications.

This context underscores the need to analyze how reviews related to educational psychology are addressed in *Frontiers in Psychology*. Within this framework, the following research questions are proposed: RQ1: What are the main characteristics defining the reviews published in *Frontiers in Psychology* from its inception to 2024 included, considering methodological, thematic, and focus aspects?; RQ2: How do systematic reviews differ from meta-analysis in terms of scope, methodology, and scientific contributions?; RQ3: What emerging trends are observed in the topics addressed and data analysis approaches in the journal’s reviews?; RQ4: What are the most relevant results obtained from the comparative analysis of reviews by geographic regions, and what regional patterns can be identified?; RQ5: How has the use of tools and software in published reviews evolved over time?; and RQ6: What specific contributions and added value does the special issue provide compared to the overall set of articles analyzed in the journal?

The general objective of this study is to conduct a comparative analysis of the reviews published in *Frontiers in Psychology*, identifying predominant characteristics such as typology, constructs, methodology, and software used, and evaluating the specific contributions of the special issue within the current context. To address the research questions, the following Specific Objectives (SO) are established: SO1: determine the predominant characteristics of the reviews published in *Frontiers in Psychology*, considering aspects such as typology, methodological approaches, and investigated constructs; SO2: compare systematic reviews and meta-analyzes in terms of methodologies and scientific contributions to establish key differences between these approaches; SO3: identify emerging trends in topics and data analyzis approaches in the reviews, highlighting their impact on advancing psychological knowledge; SO4: analyze the reviews from a geographical perspective, identifying patterns and regional differences in study approaches and results; SO5: examine the evolution of tools and software usage in published reviews; and SO6: evaluate the specific contributions and added value of the special issue in comparison with the set of published reviews, identifying its relevance and differentiating contributions.

Based on the proposed objectives, the following forecasts (F) are established: F1: the reviews published in *Frontiers in Psychology* are characterized by a predominance of systematic reviews and meta-analysis, with a wide variety of thematic and methodological approaches reflecting trends in psychology; F2: current trends in topics and data analysis reveal a growing interest in interdisciplinary areas, such as ICT and its impact on educational psychology; F3: significant regional differences exist in the approaches and outcomes of published reviews, influenced by geographical, cultural, and socioeconomic contexts; F4: the use of tools and software in reviews has evolved significantly over time; and F5: the special issue provides significant added value compared to the set of reviews, standing out for identifying research gaps and promoting innovative approaches.

## 2 Methods

**Figure 1:**
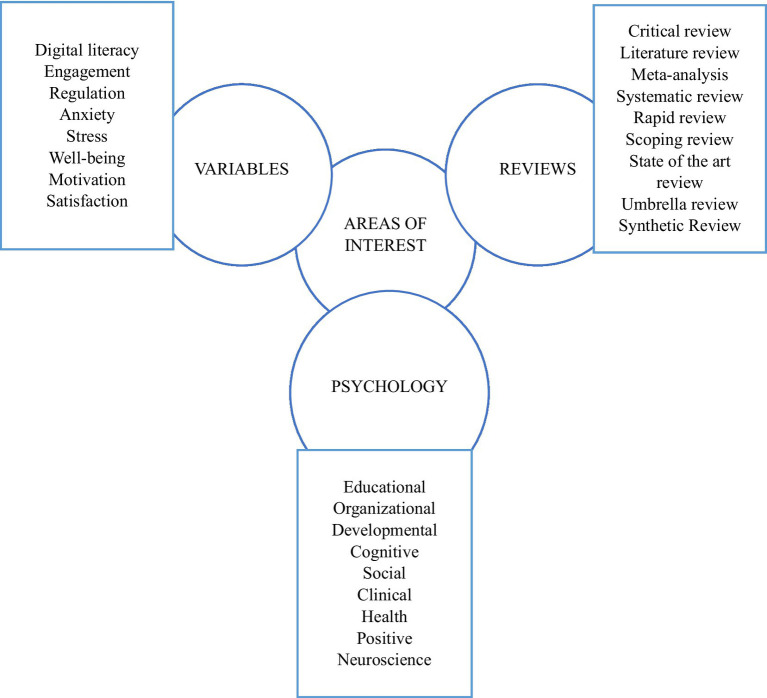
Diagram of key terms. This diagram provides an overview of the main thematic axes and specific keywords used in the bibliographic search process.

This study employed a systematic review approach following the PRISMA guidelines (Page et al., 2021). The process included the following steps: (i) the bibliographic search began with the creation of a diagram highlighting key terms and main thematic axes, utilizing Frontiers website or databases such as Web of Science and Scopus (Figure 1); (ii) inclusion and exclusion criteria were established, incorporating additional parameters such as the selection of studies published in peer-reviewed scientific journals, recognized databases, and citation indexes (Cooper et al., 2009; Miller et al., 2018; Scott et al., 2018); and (iii) once the criteria were defined, they were applied to conduct both qualitative and quantitative analysis.

The inclusion of Web of Science (WOS) and Scopus databases, despite the final selection of articles exclusively from *Frontiers in Psychology*, was part of a deliberate search triangulation strategy designed to ensure comprehensiveness, validity, and methodological rigor. Keywords were combined using the logical operators “AND” and “OR.” In the search engine, results were filtered based on inclusion and exclusion criteria, such as the publication period (2010–2024), open access, English language, and document type (“Review”). The database search was conducted between December 2024 and January 2025, ensuring the inclusion of all relevant publications available up to that period. Specifically, the main keywords employed were “educational psychology” and “review,” intended to identify publications explicitly focused on reviews within the field of educational psychology. An example of a search string used in Web of Science was as follows: TS = “review” AND “educational psychology.” Filters included publication period (2010–2024) and open access. Additionally, a search was conducted on the Frontiers website using the keyword “educational.” This search was specifically filtered to include only documents published in the journal Frontiers in Psychology and further restricted to articles classified as review or systematic review.

The following criteria were implemented through a rigorous process based on the PRISMA model, documenting each stage: identification, removal of duplicates, screening, and final eligibility assessment. This process resulted in the selection of 392 relevant studies, which formed the foundation for the comparative analysis presented in this work.

### 2.1 Inclusion/exclusion criteria

Studies published between 2010 and 2024 were included, covering the entire publication period of the journal *Frontiers in Psychology* up to the date of this article. Reviews related to the field of educational psychology were selected due to their capacity to comprehensively synthesize existing literature. Articles available in full text were required to allow for a detailed assessment and ensure analytical transparency, as incomplete access would limit the ability to critically evaluate methodological and theoretical contributions.

To maintain methodological rigor and enhance the reliability of findings, non-peer-reviewed studies, conference proceedings, and gray literature were excluded, as these sources often lack standardized review processes and methodological transparency, which could compromise the consistency of the analysis. Publications had to be in English to ensure uniformity in data extraction and minimize the risk of misinterpretation due to translation inaccuracies. Duplicate articles were removed to prevent redundant inclusion of the same study. Additionally, only articles published in *Frontiers in Psychology* were considered, ensuring coherence in editorial policies, peer-review standards, and methodological approaches. The removal of duplicates was carried out manually by the authors, ensuring careful evaluation to avoid any redundancy. This manual process was essential to maintain the rigor and quality of the review.

The dataset included all studies retrieved from the *Frontiers* website and both databases (Web of Science and Scopus) after applying the search string. As a result, some studies with a predominant focus on clinical or social psychology were also incorporated when they intersected with educational psychology, particularly in areas related to learning processes, teacher well-being, and cognitive development. Similarly, conceptual analyses were included alongside systematic reviews and meta-analyses, given their role in advancing theoretical discussions in educational psychology. However, studies lacking methodological clarity or explicit review criteria were excluded to preserve the validity and reproducibility of the findings.

Once the inclusion and exclusion criteria were applied, the selected studies were systematically organized to facilitate subsequent analysis, as detailed in the following section.

### 2.2 Procedure

The information gathered from the selected studies was organized into tables created in Excel, classifying the articles based on multiple categories extracted from the analyzed sheets. These categories included data on authors and year of publication, country or region of origin, type of review, number of studies reviewed, age group analyzed, constructs addressed, main topics, theoretical frameworks used, digital tools and software employed, reliability, validation, period reviewed, database used, quality analysis, applied methodologies, types of data analysis conducted, added value of the study, main results, identified limitations, and proposed applications. This approach allowed for a comprehensive systematization of the information, providing a solid foundation for subsequent analysis.

A categorization process was then conducted to divide the studies according to various approaches, such as the investigative topics covered, geographic regions, and type of review performed. This division was specifically designed to facilitate comparisons between articles within each group, enabling a deeper analysis of methodological and thematic differences as well as trends observed in each category. The use of Excel for systematic categorization ensured consistency and reproducibility, allowing future researchers to replicate or adapt the methodology for similar analysis.

Additionally, the articles from the special issue were separated into a specific sheet, allowing for a detailed analysis of their added value and innovation compared to the other studies. This procedure ensured that the specific contributions of the special issue could be evaluated in isolation, highlighting its unique contributions in terms of digital tools, methodologies, and investigative approaches. The entire process of data categorization and separation was designed to facilitate the observation and extraction of relevant conclusions, identifying patterns in the reviewed literature.

To guarantee the application of all the steps and process of this systematic review of reviews published in *Frontiers in Psychology*, the inclusion and classifications were agreed upon by at least three of the authors. This guarantees agreement between coders.

To complement this tabular organization, graphs were generated to provide a visual representation of the key trends and relationships identified in the data. These graphs facilitated comparisons between different approaches, such as the evolution of digital tool usage over time, differences in applied methodologies based on the type of review, and investigative dynamics across geographic regions. By offering a visual perspective, the graphs allowed for a more intuitive interpretation of the results, simplifying the identification of emerging trends and providing a comprehensive framework for discussing and analyzing the conclusions obtained. This integrated methodological approach, combining systematic categorization and graphical representation, ensured a rigorous and detailed analysis of the reviewed articles, optimizing the clarity and depth of the results.

## 3 Results

**Figure 2:**
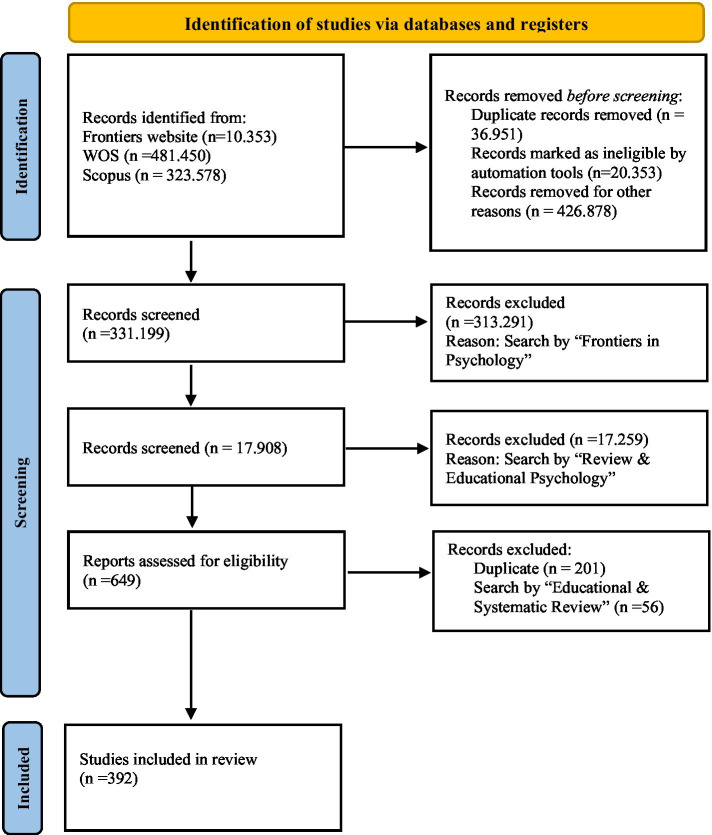
PRISMA flow diagram. This flow diagram outlines the systematic review process, detailing each step from the initial identification of studies to the final inclusion (Moher et al., 2009; Page et al., 2021).

A total of 392 articles published in *Frontiers in Psychology* were reviewed, obtained through a systematic search following the PRISMA model (Figure 2). Each article was analyzed and categorized into tables that organized the information based on various criteria, such as the geographic distribution of studies, the type of review conducted, the topics addressed, the type of data analysis employed, and the tools used in the research processes. These results were cross-referenced and represented through graphs to identify key trends and patterns in the reviewed literature.

To facilitate the localization and management of references, all articles analyzed have been compiled in Supplementary Table S1. This table serves as a centralized repository, enabling readers to efficiently access and manage the references used throughout this study.

Regarding the type of review, the articles included systematic reviews and meta-analyses, showing how each approach contributed to the synthesis of knowledge. Additionally, the use of different types of data analysis was evaluated, classifying them into qualitative, quantitative, and mixed approaches. This categorization revealed a wide variety of methodologies, reflecting the diversity in approaches applied to address research problems.

As this is a review of reviews, the analyzed studies collectively comprise more than 33,000 individual studies, as outlined in column N of Supplementary Table S2. The column provides specific counts for each review, allowing a detailed exploration of their scope. On average, each review analyzed around 88 studies, reflecting the substantial breadth of these reviews. The analysis of the age groups of participants in the studies reviewed by the selected reviews reveals a diverse focus. For example, around 110 reviews targeted adults, while 130 focused on youth or young populations. Reviews involving children accounted for 32 entries, and 93 reviews included a mixed age range. This categorization, presented in column 6 of Supplementary Table S2, highlights the broad demographic scope of the reviewed studies.

A thorough analysis of the main topics addressed in the articles was conducted, including areas such as clinical psychology, educational psychology, social psychology, and health-related studies. Within educational psychology, recurring constructs included “learning styles” and “educational outcomes,” as explored in studies like Clinton-Lisell & Litzinger and Dreer. Social psychology frequently addressed themes such as “social identity” and “social integration,” as highlighted in Hu & Cheung, as well as “social sustainability” and “social connectedness,” discussed in studies by Kobal Grum & Babnik and Petersen et al. Health-related studies commonly focused on constructs like “mental health” and its interplay with factors such as “digital impact” (Chen et al.) and other psychological dimensions, as examined by Limone and Toto.

Each construct and topic analyzed for the reviewed articles can be verified in Supplementary Table S2, where all relevant details are documented for transparency and further exploration. This table organizes information across different categories, providing a comprehensive view of the approaches and characteristics of the articles included in the analysis. Columns in the supplementary table include essential data such as the study title, year of publication, country of origin, and continent, allowing for an exploration of the geographic distribution of the investigations.

This supplementary table serves as a valuable resource for readers, offering detailed access to the information that supports the general analysis presented in this work. Its structure enables the identification of patterns, the exploration of specific trends, and a deeper examination of the particular characteristics of the included studies, providing a solid foundation for future research and academic synthesis.

To facilitate the categorization of the reviewed studies, the identified constructs were grouped into overarching topics based on an iterative analytical process. The categories were not predefined but rather emerged from a systematic dataset analysis, through multiple rounds of review, refining and consolidating the thematic structure. This approach enabled the classification of studies published in Frontiers in Psychology into distinct topical categories that best captured their focus while aligning with existing research in educational psychology.

The overarching topics were defined through a structured dataset review, with initial classifications iteratively refined to ensure consistency. This process involved examining recurring themes, research objectives, and conceptual frameworks across studies. Consequently, the final categorization represents a data-driven synthesis, integrating patterns and thematic clusters identified in the dataset.

Intersections between different psychological domains (e.g., clinical, social, cognitive, or educational psychology) were determined based on the stated objectives of each study. Instead of imposing arbitrary classifications, interdisciplinary connections were identified through an in-depth examination of how constructs from various fields contributed to psychoeducational research. This method ensured that interdisciplinary relationships were grounded in the focus and intent of each study, rather than in an externally imposed taxonomy.

The geographic analysis considered the distribution of studies by continents and regions, distinguishing between Eastern and Western contexts. This approach facilitated the observation of how research priorities and methodologies varied across regional contexts. It allowed the identification of significant differences in predominant areas of interest and methodological approaches between the studied regions.

For geographic regions, distinguishing between East and West posed challenges, particularly in cases where studies involved authors from multiple regions. In such instances, articles were counted once for each relevant region, which may impact the totals. Detailed information on excluded categories and their respective studies is provided in the Supplementary materials, offering a comprehensive and transparent account of the dataset. This approach ensures that the analysis remains focused on the most relevant and widely adopted methodologies and typologies.

Finally, the use of tools and software proved to be fundamental for data analysis and management. Their increasingly frequent application highlights a significant trend toward the adoption of advanced technologies and artificial intelligence within the field of scientific and psychological research. This shift underscores the growing integration of digital methodologies in academic investigations, reflecting an evolution in how systematic reviews are conducted. The generated graphs visualized these interrelationships and trends over time and by region, providing a comprehensive perspective of the obtained results.

The discrepancy between the total number of analyzed studies and the figures presented in the graphs arises from several methodological considerations. First, only meta-analyses and systematic reviews have been included in the typology analysis, excluding other review types with significantly smaller representation (Aslaksen & Lorås; Burris & Brown). Similarly, the focus on qualitative, quantitative, and mixed methodologies for data analysis reflects their predominance within the dataset, while other approaches were considered residual due to their limited occurrence and scope (Di; Tinajero et al.). Additionally, the software analysis only included studies explicitly reporting the use of digital tools, further narrowing the scope.

### 3.1 Comparative analysis by type of review

A detailed comparison was conducted between studies employing systematic reviews and those utilizing meta-analysis, enabling the exploration of methodological and thematic differences between both approaches. This classification is available in Supplementary Table S3, where each sheet contains articles classified according to the type of review. This analysis identified key patterns related to the number of studies, the tools employed, and the types of data analysis applied in each case.

**Figure 3:**
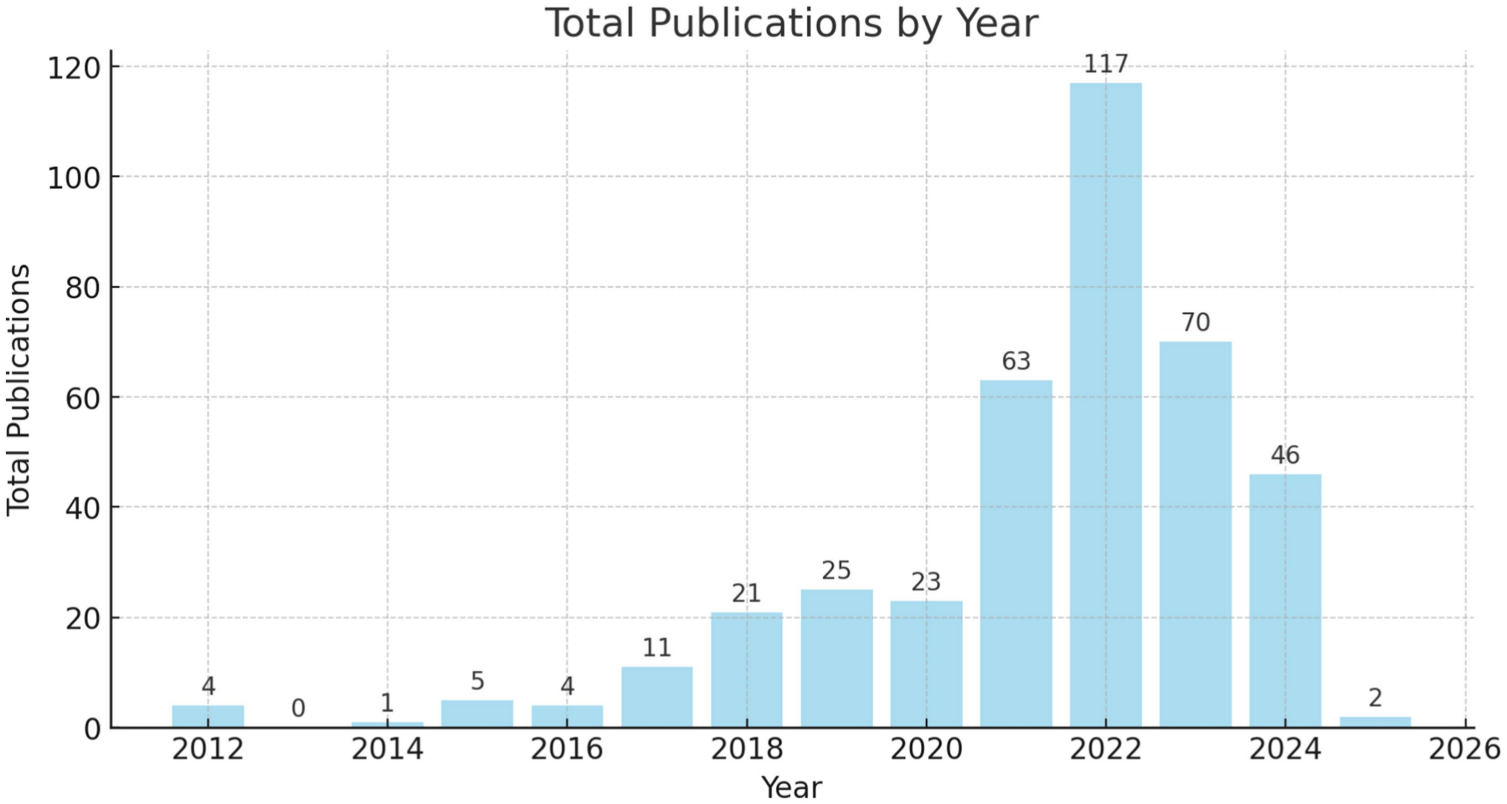
Sustained increase in publications over time. This figure shows the consistent growth in the number of publications.

**Figure 4:**
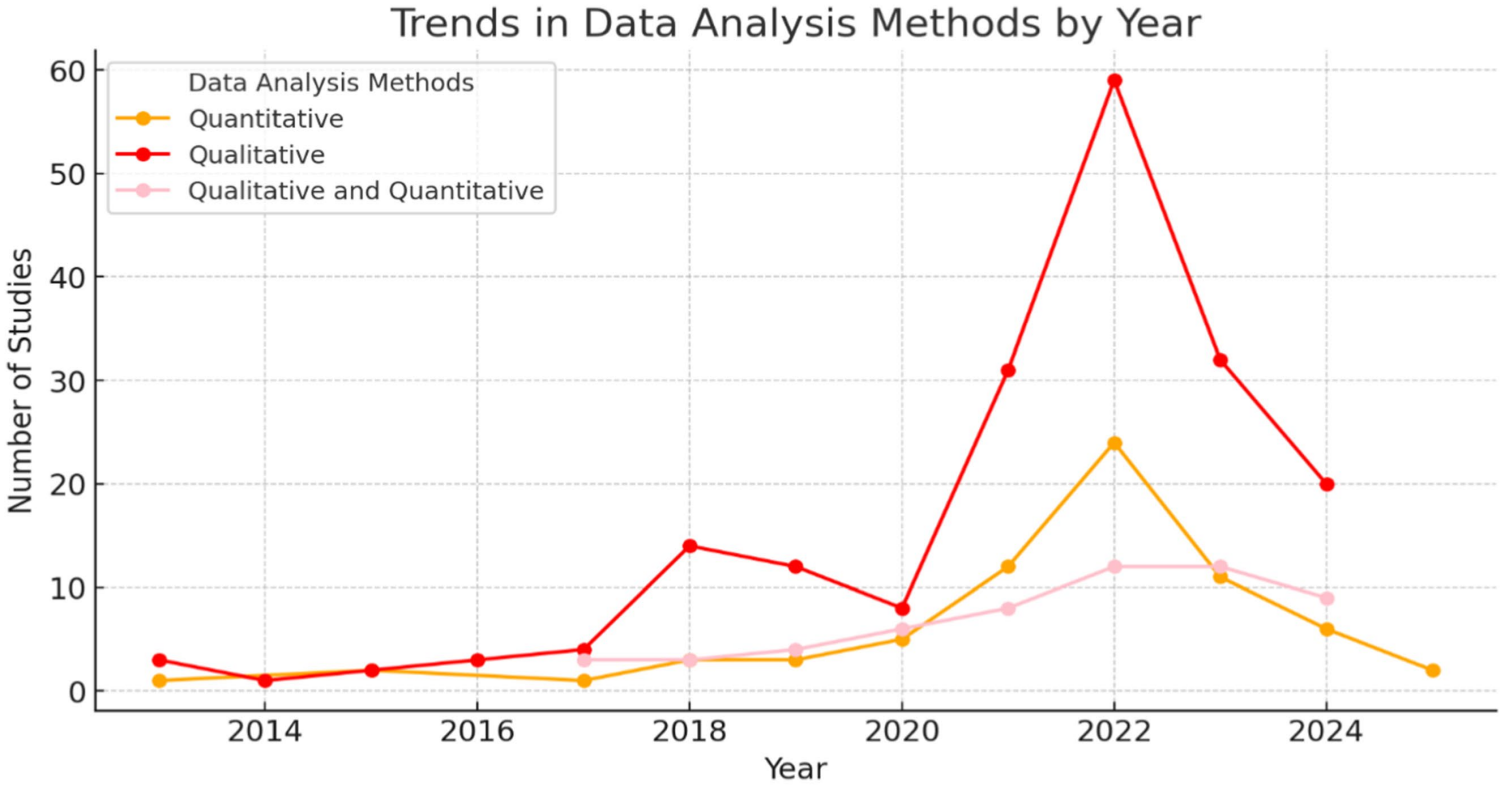
Evolution of methodologies used in systematic reviews and meta-analysis. The figure demonstrates trends in the adoption of qualitative, quantitative, and mixed methods over time.

Figure 3 demonstrated a sustained increase in the number of publications over time, reaching its highest point in recent years. The temporal distribution revealed marked growth in both systematic reviews and meta-analysis, indicating a general increase in interest in these methodologies within the scientific literature (Hobbs et al.; Kritikou & Giovazolias; Lauder et al.). This increase was more pronounced for systematic reviews, although meta-analysis also exhibited a growing trend, as observed in Figure 4.

Specifically, Figure 4 highlighted an evolution in the methodologies employed. Systematic reviews have increased the use of qualitative and mixed approaches in recent years (Akram et al.; Amores-Valencia et al.; Kuznetsova et al.), while meta-analysis have maintained a prevalence in the use of quantitative analysis analysis (Carrus et al.; Jinmin & Qi; Subara-Zukic et al.). These trends have allowed the identification of variations in methodological approaches over time, contributing to a better understanding of research practices in both types of review.

The temporal distribution of systematic reviews and meta-analyses also reveals a significant peak in publications during 2022. This increase aligns with broader scientific trends observed in the post-pandemic period, where researchers faced restrictions on conducting empirical studies in educational and psychological settings. As a result, systematic reviews became a preferred methodological approach, allowing scholars to synthesize and evaluate accumulated knowledge in the absence of direct experimental data.

**Figure 5:**
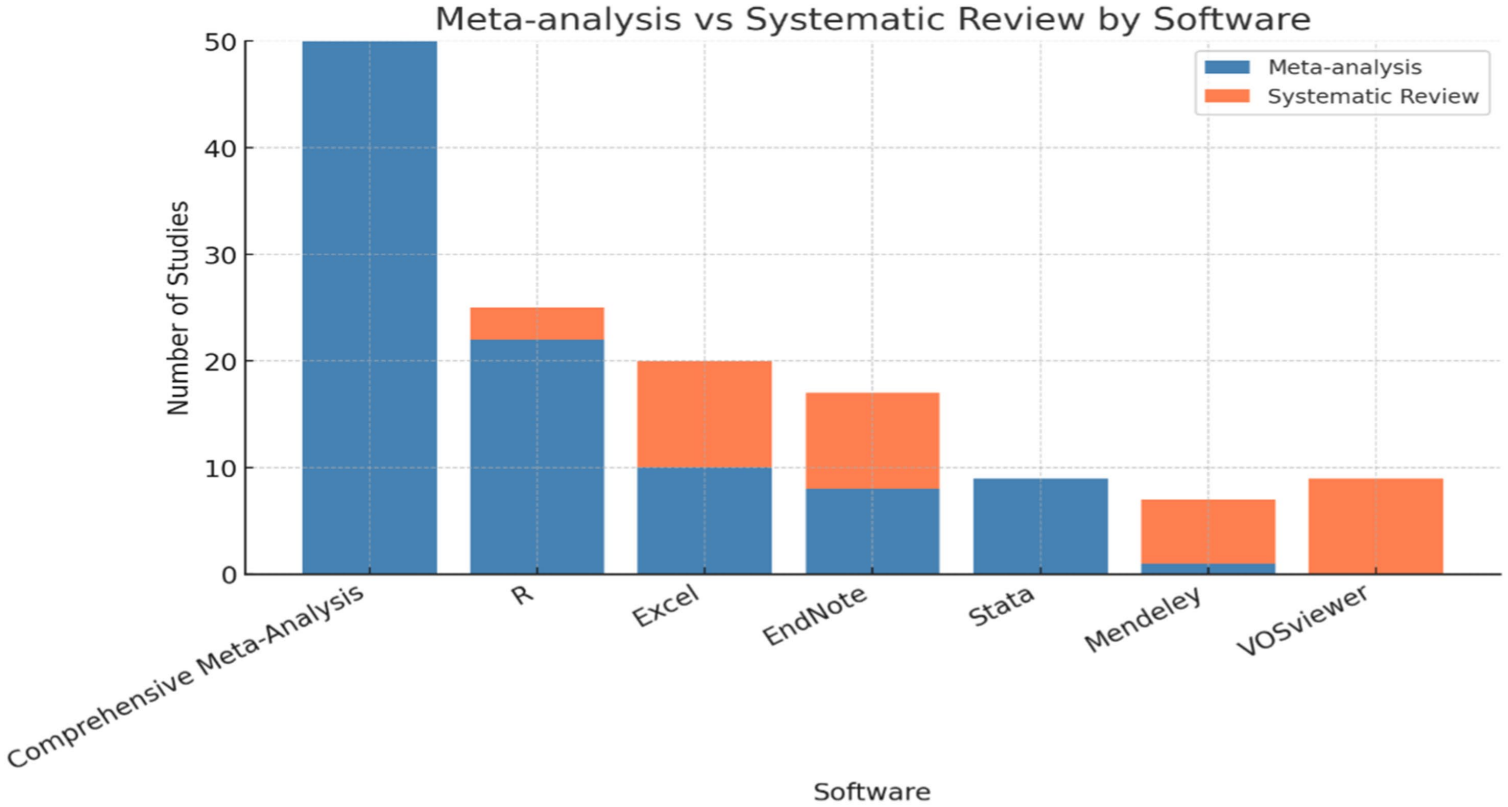
Software usage trends by year. This chart highlights the increasing adoption of digital tools in research.

Regarding the software utilized, Figure 5 highlighted differences in the tools employed for each type of review. Meta-analysis predominantly used statistical programs such as Comprehensive Meta-Analysis and R (Amlashi et al.; Andersen et al.; Chen), while systematic reviews demonstrated greater diversity, incorporating tools like EndNote and Excel for data management and organization (Beaudoin et al.; Bi et al.; Sharif Nia et al.). These differences in software usage reflected the distinct methodological demands of each type of review, which were influenced by the nature of the analysis conducted.

### 3.2 Analysis by studied topic

A detailed analysis was conducted on the studies grouped by main topics, categorized into seven areas: Clinical Psychology, Cognitive Psychology, Developmental Psychology, Educational Psychology, Health Psychology, Quantitative Psychology, and Social Psychology. These data are available in Supplementary Table S4, presented in tables detailing the articles included in each topic. This approach allowed the identification of key trends in research areas, their evolution over time, and their relationship with other methodological and regional factors.

This categorization by topics aims to facilitate comparisons and provide a clearer understanding of the research landscape. While grouped under broader topics, the specific constructs studied in each article can be consulted in the Supplementary material, ensuring detailed access to the nuances of the research. This level of granularity allows for a more comprehensive interpretation of the findings.

Although the articles span diverse topics, they share a connection to educational psychology, either directly or through overlapping constructs such as well-being, motivation, or cognitive development. This thematic overlap justifies their inclusion in the study, as it highlights the interdisciplinary nature of educational psychology and its intersection with other areas of psychological research.

**Figure 6:**
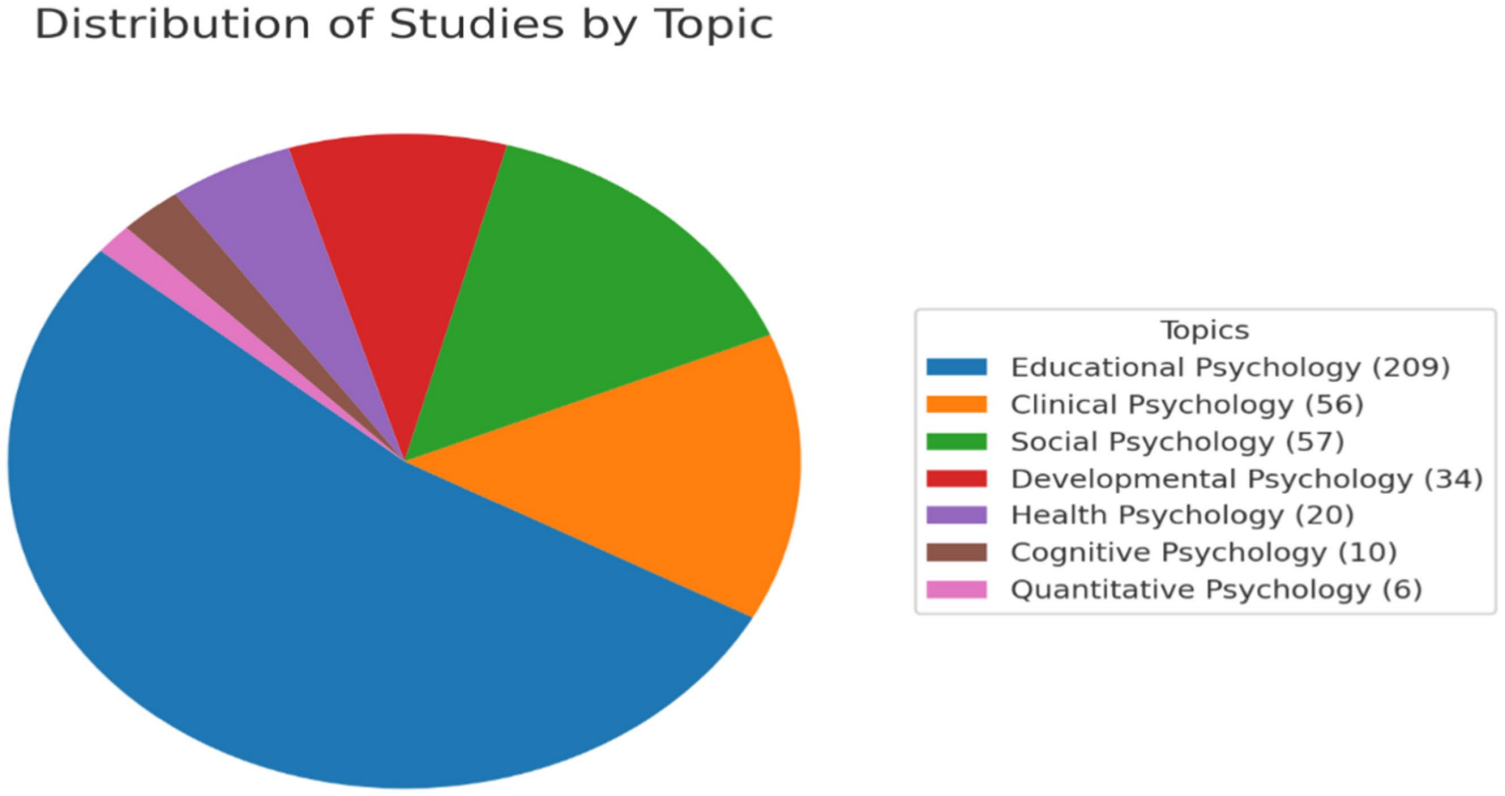
Distribution of publications by research topic. The figure shows the predominance of topics.

Figure 6 revealed a predominant distribution in the topic of Educational Psychology (Aslaksen & Lorås; Attwood; Di), followed by Social Psychology and Clinical Psychology, which together encompassed the majority of the analyzed studies (Andersen et al.; Salgado & Moscoso; Tokuhama-Espinosa et al.). Topics with lesser representation included Quantitative Psychology and Health Psychology, reflecting a concentration of research efforts in specific areas (Peña-Sarrionandia et al.; Pérez-Fernández et al.; Sáiz-Manzanares et al.).

**Figure 7:**
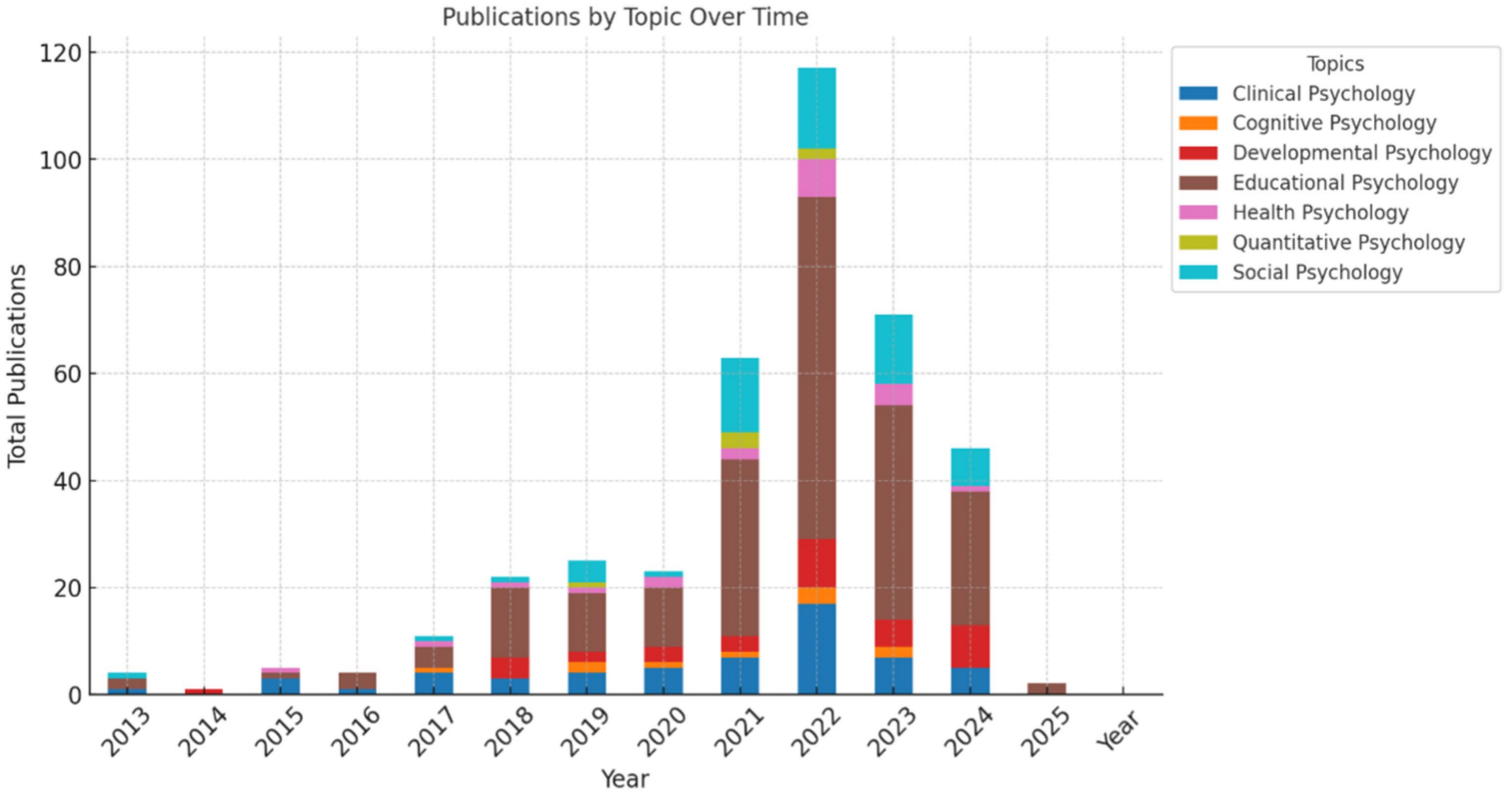
Temporal growth of publications across topics. This chart reveals the progressive increase in publications.

In the analysis by years, Figure 7 evidenced a progressive increase in the number of publications across all topics, with a significant peak in recent years, particularly in Educational Psychology and Social Psychology (O’Grady & Nag; Para et al.; Xu & Wang). This increase has been consistent with the growing interest in these research areas, while other topics, such as Quantitative and Cognitive Psychology, have shown more moderate growth (Aryadoust et al.; Bono et al.; Gegenfurtner).

**Figure 8:**
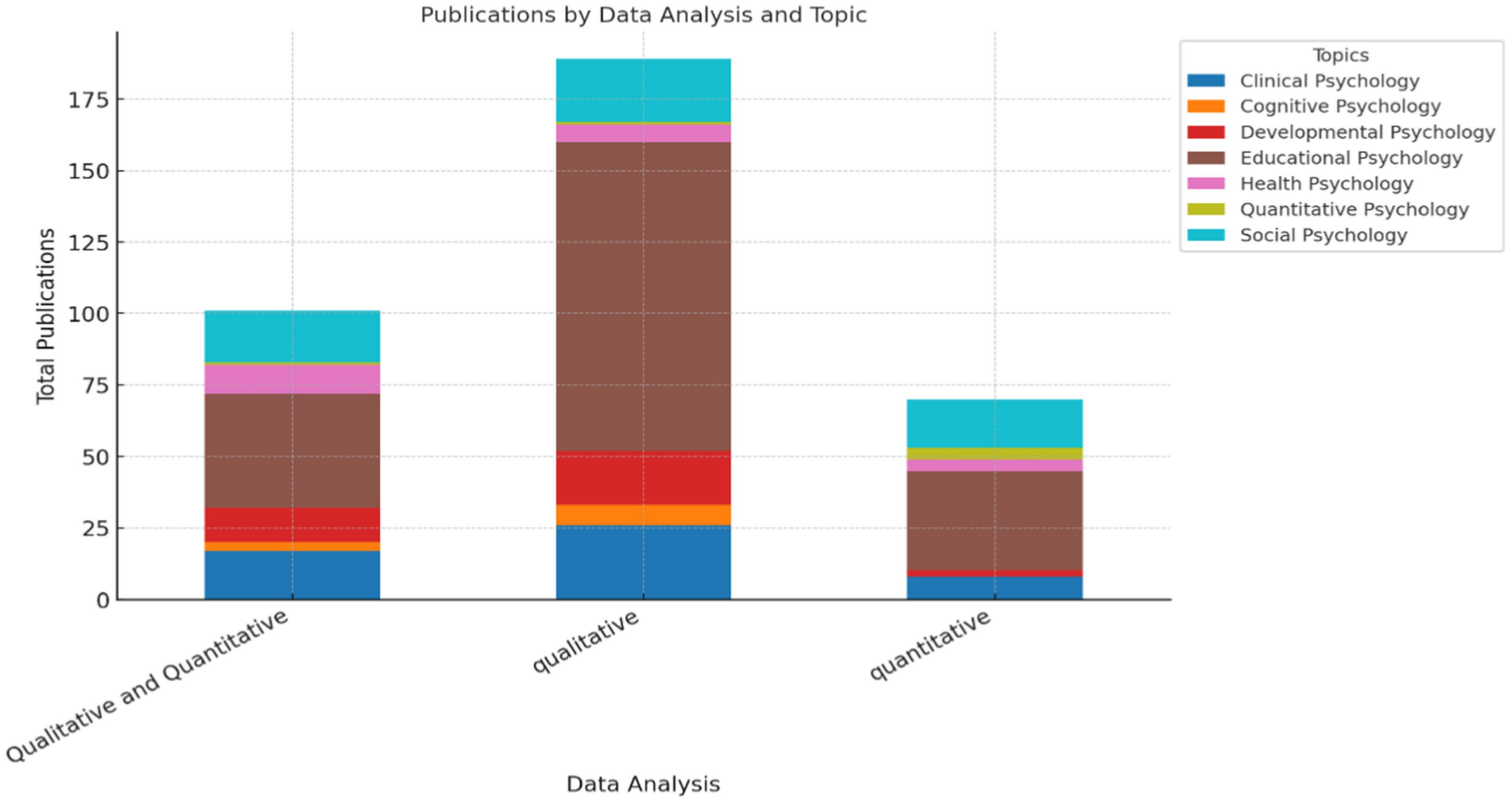
Types of data analyses by research topic. The figure analyses the prevalence of qualitative, quantitative, and mixed methods across different research areas.

The analysis of data analysis types (Figure 8) revealed a clear predominance of qualitative approaches, especially in Clinical Psychology, which stood out significantly compared to other topics (Dumont et al.; Gao; Nadmilail et al.). In contrast, quantitative methods were more dominant in Social Psychology. Notably, Educational Psychology was prominently represented in both qualitative and quantitative approaches, as evidenced by the substantial segment sizes in both categories (Fuller et al.; Hancock et al.; Zhou et al.). Mixed approaches (qualitative and quantitative) were generally less frequent than qualitative but remained more common than quantitative in certain fields, particularly in Health Psychology, where their presence indicated a greater methodological diversity (He et al.; Marino & Capone; Sánchez-López et al.). Overall, while qualitative methodologies were the most widely used, the chart illustrates a notable variation in methodological preferences across different topics, with some fields demonstrating a stronger inclination toward mixed-method research.

### 3.3 Comparative analysis by geographic regions

To provide a comprehensive understanding of geographical trends, this study employs two complementary classification approaches: the East–West regional division and the continent-based analysis. The East–West classification was chosen to group research traditions that transcend political borders, capturing historical and methodological influences that shape the prevalence of Systematic Reviews and Meta-analyses in different regions. This approach allows for an understanding of broader scientific paradigms, highlighting distinct methodological preferences, such as the qualitative emphasis in the West versus the statistical rigor observed in the East.

Specifically, for the purposes of this study, the East–West division was operationalized as follows: “West” includes all countries in North, Central, and South America, Western Europe, Oceania, and Western Africa; whereas “East” encompasses Asia, Eastern Europe, Russia, the Middle East, and Eastern Africa.

Conversely, the continent-based classification was applied to analyze temporal growth and thematic distribution, as this framework provides a more granular and widely recognized geographical categorization. This distinction also explains why Systematic Reviews vs. Meta-analyses were not analyzed by continent—since the methodological divide observed is primarily driven by scientific traditions rather than geographical boundaries. Unlike research output trends or thematic priorities, which vary considerably by continent, methodological preferences tend to be structured around historical, epistemological, and institutional factors that are better captured by an East–West division.

Similarly, the annual publication trends and topic distribution were not analyzed using an East–West classification because such an approach would have limited explanatory power in these contexts. The number of publications and their thematic focus are shaped more by national and institutional research funding, policy changes, and global academic collaborations, which are more accurately reflected when analyzed at the continental level. Applying an East–West division to these analyses would not provide additional insights, as the primary variations occur at a regional and national level rather than between broad research traditions.

By incorporating both classifications where they are most methodologically relevant, this study ensures a macro-level comparison of methodological preferences while also facilitating a micro-level exploration of research dynamics across continents. This dual perspective enhances the depth and accuracy of the findings while preserving conceptual clarity in the interpretation of geographical trends.

**Figure 9:**
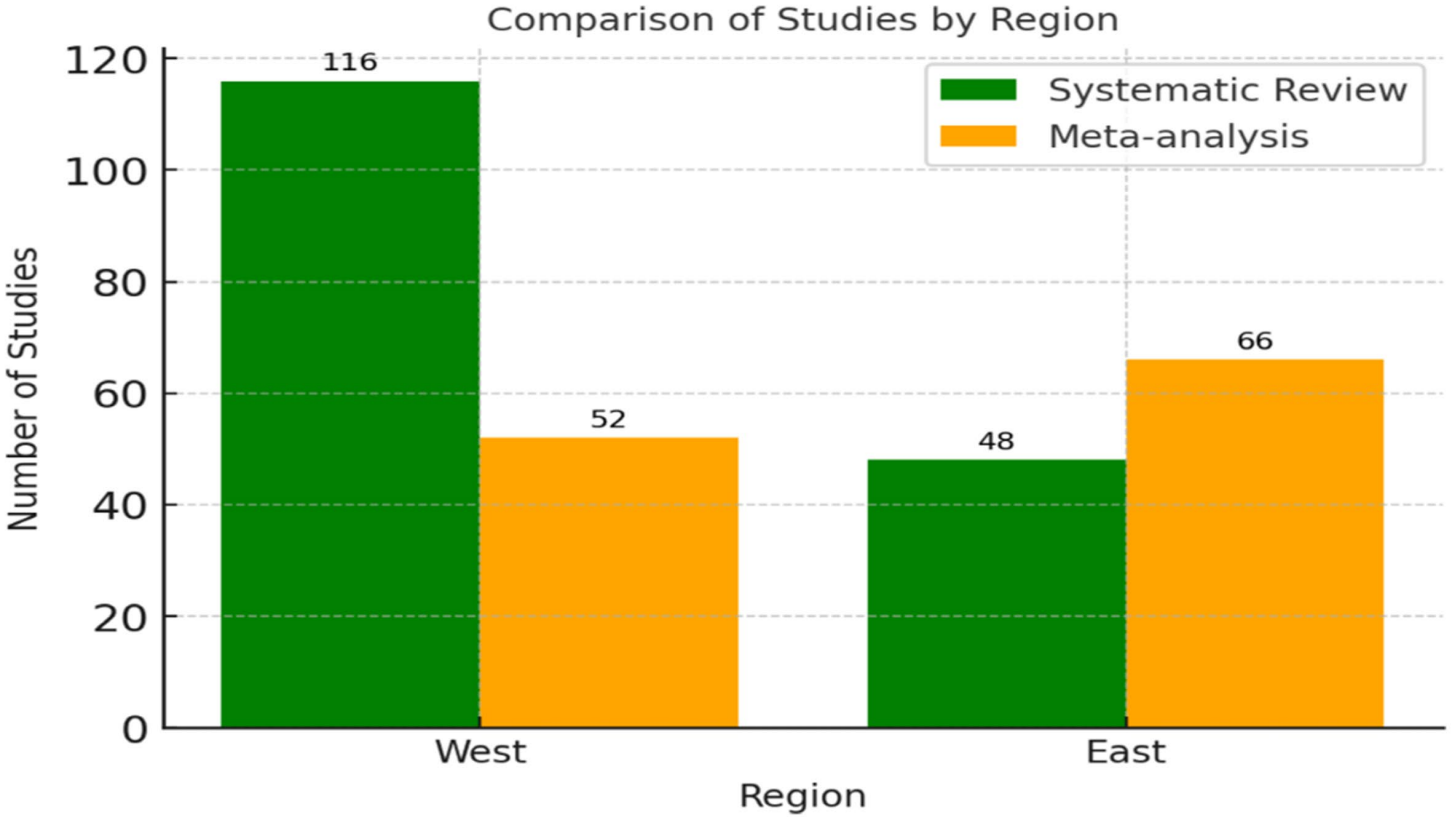
Distribution of systematic reviews and meta-analysis by region. This figure compares the prevalence of systematic reviews and meta-analysis in Eastern and Western regions.

The general analysis of the reviewed studies revealed geographical differences across various analyzed focuses (Supplementary Table S5). Methodologically, the distribution of studies between Systematic Reviews and Meta-analyses in the East and West regions was highlighted (Figure 9).

In the West, Systematic Reviews were significantly more prevalent, with a total of 116 studies, clearly surpassing Meta-analyses, which accounted for 52 (Delfa-Lobato et al.; Hammerstein et al.; Lisboa et al.). Conversely, in the East, Meta-analyses were more numerous, reaching 66 studies compared to 48 Systematic Reviews (Bolton et al.; Gong et al.; Tang & He). This pattern highlights a regional preference in research methodologies, with the West favoring a more qualitative and comprehensive approach, while the East leans toward a more quantitative and statistical perspective.

**Figure 10:**
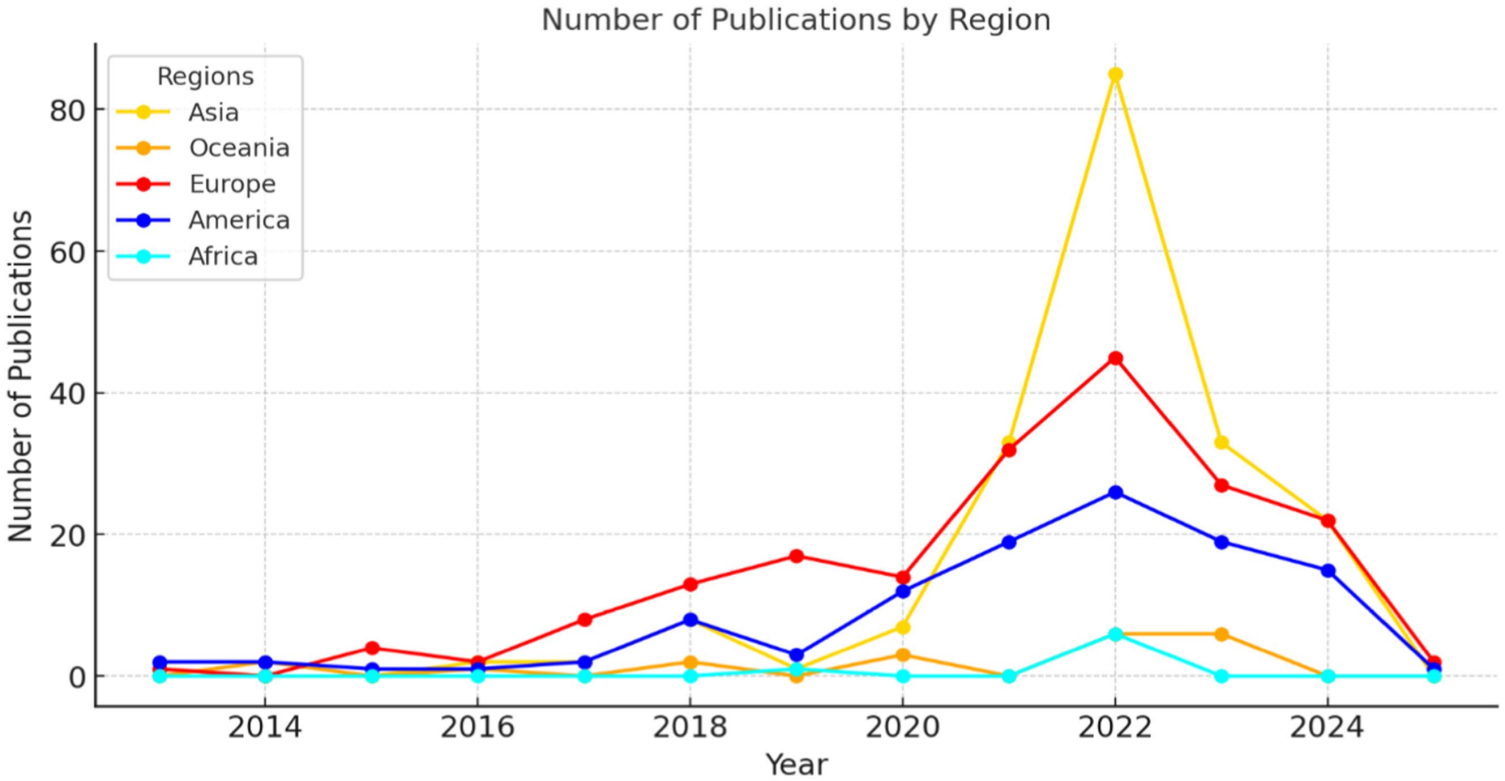
Yearly and regional distribution of publications. The figure highlights the steady growth in research activity across continents.

Furthermore, the distribution of publications by year and continent showed sustained growth in Europe and Asia, with notable peaks in recent years that reflected an increase in research activity in these regions, primarily in 2022 (Shi et al.; Tronchoni et al.; Wang & Wang). America, although maintaining a consistent production, lagged behind Europe and Asia (Bravo-Sanzana et al.; Jebb et al.; Lentz et al.), whereas Africa and Oceania exhibited a much more limited representation (McLean et al.; Orth et al.; Ware et al.). This highlighted the centralization of academic output in regions with greater resources and infrastructure for research (Figure 10).

**Figure 11:**
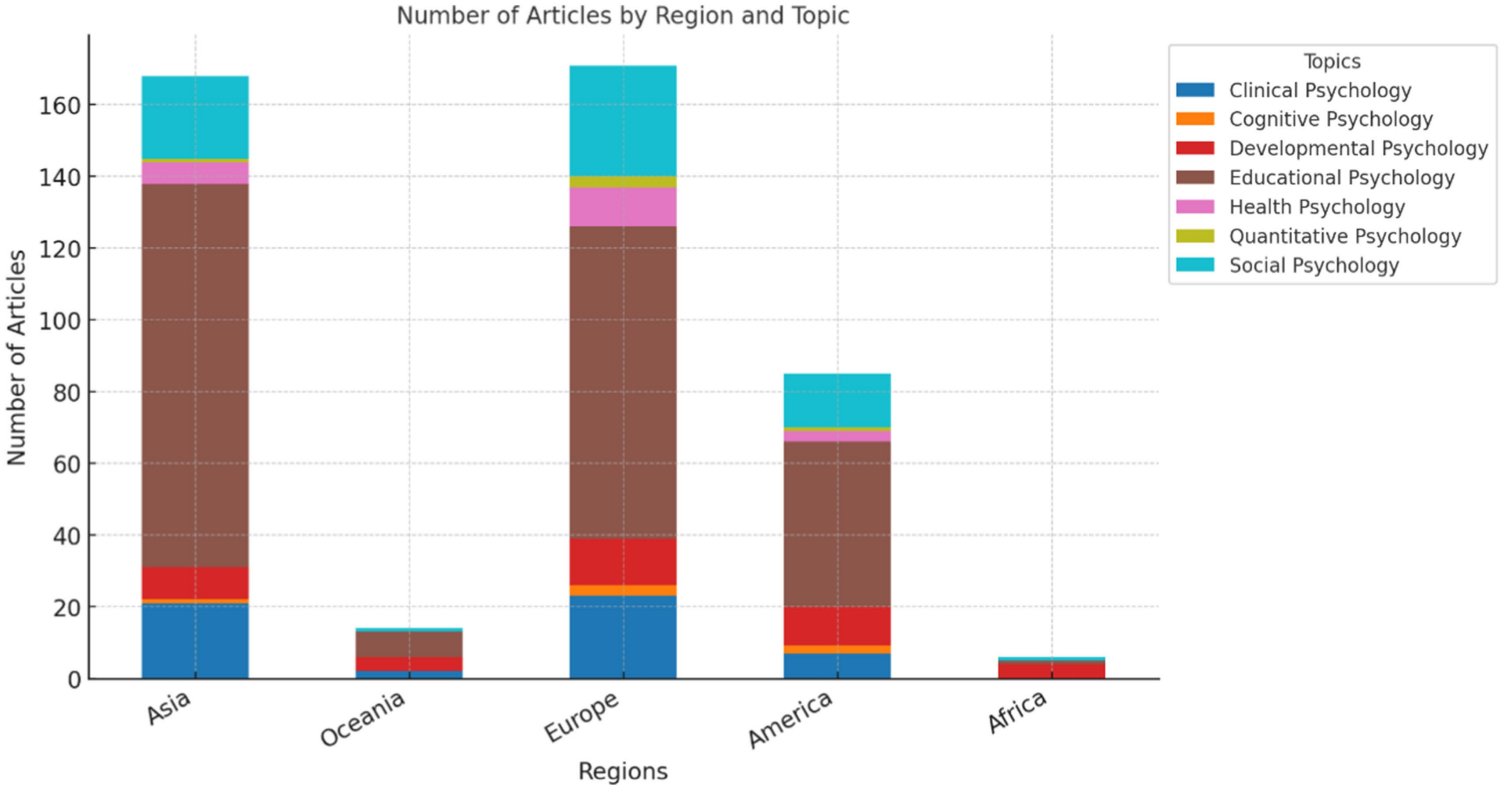
Key research topics by continent. This visualization categorizes research topics into six main areas, showing the dominant focus by continent.

Finally, the thematic analysis by continent revealed clear differences in research priorities. Europe led in the number of studies, with a strong emphasis on Social, Clinical and Educational Psychology (Beer & Mulder; Dreer; Maier et al.). Asia ranked second, also demonstrating a significant focus on Social and Educational Psychology (Aziku & Zhang; Jianping et al.; Yoon et al.). America showed a notable emphasis on Educational Psychology, reflecting a strong interest in educational and social psychological topics (Molina et al.; Clinton-Lisell & Litzinger; Kenny et al.). Africa and Oceania had a smaller representation, with fewer studies distributed across different topics, but without a clearly dominant area (Annous et al.; Frantz et al.; Zhao & Wang; Figure 11).

### 3.4 Software analysis

The analysis of software usage was conducted considering the growing presence of digital tools in scientific reviews and the consequent importance of acquiring digital competencies. The specific use of each software can be found in Supplementary Table S2, which includes dedicated columns recording the programs employed in each reviewed article.

**Figure 12:**
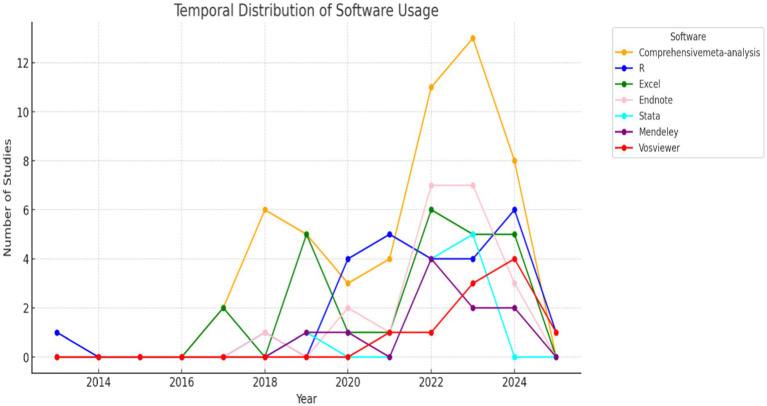
Software adoption trends over time. This visualization explores the growing use of digital tools.

Figure 12, which illustrates software usage by year, evidenced a significant increase in the adoption of digital tools in recent years. Notably, the consistent and predominant use of Comprehensive Meta-Analysis (CMA) peaked in 2022–2023, followed by a decline in 2024 (Chen et al.; Jiang et al.; Ni et al.). Concurrently, the use of R has shown an increasing trend since 2018, with fluctuations but remaining one of the primary tools in recent years (Haberstroh & Schulte-Körne; Sanchez-Alvarez et al.; Zhan et al.). Other tools, such as EndNote and Excel, exhibited specific peaks in usage, indicating their relevance in data management and organization for systematic reviews (Finell et al.; Pit-ten Cate & Glock; Vigdal & Brønnick).

The graph depicts the frequency of software usage for data analyzis in reviewed studies, categorized by year. Data were extracted from the “Software” column, with each software mentioned in a cell separated by “/” being counted individually. For example, if a cell contains “Excel/R,” both Excel and R received one count each. Instances marked as “Not Specific” were excluded from the analysis. The results illustrate the evolution and trends in software adoption over the years, with notable peaks for Comprehensive Meta-Analysis and other tools reflecting methodological preferences.

**Figure 13:**
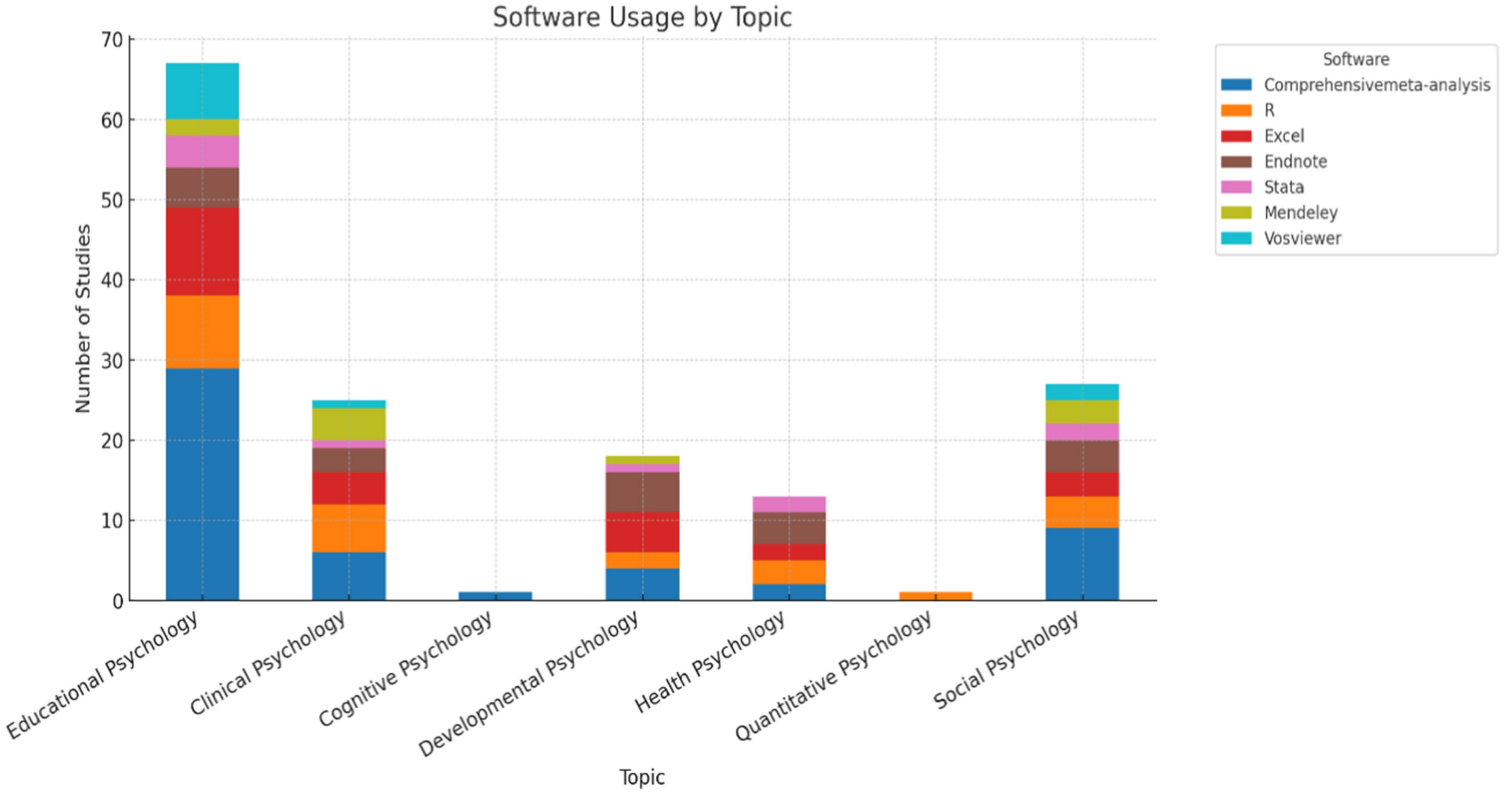
Software usage by research topic. The figure links specific software preferences to research topics.

The analysis in Figure 13, which links software usage to the investigated topics. The distribution of software usage across topics reveals that Comprehensive Meta-Analysis (CMA) is the predominant tool, particularly in Educational Psychology, where it has the highest number of studies. In Social Psychology, CMA also holds a strong presence, followed by R, which is widely used across multiple topics, including Clinical Psychology (Kievit et al.; Xu & Xue; Xuan et al.). Excel and EndNote show notable peaks in Clinical and Educational Psychology, reflecting their role in data management for systematic reviews. Meanwhile, Stata appears in a smaller number of studies, with representation across multiple topics but a higher presence in Clinical and Educational Psychology (Kenny et al.; Llistosella et al.). This distribution suggests a relationship between the methodological demands of specific topics and the tools used to address them.

**Figure 14:**
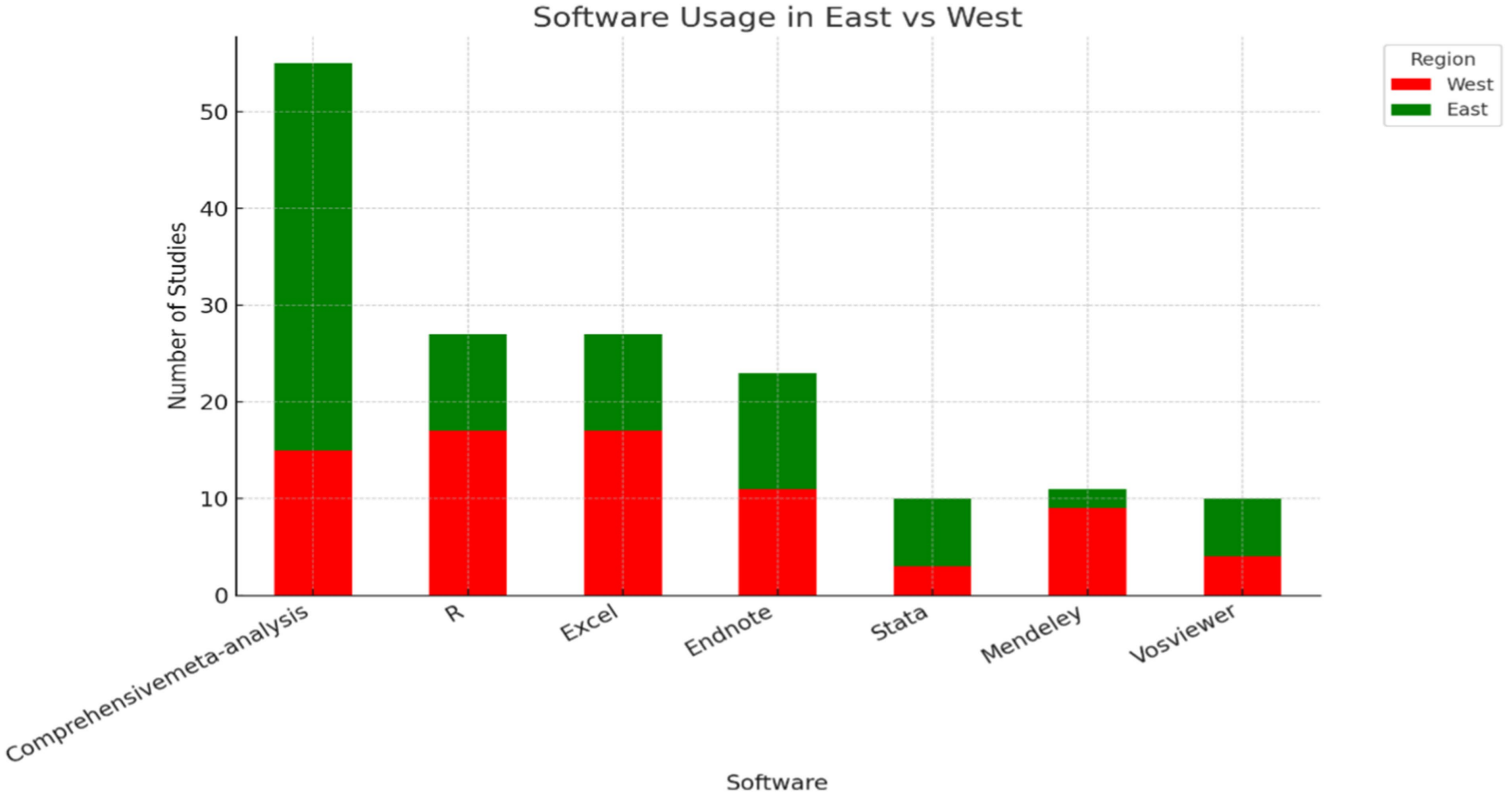
Regional software preferences. This chart highlights the differences in software usage between Eastern and Western regions.

Figure 14 shows the distribution of software usage. Comprehensive Meta-Analysis (CMA) is the most used tool, particularly in the East, where it surpasses its usage in the West. R is also widely employed in both regions, though slightly more in the West (Pascual et al.; Smale-Jacobse et al.; Xu & Xue). While Excel and EndNote have similar usage in both East and West, their presence suggests an integration of qualitative and organizational analysis processes (Cao et al.; Norouzkhani et al.; Peng). This indicates that both regions value technology in their research, albeit with different emphases. Meanwhile, Stata has a lower representation in both regions, reinforcing its role as a complementary rather than primary tool (Ding et al.; Yu et al.).

### 3.5 Special issue

The special issue, comprising a collection of studies, presented an innovative and cohesive approach that stood out in several key aspects compared to other reviews (Supplementary Table S2). These works, in an integrated manner, addressed educational sustainability through theoretical and practical approaches that promoted inclusive and adaptable development in the fields of education and psychology. Additionally, they emphasized collaborative models and highlighted the impact of familial and social contexts on learning. This framework provided a unified perspective that transcended traditional boundaries, integrating social, emotional, and educational aspects.

A core theme was the emphasis on self-regulated learning and instructional strategies to enhance academic achievement. For example, research by Shao et al. demonstrated how scaffolding techniques significantly improved students’ ability to manage their own learning, fostering autonomy and better performance. This study highlights the importance of structured support systems in helping students develop independent learning habits, ultimately leading to better academic outcomes.

A second important connection among the studies was the implementation of inclusive and innovative teaching methodologies. Cochon Drouet et al. highlighted the benefits of the Jigsaw method in fostering social relationships and increasing academic motivation through peer collaboration. Their findings suggest that collaborative pedagogical approaches not only improve academic engagement but also enhance students’ social integration, reinforcing the value of inclusive learning environments.

Another significant link was the development of creativity as a fundamental educational outcome. Fan et al. provided evidence that parental involvement plays a crucial role in fostering creativity in children and adolescents. Their study emphasizes how family dynamics contribute to creative growth, illustrating that creativity is not solely developed within academic settings but also shaped by external social factors.

The special issue also addressed the psychosocial factors influencing academic persistence and well-being. De La Fuente & Martínez Vicente introduced the Conceptual Model of Stress Management and Psychological Well-being (CMMSPW™), linking stress regulation to overall mental health in education. Their model suggests that effective stress management strategies can serve as protective factors against academic burnout, helping students maintain long-term engagement with their studies.

Finally, it examined career readiness and adaptability in professional contexts, linking educational development to long-term career success. Wang & Li assessed vocational adaptability and professional identity, providing insights into tools and interventions that enhance career trajectories. Their findings highlight the importance of developing flexible career-oriented skills early in education to ensure students can effectively transition into professional roles.

Overall, the special issue distinguished itself through its interdisciplinary approach, focusing on inclusive and practical solutions to address challenges in education and psychology. By integrating innovative methodologies, psychoeducational variables, and fostering collaboration across diverse contexts, the collection advanced the understanding of key trends and set the stage for future research in these critical areas.

## 4 Discussion

### 4.1 Key findings and novelty

This study provides a distinctive contribution to the field of educational psychology by conducting a systematic review of reviews, an approach that represents a significant methodological innovation in the field. Traditional systematic reviews and meta-analyses, while valuable, often focus on synthesizing empirical findings within a specific domain, limiting their scope to individual studies or a narrowly defined research question. In contrast, a review of reviews enables a higher-order synthesis, integrating insights from multiple systematic reviews and meta-analyses to identify overarching trends and methodological patterns that would otherwise remain fragmented across different studies (Branquinho et al., 2021; Furley and Goldschmied, 2021; Topping, 2022).

As advancements in psychology and education accelerate to address increasingly complex societal challenges, comprehensive meta-research becomes essential for contextualizing existing knowledge and evaluating the evolution of methodologies and theoretical frameworks. While systematic reviews and meta-analyses are widely used in educational psychology, reviews of reviews remain significantly less frequent, despite their potential to provide a broader and more integrative perspective on the field. Unlike traditional systematic reviews that aggregate empirical data, this study systematically examines authors and years of publication, countries or regions of origin, review types, age groups, constructs addressed, main topics, theoretical frameworks, digital tools, reliability, validation, review periods, databases, quality analyses, methodologies, data analysis techniques, added value, results, limitations, and applications. By integrating findings across multiple reviews, this research not only maps emerging patterns but also establishes a roadmap for future priorities, refining theoretical frameworks and methodological approaches in educational psychology. This approach encompasses key focal points such as publication trends, regional influences, review typologies, methodological rigor, and the role of digital tools in evidence synthesis, ensuring a nuanced understanding of how research practices evolve over time (Lange-Smith et al., 2024; Stier-Jarmer et al., 2021; Oswald et al., 2024).

At the methodological level, the study highlights the role of structured data analysis in synthesizing complex research trends. The use of Excel for database structuring enabled a systematic confrontation of multiple focal points, revealing significant insights into how psychoeducational and economic factors shape research priorities over time and across regions. This methodological approach underscores the importance of leveraging digital tools to enhance meta-research while maintaining rigorous analytical frameworks.

Due to the strict analysis, the general objective of this study—to conduct a comparative analysis of reviews published in *Frontiers in Psychology* by identifying predominant characteristics, methodologies, trends, and regional patterns—has been achieved by systematically categorizing reviews across typology, thematic, methodological, and geographical dimensions. The review utilized a multi-layered approach, analyzing studies to uncover methodological trends and providing examples that validate the diversity and rigor of psychological research.

**Figure 15:**
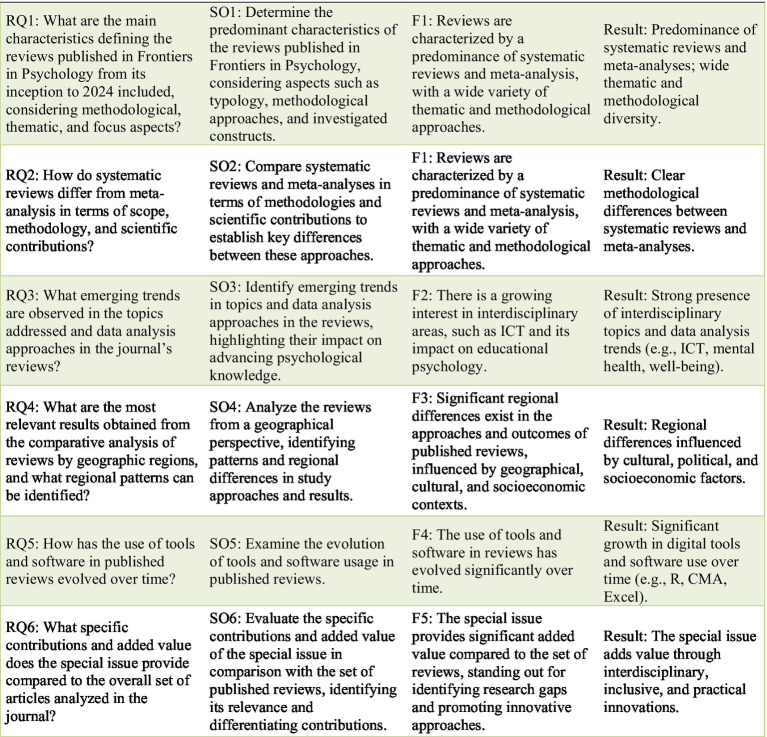
Summary table aligning the research questions (RQ), specific objectives (SO), forecasts (F), and main results of the study.

The findings not only address the study’s overarching goals but also provide a foundation for understanding how psychological research aligns with global challenges. This sets the stage for a more detailed analysis of the specific objectives, research questions, and forecasts, which will be examined individually in the following paragraphs (Figure 15).

The results of this study show a broad and comprehensive characterization of the reviews published in *Frontiers in Psychology*, a leading journal in the field with a high Journal Citation Reports (JCR) impact factor, addressing RQ1 and achieving SO1. These reviews span a wide variety of topics, including educational psychology, teacher well-being, and the integration of digital literacy, reflecting the journal’s commitment to addressing contemporary challenges in psychology. The study highlights a clear predominance of systematic reviews and meta-analysis, which provide rigorous frameworks for analyzing and synthesizing information. For example, Aziku and Zhang conducted a systematic review of research on teacher well-being during the COVID-19 pandemic, highlighting trends in methodology and focus areas. Meanwhile, Clinton-Lisell and Litzinger performed a meta-analysis on the learning styles matching hypothesis, assessing the empirical evidence for its effectiveness. These findings showcase a rich methodological diversity suitable for interdisciplinary challenges. This methodological emphasis demonstrates the maturity of psychological research and its ability to adapt to interdisciplinary demands. By categorizing reviews by their thematic focus, methodological approaches, and investigated constructs, this study offers a nuanced understanding of the trends and priorities in the field, validating F1 by showing the diversity and evolution of research practices.

The observed increase in systematic reviews, particularly in 2022, reflects broader shifts in scientific inquiry during and after the COVID-19 pandemic. This global event represented a turning point in history, not only for society but also for scientific research, prompting scholars to reassess existing knowledge. Given the disruptions to in-person research activities, many researchers turned to systematic reviews as a means to consolidate and analyze the accumulated body of work up to that moment. The objective was to understand how this historical milestone had influenced scientific paradigms, methodologies, and research priorities. The trend highlights the adaptability of systematic reviews as a research strategy capable of navigating exceptional circumstances, ensuring continuity in scientific production, and facilitating evidence-based decision-making. Moreover, the increasing reliance on systematic reviews suggests a paradigm shift in research methodology, where synthesizing and critically analyzing prior studies has become as essential as generating new empirical data, allowing researchers to reflect on the profound impact of the pandemic on the evolution of knowledge.

The comparison between systematic reviews and meta-analysis addresses RQ2 and contributes to achieving SO2, validating F1 by highlighting fundamental differences in scope and methodology. Systematic reviews, with their flexibility, adapt to broad and exploratory questions, as seen in Amores-Valencia et al., who explored how Augmented Reality (AR) influences academic motivation and performance in secondary education. Meanwhile, meta-analyses, such as Shao et al., focus on synthesizing quantitative data, evaluating the impact of regulated learning scaffolding strategies on self-regulated learning and academic outcomes. These differences reflect their complementary roles in advancing psychological research, enabling it to address interdisciplinary challenges while meeting the growing demand for robust scientific evidence.

The thematic diversity observed in published reviews reflects an effort to adapt to current challenges in psychology. Areas such as educational psychology and teacher well-being have gained significant relevance, likely due to their close connection with global issues like digital transformation and the need to promote well-being in high-demand contexts. Fan et al. conducted a three-level meta-analysis, showing that parental involvement positively predicts student creativity, with autonomy support and behavioral control having the most significant effects. Meanwhile, Chen et al. explored the role of digital technology in promoting mental health among children and adolescents, highlighting the effectiveness of interventions like mobile applications, VR, and serious games in addressing depression, anxiety, and other mental health challenges. This thematic focus addresses RQ3 and achieves SO3, as anticipated in F2, underscoring how reviews in *Frontiers in Psychology* strategically position themselves to address global priorities in psychological research.

The comparative analysis by regions addresses RQ4 and achieves SO4, validating F3 by highlighting how geographical, cultural, and socioeconomic factors influence research approaches and priorities. For example, the predominance of educational psychology in Asia reflects government policies that prioritize education as a key driver of economic growth, particularly in countries like China, Japan, and South Korea, where high-stakes testing systems and rigorous academic expectations shape research agendas. This emphasis aligns with a broader cultural and economic focus on early educational interventions, which aim to build foundational skills essential for academic success and workforce competitiveness.

In contrast, the focus on educational and developmental psychology in America appears to be driven by policies that integrate psychological research into both healthcare and education systems. The strong emphasis on mental health and student well-being reflects public concerns about social–emotional learning, neurodevelopmental disorders, and the psychological impacts of education policies, which are actively addressed through federal funding programs and interdisciplinary research initiatives. Additionally, psychology research in America shows a significant presence of social psychology, which may be linked to the region’s focus on issues such as equity, diversity, and inclusion within educational contexts.

Meanwhile, European research stands out for its long-term commitment to equity, inclusion, and sustainability in education, aligning with EU policies that promote lifelong learning and social cohesion. This emphasis is evident in research priorities that focus on educational accessibility, multicultural education, and psychological interventions aimed at reducing disparities. The diversity of research topics across these regions demonstrates how local priorities, cultural values, and institutional frameworks shape psychological inquiry, influencing both research questions and methodological approaches. Consequently, reviews in *Frontiers in Psychology* address both global trends and region-specific challenges, ensuring a nuanced understanding of how educational psychology adapts to different societal needs.

For example, Cao et al. conducted a meta-analysis on computer-based training programs aimed at enhancing children’s executive functions, reinforcing Asia’s emphasis on early cognitive development as a foundation for academic success and economic growth. In Europe, Dreer highlights the integration of educational and social psychology to address equity, inclusion, and sustainability challenges, aligning with EU policies on lifelong learning and social cohesion. Attwood analyzed the role of multiple intelligences theory in American education, highlighting how evidence-based frameworks integrate psychological research into instructional design. This reinforces America’s focus on student well-being, inclusion, and the psychological foundations of education.

Regarding software and tools, the findings address RQ5 and achieve SO5, confirming F4. The increasing adoption of programs such as R and Comprehensive Meta-Analysis reflects significant progress in researchers’ digital competencies, while the continued use of tools like Excel highlights the persistence of qualitative and organizational approaches in systematic reviews. This development underscores the shift toward digital tools and AI-driven methodologies, aligning with broader trends in technological innovation in educational psychology. Chen exemplifies this shift by conducting a meta-analysis on self-regulated learning interventions, demonstrating the increasing sophistication of statistical methods in educational research. However, traditional approaches remain relevant, as evidenced by studies like Beaudoin et al., which emphasize systematic reviews in developmental psychology, reflecting the methodological diversity in psychological research.

The special issue represents a significant milestone in educational psychology, offering a cohesive and integrative framework that distinguishes it from prior reviews. By blending psychoeducational variables with collaborative and inclusive methodologies, it emphasizes the interplay between familial, social, and emotional contexts. This holistic approach creates actionable frameworks to address real-world challenges in psychoeducation, effectively bridging gaps between theory and practice.

Addressing RQ6 and achieving SO6, as anticipated in F5, the special issue’s unified approach underscores its role in fostering sustainable and adaptable interventions across diverse educational settings. Unlike prior reviews focusing on isolated aspects, this issue integrates emotional regulation, collaborative learning, and innovative methodologies, aligning with contemporary educational psychology trends. Its comprehensive synthesis offers a roadmap for implementing interdisciplinary solutions that respond to modern educational and psychological complexities.

For instance, Kuznetsova et al. explore giftedness assessment through cognitive, psychological, and cultural dimensions, advocating for more holistic identification methods. Meanwhile, Hurtado et al. (2024) examine doctoral student persistence through a multi-factorial framework addressing individual, academic, and institutional challenges. Collectively, these contributions illustrate the special issue’s relevance in advancing adaptable, evidence-based educational solutions.

The results and analyses presented in this review align with the principles established by the Sustainable Development Goals (SDGs), particularly SDG 4: Quality Education. By categorizing and analyzing thematic and methodological trends, the study reflects a commitment to promoting equitable and accessible education. The focus on educational psychology and the integration of diverse approaches underscores the importance of tailoring educational strategies to cultural and socioeconomic conditions, further advancing inclusive and equitable education.

Moreover, this research indirectly supports SDG 3: Good Health and Well-being by emphasizing strategies that promote emotional regulation and collaborative learning. The findings highlight the role of psychological health in fostering supportive and effective educational environments. The special issue’s cohesive framework reinforces the connection between educational outcomes and mental health by integrating psychoeducational and emotional variables.

As part of this shift toward evidence-based and technology-enhanced education, AI-driven methodologies are increasingly being integrated into psychological and educational research. These tools offer potential advantages, such as accelerating systematic reviews, improving data analysis, and identifying research gaps more efficiently. However, while AI-powered tools have improved efficiency, their use also raises critical challenges.

One major limitation is the lack of transparency in AI-based decision-making, particularly in automated article selection, bias detection, and quality assessment, which could lead to the unintentional exclusion of relevant studies or the amplification of pre-existing biases in research. Additionally, current AI tools still face significant gaps in semantic comprehension and contextual analysis, making it difficult to accurately interpret complex psychological constructs and theoretical frameworks. As a result, human oversight remains essential to ensure accuracy, reliability, and ethical integrity in AI-assisted reviews.

Moving forward, AI should be integrated into educational psychology research as a complement rather than a replacement for human analysis, with a focus on developing hybrid models that combine AI efficiency with expert validation. Strengthening algorithmic transparency, improving contextual understanding, and refining bias mitigation strategies will be crucial to enhancing the reliability of AI-driven research tools.

Through its exploration of digital and AI methodologies, this study also aligns with SDG 8: Decent Work and Economic Growth and SDG 9: Industry, Innovation, and Infrastructure. By showcasing methodological advancements and evolving research tools, it underscores the importance of preparing the workforce for a digital research environment while ensuring that AI adoption remains ethically sound and methodologically rigorous. The integration of advanced research tools reflects a growing emphasis on leveraging technological progress to enhance educational and psychological research.

### 4.2 Limitations and future directions

The structured database developed in Excel provides a versatile and efficient resource for organizing and analyzing data, offering extensive opportunities for future research. This tool enables detailed meta-analyses (e.g., examining the number of studies reviewed and their participants), classification of study types (e.g., interventions, developmental studies, program validations), and exploration of methodological trends across time or regions. These capabilities reinforce the foundational value of this study, allowing psychoeducational research to advance by identifying key trends and gaps.

Beyond its methodological applications, this study highlights the practical, theoretical, and technological implications of systematic reviews. Methodologically, it underscores the importance of comparative analyses to synthesize findings and refine research frameworks. Theoretically, it provides a framework for understanding emotional regulation, equity, and global priorities in psychoeducational research. Practically, its findings offer insights into designing action plans to address emerging challenges, such as those posed by ICT, digitalization, and AI, within the context of the Sustainable Development Goals.

To further advance scientific knowledge in psychoeducational research, future studies should expand the methodological diversity of systematic reviews and meta-analyses. In particular, intervention-based studies assessing the practical impact of educational policies and psychological frameworks could provide valuable insights. Longitudinal studies tracking how research trends evolve would help contextualize shifts in methodologies and thematic priorities. Additionally, as digitalization and AI continue to shape education, research should examine their influence on learning processes, teacher training, and student outcomes. Finally, cross-cultural comparative analyses could deepen our understanding of how educational psychology reviews address global challenges and adapt to different socio-economic contexts. Integrating these research directions will enrich the field and ensure systematic reviews remain central to addressing contemporary educational and psychological challenges.

Despite these innovations, this study has certain limitations. First, it focuses exclusively on reviews published in *Frontiers in Psychology* related to educational psychology, which may limit the generalizability of the findings to other journals or psychological domains. However, to mitigate potential selection biases, a triangulation strategy was employed by conducting searches in Web of Science (WOS) and Scopus databases, ensuring a comprehensive retrieval of relevant studies. While this approach enhances methodological rigor, future research could expand the scope by incorporating systematic reviews from multiple journals, enabling comparative analyses of editorial policies, methodological approaches, and thematic trends across different academic platforms. Additionally, meta-analyses could be conducted to quantitatively synthesize findings from a wider range of sources, providing deeper insights into evolving research trends in educational psychology.

Furthermore, regarding the representativeness of the included studies, it is important to acknowledge that while this review captures a significant portion of systematic reviews and meta-analyses in educational psychology, it does not encompass the full diversity of global research output. Variations in institutional research priorities, funding availability, and regional publication trends may influence the distribution and methodological approaches of the studies analyzed. Future research could address this by incorporating cross-journal comparisons and exploring geographic, institutional, and disciplinary variations in the development of systematic reviews and meta-analyses within the field.

By encouraging further exploration of these trends and patterns, this study provides a lasting contribution to the advancement of knowledge and practice. The structured database developed in this research represents a key resource for uncovering new insights, conducting innovative analyses, and driving interdisciplinary research in response to global challenges.

## 5 Conclusion

This study underscores the pivotal contributions of reviews related to education in advancing psychology, emphasizing their capacity to synthesize extensive knowledge, uncover patterns, and address interdisciplinary challenges. By conducting a systematic review of reviews, this research introduces a methodological innovation that reflects the rapid evolution of psychology in addressing the complexities of contemporary educational and societal needs. The study’s approach highlights the necessity of analyzing prior reviews to map trends, identify gaps, and provide a comprehensive synthesis that guides future research. This added value not only positions the study as a significant contribution to the field but also sets a precedent for similar efforts across related disciplines.

The novelty of this study lies in its ability to offer a critical, comparative analyses across multiple dimensions, including typology, thematic focus, regional perspectives, and methodological approaches. By systematically categorizing reviews published in *Frontiers in Psychology*, this research highlights the importance of synthesizing diverse findings to keep pace with the rapid advancements in psychoeducational research. Such an approach is essential in a field as dynamic as psychology, where the constant emergence of new challenges and priorities necessitates innovative strategies for synthesizing existing knowledge.

The inclusion of the special issue within *Frontiers in Psychology* underscores the journal’s commitment to advancing the field by addressing pressing global challenges through innovative and interdisciplinary approaches. By fostering inclusivity and adaptability, this special issue complements the broader contributions of this study, offering actionable insights and advancing sustainable solutions for contemporary educational and psychological needs.

This study achieves its main objective of systematically analyzing reviews related to education, offering a comprehensive synthesis that aligns with the Sustainable Development Goals (SDGs), particularly SDG 4 (Quality Education) and SDG 3 (Good Health and Well-being). By addressing themes such as emotional regulation, inclusive practices, and educational sustainability, the findings emphasize the need for interdisciplinary strategies that promote equity and well-being in diverse psychoeducational contexts. Additionally, the study’s forward-looking perspective underscores the growing importance of transitioning towards digital and AI-driven methodologies, highlighting their potential to revolutionize how educational systems adapt to global challenges.

In summary, this study highlights the value of conducting reviews of reviews as a methodological innovation in psychology. It provides a robust foundation for future research by demonstrating how these meta-analyzes can synthesize complex findings and guide global strategies in education. This research not only enriches academic discourse but also motivates researchers and practitioners to adopt innovative and inclusive approaches, fostering sustainable and impactful solutions that address the evolving needs of educational psychology in a rapidly changing world.

## Data Availability

The raw data supporting the conclusions of this article will be made available by the authors, without undue reservation.

## Supplementary material

SUPPLEMENTARY TABLE S1 References of all analyzed studies.

SUPPLEMENTARY TABLE S2 General analysis with additional details and comparative data from the call of paper.

SUPPLEMENTARY TABLE S3 Analysis of articles split into two sheets: systematic review and meta-analyses.

SUPPLEMENTARY TABLE S4 Overview of research topics and their categorization.

SUPPLEMENTARY TABLE S5 Analysis of articles divided into Eastern and Western publications.
