# Supplementary material for: Reviews in Educational Psychology (Frontiers in Psychology 2010–2024): typology, topics, regional comparative and methodology toward digital and AI approaches
Source: Front Psychol. 2025 Apr 30;16:1588242. doi: 10.3389/fpsyg.2025.1588242 (PMC12257778; doi:10.3389/fpsyg.2025.1588242)
Supplement: SUPPLEMENTARY TABLE S1 — References of all analyzed studies. [file Table_1.docx]

Reviews in Educational Psychology (Frontiers in Psychology 2010-2024): typology, topics, regional comparative and methodology. Towards digital and AI approaches.

**Alberto Díaz-Burgos^1*^, Jesús N García-Sánchez^1*^, María-Lourdes Álvarez-Fernández^1*^, Sónia Brito-Costa^2,3*,^ Douglas F Kauffman^4^, Ting-Chia Hsu^5^, Jesús de la Fuente^6^.**

^1^ Universidad de León, 24071 León, Spain, ^2^ Instituto Politécnico de Coimbra, Education School, 3045-093 Coimbra, Portugal, ^3^INED - Center for Research and Innovation in Education, Polytechnic Institute of Porto, Porto, Portugal, ^4^School of Clinical Medicine, Medical University of America-Nevis, Devens, MA, United States, ^5^Department of Technology Application and Human Resource Development, National Taiwan Normal University, Taipei City 10610, Taiwan^,^  ^6^School Education and Psychology, University of Navarra, Pamplona, Spain.

References of reviews analyzed papers

*Aguilar D and Pifarre Turmo M (2019) Promoting Social Creativity in Science Education With Digital Technology to Overcome Inequalities: A Scoping Review. *Front. Psychol*. 10:1474. [doi:10.3389/fpsyg.2019.01474](https://doi.org/10.3389/fpsyg.2019.01474)

*Aithal S, Moula Z, Karkou V, Karaminis T, Powell J and Makris S (2021) A Systematic Review of the Contribution of Dance Movement Psychotherapy Towards the Well-Being of Children With Autism Spectrum Disorders. *Front. Psychol.* 12:719673. [doi: 10.3389/fpsyg.2021.719673](https://doi.org/10.3389/fpsyg.2021.719673).

*Akram H, Abdelrady AH, Al-Adwan AS and Ramzan M (2022) Teachers’ Perceptions of Technology Integration in Teaching-Learning Practices: A Systematic Review. *Front. Psychol.* 13:920317. [doi:10.3389/fpsyg.2022.920317](https://doi.org/10.3389/fpsyg.2022.920317).

*Alabbasi AMA, Paek SH, Kim D and Cramond B (2022) What do educators need to know about the Torrance Tests of Creative Thinking: A comprehensive review. *Front. Psychol*. 13:1000385. [doi:10.3389/fpsyg.2022.1000385](https://doi.org/10.3389/fpsyg.2022.1000385)

*Alonzo D, Oo CZ, Wijarwadi W and Hannigan C (2023) Using social media for assessment purposes: Practices and future directions. *Front. Psychol*. 13:1075818. [doi:10.3389/fpsyg.2022.1075818](https://doi.org/10.3389/fpsyg.2022.1075818)

*Álvarez-Godos M, Ferreira C and Vieira MJ (2023) A systematic review of actions aimed at university students with ADHD. *Front. Psychol*. 14:1216692. [doi:10.3389/fpsyg.2023.1216692](https://doi.org/10.3389/fpsyg.2023.1216692)

*Amores-Valencia A, Burgos D and Branch-Bedoya JW (2022) Influence of Motivation and Academic Performance in the Use of Augmented Reality in Education: A Systematic Review. *Front. Psychol.* 13:1011409. [doi:10.3389/fpsyg.2022.1011409](https://doi.org/10.3389/fpsyg.2022.1011409).

*Andersen FB, Djugum MET, Sjåstad VS and Pallesen S (2023) The Prevalence of Workaholism: A Systematic Review and Meta-Analysis. *Front. Psychol.* 14:1252373. [doi:10.3389/fpsyg.2023.1252373](https://doi.org/10.3389/fpsyg.2023.1252373).

*Annous N, Al-Hroub A and El Zein F (2022) A Systematic Review of Empirical Evidence on Art Therapy With Traumatized Refugee Children and Youth. *Front. Psychol.* 13:811515. [doi:10.3389/fpsyg.2022.811515](https://doi.org/10.3389/fpsyg.2022.811515).

*Aryadoust V, Tan HAH and Ng LY (2019) A Scientometric Review of Rasch Measurement: The Rise and Progress of a Specialty. *Front. Psychol.* 10:2197. [doi:10.3389/fpsyg.2019.02197](https://doi.org/10.3389/fpsyg.2019.02197).

*Aryadoust V, Zakaria A, Lim MH and Chen C (2020) An Extensive Knowledge Mapping Review of Measurement and Validity in Language Assessment and SLA Research. *Front. Psychol.* 11:1941. [doi:10.3389/fpsyg.2020.01941](https://doi.org/10.3389/fpsyg.2020.01941).

*Ashraf MA, Mollah S, Perveen S, Shabnam N and Nahar L (2022) Pedagogical Applications, Prospects, and Challenges of Blended Learning in Chinese Higher Education: A Systematic Review. *Front. Psychol*. 12:772322. [doi:10.3389/fpsyg.2021.772322](https://doi.org/10.3389/fpsyg.2021.772322)

*Aslaksen K and Lorås H (2018) The Modality-Specific Learning Style Hypothesis: A Mini-Review. *Front. Psychol.* 9:1538. [doi:10.3389/fpsyg.2018.01538](https://doi.org/10.3389/fpsyg.2018.01538).

*Asperholm M, van Leuven L and Herlitz A (2020) Sex Differences in Episodic Memory Variance. *Front. Psychol.* 11:613. [doi:10.3389/fpsyg.2020.00613](https://doi.org/10.3389/fpsyg.2020.00613).

*Attwood AI (2022) A Conceptual Analysis of the Semantic Use of Multiple Intelligences Theory and Implications for Teacher Education. *Front. Psychol.* 13:920851. [doi:10.3389/fpsyg.2022.920851](https://doi.org/10.3389/fpsyg.2022.920851).

*Azevedo R, Bouchet F, Duffy M, Harley J, Taub M, Trevors G, Cloude E, Dever D, Wiedbusch M, Wortha F and Cerezo R (2022) Lessons Learned and Future Directions of MetaTutor: Leveraging Multichannel Data to Scaffold Self-Regulated Learning With an Intelligent Tutoring System. *Front. Psychol*. 13:813632 [doi:10.3389/fpsyg.2022.813632](https://doi.org/10.3389/fpsyg.2022.813632)

*Aziku M and Zhang B (2024) Systematic review of teacher well-being research during the COVID-19 pandemic. *Front. Psychol.* 15:1427979. [doi:10.3389/fpsyg.2024.1427979](https://doi.org/10.3389/fpsyg.2024.1427979).

*Ballesteros-Quilez J, Rivera-Vargas P and Jacovkis J (2022) Counter hegemony, popular education, and resistances: A systematic literature review on the squatters’ movement. *Front. Psychol*. 13:1030379. [doi:10.3389/fpsyg.2022.1030379](https://doi.org/10.3389/fpsyg.2022.1030379)

*Beaudoin C, Leblanc É, Gagner C and Beauchamp MH (2020) Systematic Review and Inventory of Theory of Mind Measures for Young Children. *Front. Psychol.* 10:2905. [doi:10.3389/fpsyg.2019.02905](https://doi.org/10.3389/fpsyg.2019.02905).

*Beckmann E and Minnaert A (2018) Non-cognitive Characteristics of Gifted Students With Learning Disabilities: An In-depth Systematic Review. *Front. Psychol*. 9:504. [doi:10.3389/fpsyg.2018.00504](https://doi.org/10.3389/fpsyg.2018.00504)

*Beer P and Mulder RH (2020) The Effects of Technological Developments on Work and Their Implications for Continuous Vocational Education and Training: A Systematic Review. *Front. Psychol.* 11:918. [doi:10.3389/fpsyg.2020.00918](https://doi.org/10.3389/fpsyg.2020.00918).

*Bernaras E, Jaureguizar J and Garaigordobil M (2019) Child and Adolescent Depression: A Review of Theories, Evaluation Instruments, Prevention Programs, and Treatments. *Front. Psychol*. 10:543. [doi:10.3389/fpsyg.2019.00543](https://doi.org/10.3389/fpsyg.2019.00543)

*Bi H, Zare S, Kania U and Yan R (2022) A systematic review of studies on connected speech processing: Trends, key findings, and implications. *Front. Psychol.* 13:1056827. [doi:10.3389/fpsyg.2022.1056827](https://doi.org/10.3389/fpsyg.2022.1056827).

*Biasutti M (2015) Pedagogical applications of cognitive research on musical improvisation. *Front. Psychol.* 6:614. [doi:10.3389/fpsyg.2015.00614](https://doi.org/10.3389/fpsyg.2015.00614).

*Björk JM, Bolander P and Forsman AK (2021) Bottom-Up Interventions Effective in Promoting Work Engagement: A Systematic Review and Meta-Analysis. *Front. Psychol.* 12:730421. [doi:10.3389/fpsyg.2021.730421](https://doi.org/10.3389/fpsyg.2021.730421).

*Bodart A, Invernizzi S, Lefebvre L and Rossignol M (2023) Physiological reactivity at rest and in response to social or emotional stimuli after a traumatic brain injury: A systematic review*. Front. Psychol*. 14:930177. [doi:10.3389/fpsyg.2023.930177](https://doi.org/10.3389/fpsyg.2023.930177)

*Bolton B, Rooney RM, Hughes A, Hopkins A and Mancini VO (2023) Systematic review and meta-analysis of the prevention of internalizing disorders in early childhood. *Front. Psychol.* 14:1061825. [doi:10.3389/fpsyg.2023.1061825](https://doi.org/10.3389/fpsyg.2023.1061825).

*Bono R, Alarcón R and Blanca MJ (2021) Report Quality of Generalized Linear Mixed Models in Psychology: A Systematic Review. *Front. Psychol.* 12:666182. [doi:10.3389/fpsyg.2021.666182](https://doi.org/10.3389/fpsyg.2021.666182).

*Bravo-Sanzana MV, Varela J, Terán-Mendoza O and Rodriguez-Rivas ME (2023) Measuring school social climate in Latin America: the need for multidimensional and multi-informant tests – A systematic review. *Front. Psychol.* 14:1190432. [doi: 10.3389/fpsyg.2023.1190432](https://doi.org/10.3389/fpsyg.2023.1190432).

*Bueno D (2019) Genetics and Learning: How the Genes Influence Educational Attainment. *Front. Psychol*. 10:1622. [doi:10.3389/fpsyg.2019.01622](https://doi.org/10.3389/fpsyg.2019.01622)

*Burris SE and Brown DD (2024) When all children comprehend: increasing the external validity of narrative comprehension development research. *Front. Psychol.* 5:00168. doi: [10.3389/fpsyg.2014.00168](https://doi.org/10.3389/fpsyg.2014.00168)

*Caetano T, Pinho MS, Ramadas E, Clara C, Areosa T and Dixe MA (2021) Cognitive Training Effectiveness on Memory, Executive Functioning, and Processing Speed in Individuals With Substance Use Disorders: A Systematic Review. *Front. Psychol*. 12:730165. [doi:10.3389/fpsyg.2021.730165](https://doi.org/10.3389/fpsyg.2021.730165)

*Caetano T, Pinho MS, Ramadas E, Lopes J, Areosa T, Ferreira D and Dixe MdA (2023) Substance abuse and susceptibility to false memory formation: a systematic review and meta-analysis. *Front. Psychol.* 14:1176564 [doi:10.3389/fpsyg.2023.1176564](https://doi.org/10.3389/fpsyg.2023.1176564)

*Canedo-García A, García-Sánchez J-N and Pacheco-Sanz D-I (2017) A Systematic Review of the Effectiveness of Intergenerational Programs. *Front. Psychol*. 8:1882. [doi:10.3389/fpsyg.2017.01882](https://doi.org/10.3389/fpsyg.2017.01882)

*Cao S, Zhou S, Luo Y, Wang T, Zhou T and Xu Y (2022) A review of the ESL/EFL learners’ gains from online peer feedback on English writing. *Front. Psychol.* 13:1035803. doi: [10.3389/fpsyg.2022.1035803](https://doi.org/10.3389/fpsyg.2022.1035803)

*Cao Y, Huang T, Huang J, Xie X and Wang Y (2020) Effects and Moderators of Computer-Based Training on Children’s Executive Functions: A Systematic Review and Meta-Analysis. *Front. Psychol.* 11:580329. [doi:10.3389/fpsyg.2020.580329](https://doi.org/10.3389/fpsyg.2020.580329).

*Carey E, Hill F, Devine A and Szücs D (2016) The Chicken or the Egg? The Direction of the Relationship Between Mathematics Anxiety and Mathematics Performance. *Front. Psychol.* 6:1987. [doi: 10.3389/fpsyg.2015.01987](https://doi.org/10.3389/fpsyg.2015.01987)

*Carrus G, Tiberio L, Mastandrea S, Chokrai P, Fritsche I, Klöckner CA, Masson T, Vesely S and Panno A (2021) Psychological Predictors of Energy Saving Behavior: A Meta-Analytic Approach. *Front. Psychol.* 12:648221. doi:[10.3389/fpsyg.2021.648221](https://doi.org/10.3389/fpsyg.2021.648221)​

*Cawley E, Piazza G, Das RK and Kamboj SK (2022) A systematic review of the pharmacological modulation of autobiographical memory specificity. *Front. Psychol.* 13:1045217. [doi:10.3389/fpsyg.2022.1045217](https://doi.org/10.3389/fpsyg.2022.1045217)

*Červený M, Kratochvílová I, Hellerová V and Tóthová V (2022) Methods of increasing cultural competence in nurses working in clinical practice: A scoping review of literature 2011–2021. *Front. Psychol*. 13:936181 [doi:10.3389/fpsyg.2022.936181](https://doi.org/10.3389/fpsyg.2022.936181)

*Chaguendo-Quintero MA, Quintero-Monjes D, Cuervo MT and Sanabria-Mazo JP (2023) Alterations in executive functions in inmates convicted for violent behavior: a systematic review. *Front. Psychol.* 14:1066474. [doi:10.3389/fpsyg.2023.1066474](https://doi.org/10.3389/fpsyg.2023.1066474)

*Challet-Bouju G, Bruneau M, IGNACE Group, Victorri-Vigneau C and Grall-Bronnec M (2017) Cognitive Remediation Interventions for Gambling Disorder: A Systematic Review. *Front. Psychol*. 8:1961. [doi:10.3389/fpsyg.2017.01961](https://doi.org/10.3389/fpsyg.2017.01961)

*Chang Q (2022) The contribution of a hermeneutic approach to investigate psychological variables in second language acquisition. *Front. Psychol.* 13:1055249. doi: [10.3389/fpsyg.2022.1055249](https://doi.org/10.3389/fpsyg.2022.1055249)

*Chaxiong P, Dimian AF and Wolff JJ (2022) Restricted and repetitive behavior in children with autism during the first three years of life: A systematic review. *Front. Psychol.* 13:986876. [doi:10.3389/fpsyg.2022.986876](https://doi.org/10.3389/fpsyg.2022.986876)

*Chen C, Finne E, Kopp A and Jekauc D (2020) Can Positive Affective Variables Mediate Intervention Effects on Physical Activity? A Systematic Review and Meta-Analysis. *Front. Psychol.* 11:587757. doi: [10.3389/fpsyg.2020.587757](https://doi.org/10.3389/fpsyg.2020.587757)

*Chen H and Yu Y (2022) The impact of social-emotional learning: A meta-analysis in China. *Front. Psychol.* 13:1040522. [doi:10.3389/fpsyg.2022.1040522](https://doi.org/10.3389/fpsyg.2022.1040522)

*Chen J (2022) The effectiveness of self-regulated learning (SRL) interventions on L2 learning achievement, strategy employment and self-efficacy: A meta-analytic study. *Front. Psychol.* 13:1021101. doi: [10.3389/fpsyg.2022.1021101](https://doi.org/10.3389/fpsyg.2022.1021101)

*Chen T, Ou J, Li G and Luo H (2024) Promoting mental health in children and adolescents through digital technology: a systematic review and meta-analysis. *Front. Psychol.* 15:1356554. doi: [10.3389/fpsyg.2024.1356554](https://doi.org/10.3389/fpsyg.2024.1356554)

*Clark I and Dumas G (2016) The Regulation of Task Performance: A Trans-Disciplinary Review. *Front. Psychol.* 6:1862. doi: [10.3389/fpsyg.2015.01862](https://doi.org/10.3389/fpsyg.2015.01862)

*Claxton M, Onwumere J and Fornells-Ambrojo M (2017) Do Family Interventions Improve Outcomes in Early Psychosis? A Systematic Review and Meta-Analysis. *Front. Psychol.* 8:371. doi: [10.3389/fpsyg.2017.00371](https://doi.org/10.3389/fpsyg.2017.00371)

*Clinton-Lisell V and Litzinger C (2024) Is it really a neuromyth? A meta-analysis of the learning styles matching hypothesis. *Front. Psychol.* 15:1428732. doi:[10.3389/fpsyg.2024.1428732](https://doi.org/10.3389/fpsyg.2024.1428732)

*Cortés Pascual A, Moyano Muñoz N and Quílez Robres A (2019) The Relationship Between Executive Functions and Academic Performance in Primary Education: Review and Meta-Analysis. *Front. Psychol.* 10:1582. doi: [10.3389/fpsyg.2019.01582](https://doi.org/10.3389/fpsyg.2019.01582)

*Costa A and Faria L (2018) Implicit Theories of Intelligence and Academic Achievement: A Meta-Analytic Review. *Front. Psychol.* 9:829. doi: [10.3389/fpsyg.2018.00829](https://doi.org/10.3389/fpsyg.2018.00829)

*Costa S and Kuss DJ (2019) Current diagnostic procedures and interventions for Gaming Disorders: A Systematic Review. *Front. Psychol.* 10:578. doi: [10.3389/fpsyg.2019.00578](https://doi.org/10.3389/fpsyg.2019.00578)​

*Cristóvão AM, Candeias AA and Verdasca J (2017) Social and Emotional Learning and Academic Achievement in Portuguese Schools: A Bibliometric Study. *Front. Psychol.* 8:1913. [doi:10.3389/fpsyg.2017.01913](https://doi.org/10.3389/fpsyg.2017.01913)

*Cunha L, Silva D and Maggioli S (2022) Exploring the status of the human operator in Industry 4.0: A systematic review. *Front. Psychol.* 13:889129. [doi:10.3389/fpsyg.2022.889129](https://doi.org/10.3389/fpsyg.2022.889129)

*de la Fuente J, González-Torres MC, Aznárez-Sanado M, Martínez-Vicente JM, Peralta-Sánchez FJ and Vera MM (2019) Implications of Unconnected Micro, Molecular, and Molar Level Research in Psychology: The Case of Executive Functions, Self-Regulation, and External Regulation. *Front. Psychol*. 10:1919. [doi:10.3389/fpsyg.2019.01919](https://doi.org/10.3389/fpsyg.2019.01919)

*de la Fuente J, Kauffman D, Díaz-Orueta U and Kauffman Y (2018) Adapting the Research Development and Innovation (RD & I) Value Chain in Psychology to Educational Psychology Area. *Front. Psychol*. 9:1188. [doi:10.3389/fpsyg.2018.01188](https://doi.org/10.3389/fpsyg.2018.01188)

*de la Fuente J, Kauffman DF, Dempsy MS and Kauffman Y (2021) Analysis and Psychoeducational Implications of the Behavior Factor During the COVID-19 Emergency. *Front. Psychol*. 12:613881. [doi:10.3389/fpsyg.2021.613881](https://doi.org/10.3389/fpsyg.2021.613881)

*de la Fuente J, Martínez-Vicente JM, Santos FH, Sander P, Fadda S, Karagiannopoulou E, Boruchovitch E and Kauffman DF (2022) Advances on Self-Regulation Models: A New Research Agenda Through the SR vs ER Behavior Theory in Different Psychology Contexts. *Front. Psychol*. 13:861493. [doi:10.3389/fpsyg.2022.861493](https://doi.org/10.3389/fpsyg.2022.861493)

*De Lange AH, Van der Heijden B, Van Vuuren T, Furunes T, De Lange C and Dikkers J (2021) Employable as We Age? A Systematic Review of Relationships Between Age Conceptualizations and Employability. *Front. Psychol.* 11:605684. doi: [10.3389/fpsyg.2020.605684](https://doi.org/10.3389/fpsyg.2020.605684)

*De Oliveira JM, Dueñas J-M, Morales-Vives F and Gallardo-Nieto E (2023) Educational agents and institutions called into action in suicide prevention, intervention, and postvention. *Front. Psychol.* 14:1213751. [doi:10.3389/fpsyg.2023.1213751](https://doi.org/10.3389/fpsyg.2023.1213751)

*de Witte M, Kooijmans R, Hermanns M, van Hooren S, Biesmans K, Hermsen M, Stams GJ and Moonen X (2021) Self-Report Stress Measures to Assess Stress in Adults With Mild Intellectual Disabilities—A Scoping Review. *Front. Psychol.* 12:742566. doi: [10.3389/fpsyg.2021.742566](https://doi.org/10.3389/fpsyg.2021.742566)​

*de-la-Peña C and Luque-Rojas MJ (2021) Levels of Reading Comprehension in Higher Education: Systematic Review and Meta-Analysis. *Front. Psychol.* 12:712901. doi: [10.3389/fpsyg.2021.712901](https://doi.org/10.3389/fpsyg.2021.712901)​

*Delfa-Lobato L, Guàrdia-Olmos J and Feliu-Torruella M (2021) Benefits of Cultural Activities on People With Cognitive Impairment: A Systematic Review. *Front. Psychol.* 12:762392. doi: [10.3389/fpsyg.2021.762392](https://doi.org/10.3389/fpsyg.2021.762392)​

*Deng Q, Fu C, Ban M and Iio T (2024) A systematic review on robot-assisted language learning for adults. *Front. Psychol.* 15:1471370. [doi:10.3389/fpsyg.2024.1471370](https://doi.org/10.3389/fpsyg.2024.1471370)

*Deng X, Yang J and Wu Y (2021) Adolescent Empathy Influences Bystander Defending in School Bullying: A Three-Level Meta-Analysis. *Front. Psychol*. 12:690898. [doi:10.3389/fpsyg.2021.690898](https://doi.org/10.3389/fpsyg.2021.690898)

*Di H (2022) Educational Psychology-Empowered Creative Practice Strategy and Educational Countermeasures for Cinematography Major. *Front. Psychol.* 13:913294. doi: [10.3389/fpsyg.2022.913294](https://doi.org/10.3389/fpsyg.2022.913294)​

*Dias Rodrigues A, Cruz-Ferreira A, Marmeleira J and Veiga G (2022) Effects of Body-Oriented Interventions on Preschoolers’ Social-Emotional Competence: A Systematic Review. *Front. Psychol*. 12:752930. [doi:10.3389/fpsyg.2021.752930](https://doi.org/10.3389/fpsyg.2021.752930)

*Ding Y, Huang H, Zhang Y, Peng Q, Yu J, Lu G, Wu H and Chen C (2022) Correlations between smartphone addiction and alexithymia, attachment style, and subjective well-being: A meta-analysis. *Front. Psychol.* 13:971735. doi: [10.3389/fpsyg.2022.971735](https://doi.org/10.3389/fpsyg.2022.971735)​

*Ding Y, Wan X, Lu G, Huang H, Liang Y, Yu J and Chen C (2022) The associations between smartphone addiction and self-esteem, self-control, and social support among Chinese adolescents: A meta-analysis. *Front. Psychol.* 13:1029323. doi: [10.3389/fpsyg.2022.1029323](https://doi.org/10.3389/fpsyg.2022.1029323)​

*Doménech-Betoret F (2018) The Educational Situation Quality Model: Recent Advances. *Front. Psychol*. 9:328. [doi::10.3389/fpsyg.2018.00328](https://doi.org/10.3389/fpsyg.2018.00328)

*Domínguez-García E and Fernández-Berrocal P (2018) The Association Between Emotional Intelligence and Suicidal Behavior: A Systematic Review. *Front. Psychol.* 9:2380. doi: [10.3389/fpsyg.2018.02380](https://doi.org/10.3389/fpsyg.2018.02380)

*Dong H, Li W and Ye D (2022) The Influence of English as a Foreign Language Teachers’ Positive Mood and Hope on Their Academic Buoyancy: A Theoretical Review. *Front. Psychol.* 12:801435. doi: [10.3389/fpsyg.2021.801435](https://doi.org/10.3389/fpsyg.2021.801435)

*Dong Y and Zeb S (2022) Role of higher education system in promoting law abiding behavior among students. *Front. Psychol.* 13:1036991. [doi:10.3389/fpsyg.2022.1036991](https://doi.org/10.3389/fpsyg.2022.1036991)

*Dong Y, Peng S-N, Sun Y-K, Wu SX-Y and Wang W-S (2020) Reading Comprehension and Metalinguistic Knowledge in Chinese Readers: A Meta-Analysis. *Front. Psychol.* 10:3037. doi: [10.3389/fpsyg.2019.03037](https://doi.org/10.3389/fpsyg.2019.03037)

*Dreer B (2023) On the outcomes of teacher wellbeing: a systematic review of research. *Front. Psychol.* 14:1205179. doi: [10.3389/fpsyg.2023.1205179](https://doi.org/10.3389/fpsyg.2023.1205179)

*Dudley D, Mackenzie E, Van Bergen P, Cairney J and Barnett L (2022) What Drives Quality Physical Education? A Systematic Review and Meta-Analysis of Learning and Development Effects From Physical Education-Based Interventions. *Front. Psychol*. 13:799330. [doi:10.3389/fpsyg.2022.799330](https://doi.org/10.3389/fpsyg.2022.799330)

*Dumont E, Syurina EV, Feron FJM and van Hooren S (2017) Music Interventions and Child Development: A Critical Review and Further Directions. *Front. Psychol.* 8:1694. doi: [10.3389/fpsyg.2017.01694](https://doi.org/10.3389/fpsyg.2017.01694)

*Ebrahimi OV, Pallesen S, Kenter RMF and Nordgreen T (2019) Psychological Interventions for the Fear of Public Speaking: A Meta-Analysis. *Front. Psychol.* 10:488. doi: [10.3389/fpsyg.2019.00488](https://doi.org/10.3389/fpsyg.2019.00488)

*Edisherashvili N, Saks K, Pedaste M and Leijen Ä (2022) Supporting Self-Regulated Learning in Distance Learning Contexts at Higher Education Level: Systematic Literature Review. *Front. Psychol.* 12:792422. doi: [10.3389/fpsyg.2021.792422](https://doi.org/10.3389/fpsyg.2021.792422)

*Fàbregues S, Sáinz M, Romano MJ, Escalante-Barrios EL, Younas A and López-Pérez B-S (2023) Use of mixed methods research in intervention studies to increase young people’s interest in STEM: A systematic methodological review. *Front. Psychol*. 13:956300. [doi:10.3389/fpsyg.2022.956300](https://doi.org/10.3389/fpsyg.2022.956300)

*Fang J, Huang X, Zhang M, Huang F, Li Z and Yuan Q (2018) The Big-Fish-Little-Pond Effect on Academic Self-Concept: A Meta-Analysis. *Front. Psychol.* 9:1569. doi: [10.3389/fpsyg.2018.01569](https://doi.org/10.3389/fpsyg.2018.01569)

*Fang S and Ding D (2022) Which outcome variables are associated with psychological inflexibility/flexibility for chronic pain patients? A three level meta-analysis. *Front. Psychol.* 13:1069748. [doi:10.3389/fpsyg.2022.1069748](https://doi.org/10.3389/fpsyg.2022.1069748)

*Fernández-Martín F-D, Romero-Rodríguez J-M, Marín-Marín J-A and Gómez-García G (2021) Social and Emotional Learning in the Ibero-American Context: A Systematic Review. *Front. Psychol*. 12:738501. [doi:10.3389/fpsyg.2021.738501](https://doi.org/10.3389/fpsyg.2021.738501)

*Filipe MG, Cruz S, Veloso AS and Frota S (2022) Early predictors of language outcomes in Down syndrome: A mini-review. *Front. Psychol.* 13:934490. doi: [10.3389/fpsyg.2022.934490](https://doi.org/10.3389/fpsyg.2022.934490)

*Filipe MG, Magalhães S, Veloso AS, Costa AF, Ribeiro L, Araújo P, Castro SL and Limpo T (2021) Exploring the effects of meditation techniques used by mindfulness-based programs on the cognitive, social-emotional, and academic skills of children: A systematic review. *Front. Psychol.* 12:660650. doi: [10.3389/fpsyg.2021.660650](https://doi.org/10.3389/fpsyg.2021.660650)

*Finell J, Sammallahti E, Korhonen J, Eklöf H and Jonsson B (2022) Working memory and its mediating role on the relationship of math anxiety and math performance: A meta-analysis. *Front. Psychol.* 12:798090. doi: [10.3389/fpsyg.2021.798090](https://doi.org/10.3389/fpsyg.2021.798090)​

*François C, Grau-Sánchez J, Duarte E and Rodriguez-Fornells A (2015) Musical training as an alternative and effective method for neuro-education and neuro-rehabilitation. *Front. Psychol.* 6:475. doi: [10.3389/fpsyg.2015.00475](https://doi.org/10.3389/fpsyg.2015.00475)

*Frantz J, Cupido-Masters J, Moosajee F and Smith MR (2022) Non-cognitive support for postgraduate studies: A systematic review. *Front. Psychol.* 12:773910. doi: [10.3389/fpsyg.2021.773910](https://doi.org/10.3389/fpsyg.2021.773910)

*Fu F, Zhao H, Tong F and Chi I (2017) A systematic review of psychosocial interventions to cancer caregivers. *Front. Psychol.* 8:834. doi: [10.3389/fpsyg.2017.00834](https://doi.org/10.3389/fpsyg.2017.00834)

*Fulantelli G, Taibi D, Scifo L, Schwarze V and Eimler SC (2022) Cyberbullying and Cyberhate as Two Interlinked Instances of Cyber-Aggression in Adolescence: A Systematic Review. *Front. Psychol*. 13:909299. [doi:10.3389/fpsyg.2022.909299](https://doi.org/10.3389/fpsyg.2022.909299)

*Fuller B, Bajaba A and Bajaba S (2022) Enhancing and extending the meta-analytic comparison of newer genre leadership forms. *Front. Psychol.* 13:872568. doi: [10.3389/fpsyg.2022.872568](https://doi.org/10.3389/fpsyg.2022.872568)

*Gao Y (2021) Toward the role of language teacher confirmation and stroke in EFL/ESL students' motivation and academic engagement: A theoretical review. *Front. Psychol.* 12:723432. doi: [10.3389/fpsyg.2021.723432](https://doi.org/10.3389/fpsyg.2021.723432)

*Gao Y, Soh KG, Zakaria NS, Mohd Rasdi R, Guo WandSohKL (2023) The effect of knowledge transfer theory on the selection of crossover winter sports athletes: A systematic literature review. *Front. Psychol*. 13:1001082. [doi:10.3389/fpsyg.2022.1001082](https://doi.org/10.3389/fpsyg.2022.1001082)

*Gao Z, Chee CS, Norjali Wazir MRW, Wang J, Zheng X and Wang T (2024) The role of parents in the motivation of young athletes: a systematic review. *Front. Psychol.* 14:1291711. [doi:10.3389/fpsyg.2023.1291711](https://doi:10.3389/fpsyg.2023.1291711)

*García-Carrión R, López de Aguileta G, Padrós M and Ramis-Salas M (2020) Implications for social impact of dialogic teaching and learning. *Front. Psychol.* 11:140. doi: [10.3389/fpsyg.2020.00140](https://doi.org/10.3389/fpsyg.2020.00140)

*Gegenfurtner A (2022) Bifactor exploratory structural equation modeling: A meta-analytic review of model fit. *Front. Psychol.* 13:1037111. doi: [10.3389/fpsyg.2022.1037111](https://doi.org/10.3389/fpsyg.2022.1037111)​

*Goetz CD, Pillsworth EG, Buss DM and Conroy-Beam D (2019) Evolutionary Mismatch in Mating. *Front. Psychol.* 10:2709. doi: [10.3389/fpsyg.2019.02709](https://doi.org/10.3389/fpsyg.2019.02709)

*Gómez-Zúñiga B, Pousada M and Armayones M (2023) Loneliness and disability: A systematic review of loneliness conceptualization and intervention strategies. *Front. Psychol.* 13:1040651. doi: [10.3389/fpsyg.2022.1040651](https://doi.org/10.3389/fpsyg.2022.1040651)

*Gong X-G, Wang L-P, Rong G, Zhang D-N, Zhang A-Y and Liu C (2023) Effects of online mindfulness-based interventions on the mental health of university students: A systematic review and meta-analysis. *Front. Psychol.* 14:1073647. doi: [10.3389/fpsyg.2023.1073647](https://doi.org/10.3389/fpsyg.2023.1073647)

*Grebosz-Haring K, Thun-Hohenstein L, Schuchter-Wiegand AK, Irons Y, Bathke A, Phillips K and Clift S (2022) The Need for Robust Critique of Arts and Health Research: Young People, Art Therapy and Mental Health. *Front. Psychol.* 13:821093. doi: [10.3389/fpsyg.2022.821093](https://doi.org/10.3389/fpsyg.2022.821093)

*Greving S and Richter T (2018) Examining the Testing Effect in University Teaching: Retrievability and Question Format Matter. *Front. Psychol.* 9:2412. doi: [10.3389/fpsyg.2018.02412](https://doi.org/10.3389/fpsyg.2018.02412)

*Grolig L (2020) Shared Storybook Reading and Oral Language Development: A Bioecological Perspective. *Front. Psychol*. 11:1818. [doi:10.3389/fpsyg.2020.01818](https://doi.org/10.3389/fpsyg.2020.01818)

*Gumz A, Treese B, Marx C, Strauss B and Wendt H (2015) Measuring Verbal Psychotherapeutic Techniques—A Systematic Review of Intervention Characteristics and Measures. *Front. Psychol.* 6:1705. doi: [10.3389/fpsyg.2015.01705](https://doi.org/10.3389/fpsyg.2015.01705)

*Gutiérrez-Ángel N, Sánchez-García J-N, Mercader-Rubio I, García-Martín J and Brito-Costa S (2022) Digital literacy in the university setting: A literature review of empirical studies between 2010 and 2021. *Front. Psychol.* 13:896800. [doi:10.3389/fpsyg.2022.896800](https://doi.org/10.3389/fpsyg.2022.896800)

*Guzzardi M, Menghini D, Costanzo F, Vicari S and Foti F (2024) Prospective memory in the developmental age: a systematic review to synthesize the evaluation tools and the main cognitive functions involved. *Front. Psychol.* 15:1394586. [doi:10.3389/fpsyg.2024.1394586](https://doi.org/10.3389/fpsyg.2024.1394586)

*Haberstroh S and Schulte-Körne G (2022) The Cognitive Profile of Math Difficulties: A Meta-Analysis Based on Clinical Criteria. *Front. Psychol.* 13:842391. doi: [10.3389/fpsyg.2022.842391](https://doi.org/10.3389/fpsyg.2022.842391)​

*Hammerstein S, König C, Dreisörner T and Frey A (2021) Effects of COVID-19-Related School Closures on Student Achievement—A Systematic Review. *Front. Psychol.* 12:746289. doi: [10.3389/fpsyg.2021.746289](https://doi.org/10.3389/fpsyg.2021.746289)​

*Hancock PA, Kessler TT, Kaplan AD, Stowers K, Brill JC, Billings DR, Schaefer KE and Szalma JL (2023) How and why humans trust: A meta-analysis and elaborated model. *Front. Psychol.* 14:1081086. doi: [10.3389/fpsyg.2023.1081086](https://doi.org/10.3389/fpsyg.2023.1081086)

*Hanson JL, Rosenberg AA and Lane JL (2013) Narrative descriptions should replace grades and numerical ratings for clinical performance in medical education in the United States. *Front. Psychol*. **4**:668. [doi:10.3389/fpsyg.2013.00668](https://doi.org/10.3389/fpsyg.2013.00668)

*Hantman RM, Choi B, Hartwick K, Nadler Z and Luk G (2023) A systematic review of bilingual experiences, labels, and descriptions in autism spectrum disorder research. *Front. Psychol.* 14:1095164. doi: [10.3389/fpsyg.2023.1095164](https://doi.org/10.3389/fpsyg.2023.1095164)

*He J, Yu H, Jiang M and Bialas M (2023) A research synthesis on successful educational practices and student outcomes for physical education in schools. *Front. Psychol.* 14:1280871. [doi:10.3389/fpsyg.2023.1280871](https://doi.org/10.3389/fpsyg.2023.1280871)

*He J-L, Xu H-Q, Yang J, Hou D-J, Gong X-Y, Lu X-Y, Wang W, Cai M-J, Yu Y-F and Gao J (2023) Fear of disease progression among breast cancer patients in China: a meta-analysis of studies using the fear of progression questionnaire short form. *Front. Psychol*. 14:1222798. [doi:10.3389/fpsyg.2023.1222798](https://doi.org/10.3389/fpsyg.2023.1222798)

*Hendriks F, Mayweg-Paus E, Felton M, Iordanou K, Jucks R and Zimmermann M (2020) Constraints and Affordances of Online Engagement With Scientific Information—A Literature Review. *Front. Psychol*. 11:572744. [doi:10.3389/fpsyg.2020.572744](https://doi.org/10.3389/fpsyg.2020.572744)

*Heyne D, Strömbeck J, Alanko K, Bergström M and Ulriksen R (2020) A scoping review of constructs measured following intervention for school refusal: Are we measuring up? *Front. Psychol.* 11:1744. doi: [10.3389/fpsyg.2020.01744](https://doi.org/10.3389/fpsyg.2020.01744)

*Hmelo-Silver CE and Jeong H (2021) Benefits and challenges of interdisciplinarity in CSCL research: A view from the literature. *Front. Psychol.* 11:579986. doi: [10.3389/fpsyg.2020.579986](https://doi.org/10.3389/fpsyg.2020.579986)

*Hobbs C, Armitage J, Hood B and Jelbert S (2022) A systematic review of the effect of university positive psychology courses on student psychological wellbeing. *Front. Psychol.* 13:1023140. doi: [10.3389/fpsyg.2022.1023140](https://doi.org/10.3389/fpsyg.2022.1023140)

*Hoedemakers J, Vanderstukken A and Stoffers J (2023) The influence of leadership on employees' employability: a bibliometric analysis, systematic literature review, and research agenda. *Front. Psychol.* 14:1092865. [doi:10.3389/fpsyg.2023.1092865](https://doi.org/10.3389/fpsyg.2023.1092865)

*Hokstad S and Næss K-AB (2023) Stuttering in individuals with Down syndrome: a systematic review of earlier research. *Front. Psychol.* 14:1176743. doi: [10.3389/fpsyg.2023.1176743](https://doi.org/10.3389/fpsyg.2023.1176743)​

*Horner SL and Shaffer SA (2021) Evaluating the Reporting Quality of Researcher-Developed Alphabet Knowledge Measures: How Transparent and Replicable Is It? *Front. Psychol*. 12:601849. [doi:10.3389/fpsyg.2021.601849](https://doi.org/10.3389/fpsyg.2021.601849)

*Horvath JC and Donoghue GM (2016) A Bridge Too Far– Revisited: Reframing Bruer’s Neuroeducation Argument for Modern Science of Learning Practitioners. *Front. Psychol*. 7:377. [doi:10.3389/fpsyg.2016.00377](http://dx.doi.org/10.3389/fpsyg.2016.00377)

*Hsieh C-C and Li S (2024) A bibliometrics review of the journal mindfulness: science mapping the literature from 2012 to 2022. *Front. Psychol.* 15:1378143. [doi:10.3389/fpsyg.2024.1378143](https://doi.org/10.3389/fpsyg.2024.1378143)

*Hu J and Cheung CKJ (2024) Social identity and social integration: a meta-analysis exploring the relationship between social identity and social integration. *Front. Psychol.* 15:1361163. doi: [10.3389/fpsyg.2024.1361163](https://doi.org/10.3389/fpsyg.2024.1361163)​

*Hu T, Wang X and Xu H (2022) Eye-tracking in interpreting studies: A review of four decades of empirical studies. *Front. Psychol.* 13:872247. doi: [10.3389/fpsyg.2022.872247](https://doi.org/10.3389/fpsyg.2022.872247)​

*Hua J, Zheng K and Fan S (2022) The impact of entrepreneurial activities and college students’ entrepreneurial abilities in higher education—A meta-analytic path. *Front. Psychol.* 13:843978. doi: [10.3389/fpsyg.2022.843978](https://doi.org/10.3389/fpsyg.2022.843978)​

*Huang C (2022) Self-regulation of learning and EFL learners’ hope and joy: A review of literature. *Front. Psychol.* 13:833279. doi: [10.3389/fpsyg.2022.833279](https://doi.org/10.3389/fpsyg.2022.833279)

*Huang W, Song F, Zhang S and Xia T (2022) Influence of deep learning-based journal reading guidance system on students’ national cognition and cultural acceptance. *Front. Psychol.* 13:950412. doi: [10.3389/fpsyg.2022.950412](https://doi.org/10.3389/fpsyg.2022.950412)

*Jebb AT, Ng V and Tay L (2021) A review of key Likert scale development advances: 1995–2019. *Front. Psychol.* 12:637547. doi: [10.3389/fpsyg.2021.637547](https://doi.org/10.3389/fpsyg.2021.637547)

*Ji S, Qin X and LI K (2022) A systematic review of foreign language listening anxiety: focus on the theoretical definitions and measurements. *Front. Psychol.* 13:859021. doi: [10.3389/fpsyg.2022.859021](https://doi.org/10.3389/fpsyg.2022.859021)

*Jia H (2022) English as a foreign language learners’ well-being and their academic engagement: the mediating role of English as a foreign language learners’ self-efficacy. *Front. Psychol.* 13:882886. doi: [10.3389/fpsyg.2022.882886](https://doi.org/10.3389/fpsyg.2022.882886)​

*Jiang Q, Shi L, Zheng D and Mao W (2023) Parental homework involvement and students’ mathematics achievement: a meta-analysis. *Front. Psychol.* 14:1218534. doi: [10.3389/fpsyg.2023.1218534](https://doi.org/10.3389/fpsyg.2023.1218534)​

*Jianping G, Zhihui Z, Roslan S, Zaremohzzabieh Z, Burhanuddin NAN and Geok SK (2023) Improving hardiness among university students: a meta-analysis of intervention studies. *Front. Psychol.* 13:994453. doi: [10.3389/fpsyg.2022.994453](https://doi.org/10.3389/fpsyg.2022.994453)

*Jinmin Z and Qi F (2023) Relationship between learning flow and academic performance among students: a systematic evaluation and meta-analysis. *Front. Psychol.* 14:1270642. doi: [10.3389/fpsyg.2023.1270642](https://doi.org/10.3389/fpsyg.2023.1270642)

*Jolles J and Jolles DD (2021) On Neuroeducation: Why and How to Improve Neuroscientific Literacy in Educational Professionals. *Front. Psychol.* 12:752151. [doi:10.3389/fpsyg.2021.752151](https://doi.org/10.3389/fpsyg.2021.752151)

*Juhani Lyytinen H, Semrud-Clikeman M, Li H, Pugh K and Richardson U (2021) Supporting Acquisition of Spelling Skills in Different Orthographies Using an Empirically Validated Digital Learning Environment. *Front. Psychol*. 12:566220. [doi:10.3389/fpsyg.2021.566220](https://doi.org/10.3389/fpsyg.2021.566220)

*Kenny SA, Cameron CE, Karing JT, Ahmadi A, Braithwaite PN and McClelland MM (2023) A meta-analysis of the validity of the Head-Toes-Knees-Shoulders task in predicting young children’s academic performance. *Front. Psychol.* 14:1124235. doi: [10.3389/fpsyg.2023.1124235](https://doi.org/10.3389/fpsyg.2023.1124235)

*Kievit RA, Frankenhuis WE, Waldorp LJ and Borsboom D (2013) Simpson's paradox in psychological science: a practical guide. *Front. Psychol*. **4**:513. doi: [10.3389/fpsyg.2013.00513](https://doi.org/10.3389/fpsyg.2013.00513)

*Kim H-D and Cruz AB (2021) Psychological Influence of Self-Management on Exercise Self-Confidence, Satisfaction, and Commitment of Martial Arts Practitioners in Korea: A Meta-Analytic Approach. *Front. Psychol*. 12:691974. [doi:10.3389/fpsyg.2021.691974](https://doi.org/10.3389/fpsyg.2021.691974)

*Kim S-i (2013) Neuroscientific model of motivational process. *Front. Psychol.* **4**:98. [doi:10.3389/fpsyg.2013.00098](https://doi.org/10.3389/fpsyg.2013.00098)

*Knitter LA, Hoffmann J, Eid M and Koch T (2025) Measuring the dark triad: a meta-analytical SEM study of two prominent short scales*. Front. Psychol.* 15:1469970. [doi:10.3389/fpsyg.2024.1469970](https://doi.org/10.3389/fpsyg.2024.1469970)

*Knogler M, Hetmanek A and Seidel T (2022) Determining an evidence base for particular fields of educational practice: a systematic review of meta-analyses on effective mathematics and science teaching. *Front. Psychol.* 13:873995. doi: [10.3389/fpsyg.2022.873995](https://doi.org/10.3389/fpsyg.2022.873995)​

*Kobal Grum D and Babnik K (2022) The psychological concept of social sustainability in the workplace from the perspective of sustainable goals: A systematic review. *Front. Psychol.* 13:942204. doi: [10.3389/fpsyg.2022.942204](https://doi.org/10.3389/fpsyg.2022.942204)

*Kominsky JF, Bascandziev I, Shafto P and Bonawitz E (2023) Talk of the Town mobile app platform: New method for engaging family in STEM learning and research in homes and communities. *Front. Psychol.* 14:1110940. doi: [10.3389/fpsyg.2023.1110940](https://doi.org/10.3389/fpsyg.2023.1110940)

*Kong Y (2021) The Role of Experiential Learning on Students’ Motivation and Classroom Engagement. *Front. Psychol.* 12:771272. doi: [10.3389/fpsyg.2021.771272](https://doi.org/10.3389/fpsyg.2021.771272)

*Kopp SL, Ramseier CA, Ratka-Krüger P and Woelber JP (2017) Motivational Interviewing As an Adjunct to Periodontal Therapy—A Systematic Review. *Front. Psychol.* 8:279. doi: [10.3389/fpsyg.2017.00279](https://doi.org/10.3389/fpsyg.2017.00279)

*Kotera Y and Van Gordon W (2021) Effects of Self-Compassion Training on Work-Related Well-Being: A Systematic Review. *Front. Psychol.* 12:630798. doi: [10.3389/fpsyg.2021.630798](https://doi.org/10.3389/fpsyg.2021.630798)​

*Koutsimani P, Montgomery A and Georganta K (2019) The Relationship Between Burnout, Depression, and Anxiety: A Systematic Review and Meta-Analysis. *Front. Psychol.* 10:284. doi: [10.3389/fpsyg.2019.00284](https://doi.org/10.3389/fpsyg.2019.00284)

*Krägeloh CU, Bharatharaj J, Albo-Canals J, Hannon D and Heerink M (2022) The time is ripe for robopsychology. *Front. Psychol.* 13:968382. doi: [10.3389/fpsyg.2022.968382](https://doi.org/10.3389/fpsyg.2022.968382)

*Kritikou M and Giovazolias T (2022) Emotion regulation, academic buoyancy, and academic adjustment of university students within a self-determination theory framework: A systematic review. *Front. Psychol.* 13:1057697. doi: [10.3389/fpsyg.2022.1057697](https://doi.org/10.3389/fpsyg.2022.1057697)

*Larrain A, Fortes G and Rojas MT (2021) Deliberative Teaching as an Emergent Field: The Challenge of Articulating Diverse Research Agendas to Promote Educational Experiences for Citizenship. *Front. Psychol*. 12:660825. [doi:10.3389/fpsyg.2021.660825](https://doi.org/10.3389/fpsyg.2021.660825)

*Lauder K, McDowall A and Tenenbaum HR (2022) A systematic review of interventions to support adults with ADHD at work—Implications from the paucity of context-specific research for theory and practice. *Front. Psychol.* 13:893469. doi: [10.3389/fpsyg.2022.893469](https://doi.org/10.3389/fpsyg.2022.893469)

*Lea RG, Davis SK, Mahoney B and Qualter P (2019) Does Emotional Intelligence Buffer the Effects of Acute Stress? A Systematic Review. *Front. Psychol*. 10:810. [doi:10.3389/fpsyg.2019.00810](https://doi.org/10.3389/fpsyg.2019.00810)

*Léger-Goodes T, Malboeuf-Hurtubise C, Mastine T, Généreux M, Paradis P-O and Camden C (2022) Eco-anxiety in children: A scoping review of the mental health impacts of the awareness of climate change. *Front. Psychol.* 13:872544. doi: [10.3389/fpsyg.2022.872544](https://doi.org/10.3389/fpsyg.2022.872544)

*Lembke EJ, Linderkamp F and Casale G (2024) Trauma-sensitive school concepts for students with a refugee background: a review of international studies. *Front. Psychol*. 15:1321373. [doi:10.3389/fpsyg.2024.1321373](https://doi.org/10.3389/fpsyg.2024.1321373)

*Lentz LM, Smith-MacDonald L, Malloy D, Carleton RN and Brémault-Phillips S (2021) Compromised Conscience: A Scoping Review of Moral Injury Among Firefighters, Paramedics, and Police Officers. *Front. Psychol.* 12:639781. doi: [10.3389/fpsyg.2021.639781](https://doi.org/10.3389/fpsyg.2021.639781)​

*Leppänen M, Korja R, Rautava P and Ahlqvist-Björkroth S (2024) Early psychosocial parent–infant interventions and parent–infant relationships after preterm birth–a scoping review. *Front. Psychol.* 15:1380826. [doi:10.3389/fpsyg.2024.1380826](https://doi.org/10.3389/fpsyg.2024.1380826)

*Li B (2022) Boosting EFL learners’ commitment and enjoyment in language learning through social networking: A literature review. *Front. Psychol.* 13:999586. doi: [10.3389/fpsyg.2022.999586](https://doi.org/10.3389/fpsyg.2022.999586)

*Li F, Liu J, Qiu F, Liu H, Xin S and Yang Q (2022) Changes in mental health levels among Chinese physical education college students from 1995 to 2019. *Front. Psychol.* 13:1034221. doi: [10.3389/fpsyg.2022.1034221](https://doi.org/10.3389/fpsyg.2022.1034221)

*Li J and Jiang Y (2021) The Research Trend of Big Data in Education and the Impact of Teacher Psychology on Educational Development During COVID-19: A Systematic Review and Future Perspective. *Front. Psychol.* 12:753388. doi: [10.3389/fpsyg.2021.753388](https://doi.org/10.3389/fpsyg.2021.753388)​

*Li J, Ye H, Tang Y, Zhou Z and Hu X (2018) What Are the Effects of Self-Regulation Phases and Strategies for Chinese Students? A Meta-Analysis of Two Decades Research of the Association Between Self-Regulation and Academic Performance. *Front. Psychol.* 9:2434. doi: [10.3389/fpsyg.2018.02434](https://doi.org/10.3389/fpsyg.2018.02434)

*Li M (2022) On the Role of Psychological Health and Buoyancy in EFL Teachers' Professional Commitment. *Front. Psychol.* 13:897488. doi: [10.3389/fpsyg.2022.897488](https://doi.org/10.3389/fpsyg.2022.897488)​

*Li M and Yu Z (2023) A systematic review on the metaverse-based blended English learning. *Front. Psychol.* 13:1087508. [doi:10.3389/fpsyg.2022.1087508](https://doi.org/10.3389/fpsyg.2022.1087508)

*Li M, Ma S and Shi Y (2023) Examining the effectiveness of gamification as a tool promoting teaching and learning in educational settings: a meta-analysis. *Front. Psychol.* 14:1253549. doi: [10.3389/fpsyg.2023.1253549](https://doi.org/10.3389/fpsyg.2023.1253549)

*Li W (2022) Resilience Among Language Learners: The Roles of Support, Self-Efficacy, and Buoyancy. *Front. Psychol.* 13:854522. doi: [10.3389/fpsyg.2022.854522](https://doi.org/10.3389/fpsyg.2022.854522)​

*Li Z, Slavkova O and Gao Y (2022) Role of digitalization, digital competence, and parental support on performance of sports education in low-income college students. *Front. Psychol.* 13:979318. [doi:10.3389/fpsyg.2022.979318](https://doi.org/10.3389/fpsyg.2022.979318)

*Li, J.; Ye, H.; Tang, Y. & Hu, X. (2018). What are the effects of self-regulation phases and strategies for Chinese students? A meta-analysis of two decades research of the association betweenself-regulationand academicPerformance. *Frontiers in Psychology* [doi:10.3389/fpsyg.2018.02434](https://doi.org/10.3389/fpsyg.2018.02434)

*Limone P and Toto GA (2022) Psychological and Emotional Effects of Digital Technology on Digitods (14–18 Years): A Systematic Review. *Front. Psychol.* 13:938965. doi: [10.3389/fpsyg.2022.938965](https://doi.org/10.3389/fpsyg.2022.938965)

*Lin X, Li R, Chen Z and Xiong J (2024) Design strategies for VR science and education games from an embodied cognition perspective: a literature-based meta-analysis. *Front. Psychol.* 14:1292110. doi: [10.3389/fpsyg.2023.1292110](https://doi.org/10.3389/fpsyg.2023.1292110)

*Lisboa PV, Gómez-Román C, Guntín L and Monteiro AP (2024) Pro-environmental behavior, personality and emotional intelligence in adolescents: a systematic review. *Front. Psychol.* 15:1323098. doi: [10.3389/fpsyg.2024.1323098](https://doi.org/10.3389/fpsyg.2024.1323098)

*Liu C and Rahman MNA (2022) Relationships between parenting style and sibling conflicts: A meta-analysis. *Front. Psychol.* 13:936253. doi: [10.3389/fpsyg.2022.936253](https://doi.org/10.3389/fpsyg.2022.936253)​

*Liu H, Soh KG, Samsudin S, Rattanakoses W and Qi F (2022) Effects of exercise and psychological interventions on smartphone addiction among university students: A systematic review. *Front. Psychol.* 13:1021285. [doi:10.3389/fpsyg.2022.1021285](https://doi.org/10.3389/fpsyg.2022.1021285)

*Liu W (2021) Does Teacher Immediacy Affect Students? A Systematic Review of the Association Between Teacher Verbal and Non-verbal Immediacy and Student Motivation. *Front. Psychol.* 12:713978. doi: [10.3389/fpsyg.2021.713978](https://doi.org/10.3389/fpsyg.2021.713978)

*Liu Y, Zhang M, Zhao X and Jia F (2021) Fostering EFL/ESL Students’ Language Achievement: The Role of Teachers’ Enthusiasm and Classroom Enjoyment. *Front. Psychol.* 12:781118. doi: [10.3389/fpsyg.2021.781118](https://doi.org/10.3389/fpsyg.2021.781118)

*Liu Z (2022) The Interplay of English as a Foreign Language Learners' Interest, Self-Efficacy, and Involvement. *Front. Psychol.* 13:837286. doi: [10.3389/fpsyg.2022.837286](https://doi.org/10.3389/fpsyg.2022.837286)

*Llistosella M, Goni-Fuste B, Martín-Delgado L, Miranda-Mendizabal A, Franch Martinez B, Pérez-Ventana C and Castellvi P (2023) Effectiveness of resilience-based interventions in schools for adolescents: a systematic review and meta-analysis. *Front. Psychol.* 14:1211113. doi: [10.3389/fpsyg.2023.1211113](https://doi.org/10.3389/fpsyg.2023.1211113)

*Lo CK and Hew KF (2021) Student Engagement in Mathematics Flipped Classrooms: Implications of Journal Publications From 2011 to 2020. *Front. Psychol.* 12:672610. doi: [10.3389/fpsyg.2021.672610](https://doi.org/10.3389/fpsyg.2021.672610)

*Locher FM and Philipp M (2023) Measuring reading behavior in large-scale assessments and surveys. *Front. Psychol.* 13:1044290. [doi:10.3389/fpsyg.2022.1044290](https://doi.org/10.3389/fpsyg.2022.1044290)

*Lopes S, Magalhães P, Pereira A, Martins J, Magalhães C, Chaleta E and Rosário P (2018) Games Used With Serious Purposes: A Systematic Review of Interventions in Patients With Cerebral Palsy. *Front. Psychol.* 9:1712. doi: [10.3389/fpsyg.2018.01712](https://doi.org/10.3389/fpsyg.2018.01712)​

*López-Escribano C, Martín-Babarro J and Pérez-López R (2022) Promoting Handwriting Fluency for Preschool and Elementary-Age Students: Meta-Analysis and Meta-Synthesis of Research From 2000 to 2020. *Front. Psychol.* 13:841573. doi: [10.3389/fpsyg.2022.841573](https://doi.org/10.3389/fpsyg.2022.841573)

*López-Íñiguez G and McPherson GE (2023) Caring approaches to young, gifted music learners’ education: a PRISMA scoping review. *Front. Psychol.* 14:1167292. doi: [10.3389/fpsyg.2023.1167292](https://doi.org/10.3389/fpsyg.2023.1167292)

*Loyola-Carrillo P, Vega-Muñoz A, Salazar-Sepúlveda G, Gil-Marín M and Adsuar-Sala J (2025) Studying engagement in educational settings: a mapping review on high-impact academic engagement research. *Front. Psychol*. 16:1519509. [doi:10.3389/fpsyg.2025.1519509](https://doi.org/10.3389/fpsyg.2025.1519509)

*Luan H, Geczy P, Lai H, Gobert J, Yang SJH, Ogata H, Baltes J, Guerra R, Li P and Tsai C-C (2020) Challenges and Future Directions of Big Data and Artificial Intelligence in Education. *Front. Psychol.* 11:580820. [doi:10.3389/fpsyg.2020.580820](https://doi.org/10.3389/fpsyg.2020.580820)

*Ma Q and Wang F (2022) The Role of Students’ Spiritual Intelligence in Enhancing Their Academic Engagement: A Theoretical Review. *Front. Psychol.* 13:857842. doi: [10.3389/fpsyg.2022.857842](https://doi.org/10.3389/fpsyg.2022.857842)​

*Ma Y (2022) On the Relationship Between English as a Foreign Language Learners’ Positive Affectivity, Academic Disengagement, and Communication Apprehension. *Front. Psychol.* 12:828873. doi: [10.3389/fpsyg.2021.828873](https://doi.org/10.3389/fpsyg.2021.828873)

*Ma Y (2022) The triarchy of L2 learners’ emotion, cognition, and language performance: Anxiety, self-efficacy, and speaking skill in lights of the emerging theories in SLA. *Front. Psychol.* 13:1002492. doi: [10.3389/fpsyg.2022.1002492](https://doi.org/10.3389/fpsyg.2022.1002492)

*Macedonia M (2019) Embodied Learning: Why at School the Mind Needs the Body. *Front. Psychol*. 10:2098. [doi:10.3389/fpsyg.2019.02098](https://doi.org/10.3389/fpsyg.2019.02098)

*Madni SHH, Ali J, Husnain HA, Masum MH, Mustafa S, Shuja J, Maray M and Hosseini S (2022) Factors Influencing the Adoption of IoT for E-Learning in Higher Educational Institutes in Developing Countries. *Front. Psychol*. 13:915596. [doi:10.3389/fpsyg.2022.915596](https://doi.org/10.3389/fpsyg.2022.915596)

*Maier NA, Mendzheritskaya J, Hagenauer G, Hansen M, Kordts R, Stephan M and Thies K (2023) Developing a CVTAE-based conceptual framework for examining emotions in higher education teaching: a systematic literature review. *Front. Psychol.* 14:1142506. doi: [10.3389/fpsyg.2023.1142506](https://doi.org/10.3389/fpsyg.2023.1142506)

*Maldonado Briegas JJ, Sánchez Iglesias AI, Ballester SG and Vicente Castro F (2020) The Well-Being of the Elderly: Memory and Aging. *Front. Psychol.* 11:778. [doi:10.3389/fpsyg.2020.00778](https://doi.org/10.3389/fpsyg.2020.00778)

*Manjarres MT, Duarte DPM, Navarro-Obeid J, Álvarez MLV, Martinez I, Cudris-Torres L, Hernández-Lalinde J and Bermúdez V (2023) A bibliometric analysis and literature review on emotional skills. *Front. Psychol.* 14:1040110. doi: [10.3389/fpsyg.2023.1040110](https://doi.org/10.3389/fpsyg.2023.1040110)

*Mao F, Huang F, Zhao S and Fang Q (2024) Effects of cognitively engaging physical activity interventions on executive function in children and adolescents: a systematic review and meta-analysis. *Front. Psychol*. 15:1454447. [doi:10.3389/fpsyg.2024.1454447](https://doi.org/10.3389/fpsyg.2024.1454447)

*Mao F, Yin A, Zhao S and Fang Q (2024) Effects of football training on cognitive performance in children and adolescents: a meta-analytic review*. Front. Psychol*. 15:1449612. [doi:/10.3389/fpsyg.2024.1449612](https://doi.org/10.3389/fpsyg.2024.1449612)

*Mao P, Cai Z, He J, Chen X and Fan X (2021) The Relationship Between Attitude Toward Science and Academic Achievement in Science: A Three-Level Meta-Analysis. *Front. Psychol.* 12:784068. doi: [10.3389/fpsyg.2021.784068](https://doi.org/10.3389/fpsyg.2021.784068)

*Marino L and Capone V (2024) Psychosocial factors contributing to value creation in value-based healthcare: a scoping review. *Front. Psychol.* 15:1323110. doi: [10.3389/fpsyg.2024.1323110](https://doi.org/10.3389/fpsyg.2024.1323110)

*Martín-Martín M and Bueno-Álvarez JA (2023) Social networks and aggressive attitudes: who is who. Scoping review of the scientific production on their relationships. *Front. Psychol.* 14:1249907. doi: [10.3389/fpsyg.2023.1249907](https://doi.org/10.3389/fpsyg.2023.1249907)

*Mavilidi MF, Ruiter M, Schmidt M, Okely AD, Loyens S, Chandler P and Paas F (2018) A Narrative Review of School-Based Physical Activity for Enhancing Cognition and Learning: The Importance of Relevancy and Integration. *Front. Psychol*. 9:2079. [doi:10.3389/fpsyg.2018.02079](https://doi.org/10.3389/fpsyg.2018.02079)

*McLean CP, Utpala R and Sharp G (2022) The impacts of COVID-19 on eating disorders and disordered eating: A mixed studies systematic review and implications. *Front. Psychol.* 13:926709. doi: [10.3389/fpsyg.2022.926709](https://doi.org/10.3389/fpsyg.2022.926709)

*Meixner J and Kotrschal K (2022) Animal-Assisted Interventions With Dogs in Special Education—A Systematic Review. *Front. Psychol*. 13:876290. [doi:10.3389/fpsyg.2022.876290](https://doi.org/10.3389/fpsyg.2022.876290)

*Mendonça AR, Loureiro LM, Nórte CE and Landeira-Fernandez J (2022) Episodic memory training in elderly: A systematic review. *Front. Psychol.* 13:947519. doi: [10.3389/fpsyg.2022.947519](https://doi.org/10.3389/fpsyg.2022.947519)

*Menezes IG, Duran VR, Mendonça Filho EJ, Veloso TJ, Sarmento SMS, Paget CL and Ruggeri K (2016) Policy implications of achievement testing using multilevel models: The case of Brazilian elementary schools. *Front. Psychol.* 7:1727. doi: [10.3389/fpsyg.2016.01727](https://doi.org/10.3389/fpsyg.2016.01727)

*Metzner F, Adedeji A, Wichmann ML-Y, Zaheer Z, Schneider L, Schlachzig L, Richters J, Heumann S and Mays D (2022) Experiences of discrimination and everyday racism among children and adolescents with an immigrant background – Results of a systematic literature review on the impact of discrimination on the developmental outcomes of minors worldwide. *Front. Psychol.* 13:805941. doi: [10.3389/fpsyg.2022.805941](https://doi.org/10.3389/fpsyg.2022.805941)

*Meyerhofer-Parra R and González-Martínez J (2023) Transmedia storytelling usage of neural networks from a Universal Design for Learning perspective: A systematic review. *Front. Psychol.* 14:1119551. [doi:10.3389/fpsyg.2023.1119551](https://doi.org/10.3389/fpsyg.2023.1119551)

*Midgley N, Mortimer R, Cirasola A, Batra P and Kennedy E (2021) The Evidence-Base for Psychodynamic Psychotherapy With Children and Adolescents: A Narrative Synthesis. *Front. Psychol*. 12:662671. [doi:10.3389/fpsyg.2021.662671](https://doi.org/10.3389/fpsyg.2021.662671)

*Mitic M, Woodcock KA, Amering M, Krammer I, Stiehl KAM, Zehetmayer S and Schrank B (2021) Toward an integrated model of supportive peer relationships in early adolescence: A systematic review and exploratory meta-analysis. *Front. Psychol.* 12:589403. doi: [10.3389/fpsyg.2021.589403](https://doi.org/10.3389/fpsyg.2021.589403)

*Molina I, Molina-Perez E, Sobrino F, Tellez-Rojas MA, Zamora-Maldonado HC, Plaza-Ferreira M, Orozco Y, Espinoza-Juarez V, Serra-Barragán L and De Unanue A (2023) Current research trends on cognition, integrative complexity, and decision-making: A systematic literature review using activity theory and neuroscience. *Front. Psychol.* 14:1156696. doi: [10.3389/fpsyg.2023.1156696](https://doi.org/10.3389/fpsyg.2023.1156696)

*Mölsä ME, Lax M, Korhonen J, Gumpel TP and Söderberg P (2022) The experience sampling method in monitoring social interactions among children and adolescents in school: A systematic literature review. *Front. Psychol.* 13:844698. doi: [10.3389/fpsyg.2022.844698](https://doi.org/10.3389/fpsyg.2022.844698)

*Montiel C, Radziszewski S, Prilleltensky I and Houle J (2021) Fostering positive communities: A scoping review of community-level positive psychology interventions. *Front. Psychol.* 12:720793. doi: [10.3389/fpsyg.2021.720793](https://doi.org/10.3389/fpsyg.2021.720793)

*Moreno-Alcázar A, Treen D, Valiente-Gómez A, Sio-Eroles A, Pérez V, Amann BL and Radua J (2017) Efficacy of eye movement desensitization and reprocessing in children and adolescent with post-traumatic stress disorder: A meta-analysis of randomized controlled trials. *Front. Psychol.* 8:1750. doi: [10.3389/fpsyg.2017.01750](https://doi.org/10.3389/fpsyg.2017.01750)

*Moula Z, Palmer K and Walshe N (2022) A systematic review of arts-based interventions delivered to children and young people in nature or outdoor spaces: Impact on nature connectedness, health and wellbeing. *Front. Psychol.* 13:858781. doi: [10.3389/fpsyg.2022.858781](https://doi.org/10.3389/fpsyg.2022.858781)​

*Müller B, von Hagen A, Vannini N and Büttner G (2021) Measurement of the effects of school psychological services: A scoping review. *Front. Psychol.* 12:606228. doi: [10.3389/fpsyg.2021.606228](https://doi.org/10.3389/fpsyg.2021.606228)

*Mutaf-Yıldız B, Sasanguie D, De Smedt B and Reynvoet B (2020) Probing the relationship between home numeracy and children's mathematical skills: A systematic review. *Front. Psychol.* 11:2074. doi: [10.3389/fpsyg.2020.02074](https://doi.org/10.3389/fpsyg.2020.02074)

*Nadmilail AI, Mohd Matore MEE, Maat SM and Sheridan L (2023) Broad vs. narrow traits: a scoping review of measuring personality traits in teacher selection using the situational judgment test. *Front. Psychol.* 14:1217321. doi: [10.3389/fpsyg.2023.1217321](https://doi.org/10.3389/fpsyg.2023.1217321)

*Naor L and Mayseless O (2020) The wilderness solo experience: A unique practice of silence and solitude for personal growth. *Front. Psychol.* 11:547067. doi: [10.3389/fpsyg.2020.547067](https://doi.org/10.3389/fpsyg.2020.547067)

*Ni A, Cheung ACK and Shi J (2022) Effects of educational technology on reading achievement for Chinese K-12 English second language learners: A meta-analysis. *Front. Psychol.* 13:1025761. doi: [10.3389/fpsyg.2022.1025761](https://doi.org/10.3389/fpsyg.2022.1025761)

*Nogueira J, Gerardo B, Santana I, Simões MR and Freitas S (2022) The Assessment of Cognitive Reserve: A Systematic Review of the Most Used Quantitative Measurement Methods of Cognitive Reserve for Aging. *Front. Psychol*. 13:847186. [doi:10.3389/fpsyg.2022.847186](https://doi.org/10.3389/fpsyg.2022.847186)

*Norouzkhani N, Faramarzi M, Ghodousi Moghadam S, Karimi MA, Shokri Shirvani J, Bahari A, ShojaeiBaghini M, Eslami S and Tabesh H (2023) Identification of the informational and supportive needs of patients diagnosed with inflammatory bowel disease: a scoping review. *Front. Psychol.* 14:1055449. doi: [10.3389/fpsyg.2023.1055449](https://doi.org/10.3389/fpsyg.2023.1055449)​

*Nyongesa MK, Ssewanyana D, Mutua AM, Chongwo E, Scerif G, Newton CRJC and Abubakar A (2019) Assessing executive function in adolescence: A scoping review of existing measures and their psychometric robustness. *Front. Psychol.* 10:311. doi: [10.3389/fpsyg.2019.00311](https://doi.org/10.3389/fpsyg.2019.00311)​

*O’Grady AM and Nag S (2022) “Where’s Wally?” Identifying theory of mind in school-based social skills interventions. *Front. Psychol.* 13:600699. doi: [10.3389/fpsyg.2022.600699](https://doi.org/10.3389/fpsyg.2022.600699)​

*Oliveira S, Roberto MS, Pereira NS, Marques-Pinto A and Veiga-Simão AM (2021) Impacts of social and emotional learning interventions for teachers on teachers' outcomes: A systematic review with meta-analysis. *Front. Psychol.* 12:677217. doi: [10.3389/fpsyg.2021.677217](https://doi.org/10.3389/fpsyg.2021.677217)

*Orth Z, Moosajee F and Van Wyk B (2022) Measuring mental wellness of adolescents: A systematic review of instruments. *Front. Psychol.* 13:835601. doi: [10.3389/fpsyg.2022.835601](https://doi.org/10.3389/fpsyg.2022.835601)

*Özdemir O and Seçkin H (2024) Quantifying cognitive and affective impacts of Quizlet on learning outcomes: a systematic review and comprehensive meta-analysis. *Front. Psychol.* 15:1349835. doi: [10.3389/fpsyg.2024.1349835](https://doi.org/10.3389/fpsyg.2024.1349835)​

*Panadero E (2017) A Review of Self-regulated Learning: Six Models and Four Directions for Research. *Front. Psychol.* 8:422. doi: [10.3389/fpsyg.2017.00422](https://doi.org/10.3389/fpsyg.2017.00422)

*Para E, Dubreuil P, Miquelon P and Martin-Krumm C (2024) Interventions addressing the impostor phenomenon: a scoping review. *Front. Psychol.* 15:1360540. doi: [10.3389/fpsyg.2024.1360540](https://doi.org/10.3389/fpsyg.2024.1360540)​

*Parker DA and Roumell EA (2020) A Functional Contextualist Approach to Mastery Learning in Vocational Education and Training. *Front. Psychol.* 11:1479. [doi:10.3389/fpsyg.2020.01479](https://doi.org/10.3389/fpsyg.2020.01479)

*Paruzel A, Klug HJP and Maier GW (2021) The Relationship Between Perceived Corporate Social Responsibility and Employee-Related Outcomes: A Meta-Analysis. *Front. Psychol.* 12:607108. doi: [10.3389/fpsyg.2021.607108](https://doi.org/10.3389/fpsyg.2021.607108)

*Parveen K, Tran PQB, Alghamdi AA, Namaziandost E, Aslam S and Xiaowei T (2022) Identifying the Leadership Challenges of K-12 Public Schools During COVID-19 Disruption: A Systematic Literature Review*. Front. Psychol*. 13:875646. [doi:10.3389/fpsyg.2022.875646](https://doi.org/10.3389/fpsyg.2022.875646)

*Pasion R, Gonçalves AR, Fernandes C, Ferreira-Santos F, Barbosa F and Marques-Teixeira J (2017) Meta-Analytic Evidence for a Reversal Learning Effect on the Iowa Gambling Task in Older Adults. *Front. Psychol*. 8:1785. [doi:10.3389/fpsyg.2017.01785](https://doi.org/10.3389/fpsyg.2017.01785)

*Pedersen SKA, Andersen PN, Lugo RG, Andreassen M and Sütterlin S (2017) Effects of Music on Agitation in Dementia: A Meta-Analysis. *Front. Psychol.* 8:742. doi: [10.3389/fpsyg.2017.00742](https://doi.org/10.3389/fpsyg.2017.00742)​

*Peifer C, Wolters G, Harmat L, Heutte J, Tan J, Freire T, Tavares D, Fonte C, Andersen FO, van den Hout J, Šimleša M, Pola L, Ceja L and Triberti S (2022) A Scoping Review of Flow Research. *Front. Psychol.* 13:815665. doi: [10.3389/fpsyg.2022.815665](https://doi.org/10.3389/fpsyg.2022.815665)

*Peña-Sarrionandia A, Mikolajczak M and Gross JJ (2015) Integrating emotion regulation and emotional intelligence traditions: A meta-analysis. *Front. Psychol.* 6:160. doi: [10.3389/fpsyg.2015.00160](https://doi.org/10.3389/fpsyg.2015.00160)

*Peng C, Yue C, Avitt A and Chen Y (2021) A Systematic Review Approach to Find Robust Items of the Zimbardo Time Perspective Inventory. *Front. Psychol.* 12:627578. doi: [10.3389/fpsyg.2021.627578](https://doi.org/10.3389/fpsyg.2021.627578)

*Peng S, Fang Y, Othman AT and Liang J (2022) Meta-analysis and systematic review of physical activity on neurodevelopment disorders, depression, and obesity among children and adolescents. *Front. Psychol.* 13:940977. doi: [10.3389/fpsyg.2022.940977](https://doi.org/10.3389/fpsyg.2022.940977)​

*Peng W, Huang Q, Mao B, Lun D, Malova E, Simmons JV and Carcioppolo N (2023) When guilt works: a comprehensive meta-analysis of guilt appeals. *Front. Psychol.* 14:1201631. doi: [10.3389/fpsyg.2023.1201631](https://doi.org/10.3389/fpsyg.2023.1201631)

*Peng X (2023) Advancing Workplace Civility: a systematic review and meta-analysis of definitions, measurements, and associated factors. *Front. Psychol.* 14:1277188. doi: [10.3389/fpsyg.2023.1277188](https://doi.org/10.3389/fpsyg.2023.1277188)

*Pereira A, Lopes S, Magalhães P, Sampaio A, Chaleta E and Rosário P (2018) How Executive Functions Are Evaluated in Children and Adolescents with Cerebral Palsy? A Systematic Review. *Front. Psychol.* 9:21. doi: [10.3389/fpsyg.2018.00021](https://doi.org/10.3389/fpsyg.2018.00021)​

*Pereira T and Freire T (2021) Positive Youth Development in the Context of Climate Change: A Systematic Review. *Front. Psychol.* 12:786119. doi: [10.3389/fpsyg.2021.786119](https://doi.org/10.3389/fpsyg.2021.786119)​

*Pérez-Fernández A, Fernández-Berrocal P and Gutiérrez-Cobo MJ (2021) The Relationship Between Emotional Intelligence and Diabetes Management: A Systematic Review. *Front. Psychol.* 12:754362. doi: [10.3389/fpsyg.2021.754362](https://doi.org/10.3389/fpsyg.2021.754362)​

*Petersen B, Khalili-Mahani N, Murphy C, Sawchuk K, Phillips N, Li KZH and Hebblethwaite S (2023) The association between information and communication technologies, loneliness and social connectedness: A scoping review. *Front. Psychol.* 14:1063146. doi: [10.3389/fpsyg.2023.1063146](https://doi.org/10.3389/fpsyg.2023.1063146)​

*Petersen KJ, Qualter P and Humphrey N (2019) The Application of Latent Class Analysis for Investigating Population Child Mental Health: A Systematic Review. *Front. Psychol.* 10:1214. doi: [10.3389/fpsyg.2019.01214](https://doi.org/10.3389/fpsyg.2019.01214)

*Petre LM, Piepiora PA, Gemescu M and Gheorghe DA (2024) Internet- and mobile-based aftercare and relapse prevention interventions for anxiety and depressive disorders: a systematic review. *Front. Psychol*. 15:1474016. [doi:10.3389/fpsyg.2024.1474016](https://doi.org/10.3389/fpsyg.2024.1474016)

*Piccolo A, De Domenico C, Di Cara M, Settimo C, Corallo F, Leonardi S, Impallomeni C, Tripodi E, Quartarone A and Cucinotta F (2024) Parental involvement in robot-mediated intervention: a systematic review. *Front. Psychol.* 15:1355901. [doi:10.3389/fpsyg.2024.1355901](https://doi.org/10.3389/fpsyg.2024.1355901)

*Pietschnig J, Oberleiter S and Köhler MD (2024) Smoking behavior is associated with suicidality in individuals with psychosis and bipolar disorder: a systematic quantitative review and meta-analysis. *Front. Psychol*. 15:1369669. [doi:10.3389/fpsyg.2024.1369669](https://doi.org/10.3389/fpsyg.2024.1369669)

*Pilch I, Turska-Kawa A, Wardawy P, Olszanecka-Marmola A and Smołkowska-Jędo W (2023) Contemporary trends in psychological research on conspiracy beliefs. A systematic review. *Front. Psychol.* 14:1075779. doi: [10.3389/fpsyg.2023.1075779](https://doi.org/10.3389/fpsyg.2023.1075779)

*Pinto TM, Laurence PG, Macedo CR and Macedo EC (2021) Resilience Programs for Children and Adolescents: A Systematic Review and Meta-Analysis. *Front. Psychol*. 12:754115. [doi:10.3389/fpsyg.2021.754115](https://doi.org/10.3389/fpsyg.2021.754115)

*Pit-ten Cate IM and Glock S (2019) Teachers' Implicit Attitudes Toward Students From Different Social Groups: A Meta-Analysis. *Front. Psychol.* 10:2832. doi: [10.3389/fpsyg.2019.02832](https://doi.org/10.3389/fpsyg.2019.02832)

*Preciado M, Anguera MT, Olarte M and Lapresa D (2019) Observational Studies in Male Elite Football: A Systematic Mixed Study Review. *Front. Psychol.* 10:2077. [doi:10.3389/fpsyg.2019.02077](https://doi.org/10.3389/fpsyg.2019.02077)

*Prins J, van der Wilt F, van der Veen C and Hovinga D (2022) Nature play in early childhood education: A systematic review and meta ethnography of qualitative research. *Front. Psychol*. 13:995164. [doi:10.3389/fpsyg.2022.995164](https://doi.org/10.3389/fpsyg.2022.995164)

*Provenzi L, Giusti L, Caglia M, Rosa E, Mascheroni E and Montirosso R (2020) Evidence and Open Questions for the Use of Video-Feedback Interventions With Parents of Children With Neurodevelopmental Disabilities. *Front. Psychol.* 11:1374. doi: [10.3389/fpsyg.2020.01374](https://doi.org/10.3389/fpsyg.2020.01374)

*Queiruga-Dios M, Santos Sánchez MJ, Queiruga-Dios MÁ, Acosta Castellanos PM and Queiruga-Dios A (2021) Assessment Methods for Service-Learning Projects in Engineering in Higher Education: A Systematic Review. *Front. Psychol*. 12:629231. [doi:10.3389/fpsyg.2021.629231](https://doi.org/10.3389/fpsyg.2021.629231)

*Quintero J, Baldiris S, Rubira R, Cerón J and Velez G (2019) Augmented Reality in Educational Inclusion. A Systematic Review on the Last Decade. *Front. Psychol.* 10:1835. doi: [10.3389/fpsyg.2019.01835](https://doi.org/10.3389/fpsyg.2019.01835)​

*Rad D, Redeş A, Roman A, Ignat S, Lile R, Demeter E, Egerău A, Dughi T, Balaş E, Maier R, Kiss C, Torkos H and Rad G (2022) Pathways to inclusive and equitable quality early childhood education for achieving SDG4 goal—a scoping review. *Front. Psychol.* 13:955833. doi: [10.3389/fpsyg.2022.955833](https://doi.org/10.3389/fpsyg.2022.955833)

*Ribeiro-Silva E, Amorim C, Aparicio-Herguedas JL and Batista P (2022) Trends of Active Learning in Higher Education and Students’ Well-Being: A Literature Review. *Front. Psychol.* 13:844236. doi: [10.3389/fpsyg.2022.844236](https://doi.org/10.3389/fpsyg.2022.844236)

*Rossignoli-Palomeque T, Perez-Hernandez E and González-Marqués J (2018) Brain Training in Children and Adolescents: Is It Scientifically Valid? Front. Psychol. 9:565. [doi:10.3389/fpsyg.2018.00565](https://doi.org/10.3389/fpsyg.2018.00565)

*Rozental A, Bennett S, Forsström D, Ebert DD, Shafran R, Andersson G and Carlbring P (2018) Targeting Procrastination Using Psychological Treatments: A Systematic Review and Meta-Analysis. *Front. Psychol.* 9:1588. doi: [10.3389/fpsyg.2018.01588](https://doi.org/10.3389/fpsyg.2018.01588)​

*Rule A, Abbey C, Wang H, Rozelle S and Singh MK (2024) Measurement of flourishing: a scoping review. *Front. Psychol.* 15:1293943. doi: [10.3389/fpsyg.2024.1293943](https://doi.org/10.3389/fpsyg.2024.1293943)

*Sáiz-Manzanares MC, Alonso-Martínez L and Marticorena-Sánchez R (2022) A Systematic Review of the Use of T-Pattern and T-String Analysis (TPA) With Theme: An Analysis Using Mixed Methods and Data Mining Techniques. *Front. Psychol.* 13:943907. doi: [10.3389/fpsyg.2022.943907](https://doi.org/10.3389/fpsyg.2022.943907)

*Salehzadeh R and Ziaeian M (2024) Decision making in human resource management: a systematic review of the applications of analytic hierarchy process. *Front. Psychol.* 15:1400772. doi: [10.3389/fpsyg.2024.1400772](https://doi.org/10.3389/fpsyg.2024.1400772)

*Salgado JF and Moscoso S (2019) Meta-Analysis of Interrater Reliability of Supervisory Performance Ratings: Effects of Appraisal Purpose, Scale Type, and Range Restriction. *Front. Psychol.* 10:2282. doi: [10.3389/fpsyg.2019.02282](https://doi.org/10.3389/fpsyg.2019.02281)

*Salgado JF and Moscoso S (2019) Meta-Analysis of the Validity of General Mental Ability for Five Performance Criteria: Hunter and Hunter (1984) Revisited. *Front. Psychol.* 10:2227. doi: [10.3389/fpsyg.2019.02227](https://doi.org/10.3389/fpsyg.2019.02227)

*Salgado JF, Cuadrado D and Moscoso S (2022) Counterproductive Academic Behaviors and Academic Performance: A Meta-Analysis and a Path Analysis Model. *Front. Psychol.* 13:893775. doi: [10.3389/fpsyg.2022.893775](https://doi.org/10.3389/fpsyg.2022.893775)

*Sanabria-Mazo JP, Colomer-Carbonell A, Fernández-Vázquez Ó, Noboa-Rocamora G, Cardona-Ros G, McCracken LM, Montes-Pérez A, Castaño-Asins JR, Edo S, Borràs X, Sanz A, Feliu-Soler A and Luciano JV (2023) A systematic review of cognitive behavioral therapy-based interventions for comorbid chronic pain and clinically relevant psychological distress. *Front. Psychol*. 14:1200685. [doi:10.3389/fpsyg.2023.1200685](https://doi.org/10.3389/fpsyg.2023.1200685)

*Sánchez-Álvarez N, Berrios Martos MP and Extremera N (2020) A Meta-Analysis of the Relationship Between Emotional Intelligence and Academic Performance in Secondary Education: A Multi-Stream Comparison. *Front. Psychol.* 11:1517. doi: [10.3389/fpsyg.2020.01517](https://doi.org/10.3389/fpsyg.2020.01517)​

*Sánchez-López MT, Fernández-Berrocal P, Gómez-Leal R and Megías-Robles A (2022) Evidence on the Relationship Between Emotional Intelligence and Risk Behavior: A Systematic and Meta-Analytic Review. *Front. Psychol.* 13:810012. doi: [10.3389/fpsyg.2022.810012](https://doi.org/10.3389/fpsyg.2022.810012)

*Sankalaite S, Huizinga M, Dewandeleer J, Xu C, de Vries N, Hens E and Baeyens D (2021) Strengthening Executive Function and Self-Regulation Through Teacher-Student Interaction in Preschool and Primary School Children: A Systematic Review. *Front. Psychol*. 12:718262. [doi:10.3389/fpsyg.2021.718262](https://doi.org/10.3389/fpsyg.2021.718262)

*Schiera M, Faraci F, Mannino G and Vantaggiato L (2024) The impact of the pandemic on psychophysical well-being and quality of learning in the growth of adolescents (aged 11–13): a systematic review of the literature with a PRISMA method. *Front. Psychol*. 15:1384388. [doi:10.3389/fpsyg.2024.1384388](https://doi.org/10.3389/fpsyg.2024.1384388)

*Schnitzius M, Kirch A, Mess F and Spengler S (2019) Inside Out: A Scoping Review on the Physical Education Teacher’s Personality. *Front. Psychol.* 10:2510. doi: [10.3389/fpsyg.2019.02510](https://doi.org/10.3389/fpsyg.2019.02510)

*Schwab F, Hennighausen C, Adler DC and Carolus A (2018) Television Is Still “Easy” and Print Is Still “Tough”? More Than 30 Years of Research on the Amount of Invested Mental Effort. *Front. Psychol.* 9:1098. doi: [10.3389/fpsyg.2018.01098](https://doi.org/10.3389/fpsyg.2018.01098)

*Seghier ML, Fahim MA and Habak C (2019) Educational fMRI: From the Lab to the Classroom. *Front. Psychol*. 10:2769. [doi:10.3389/fpsyg.2019.02769](https://doi.org/10.3389/fpsyg.2019.02769)

*Shadiev R and Wang X (2022) A Review of Research on Technology-Supported Language Learning and 21st Century Skills. *Front. Psychol.* 13:897689. doi: [10.3389/fpsyg.2022.897689](https://doi.org/10.3389/fpsyg.2022.897689)

*Shaojie T, Samad AA and Ismail L (2022) Systematic literature review on audio-visual multimodal input in listening comprehension. *Front. Psychol.* 13:980133. [doi:10.3389/fpsyg.2022.980133](https://doi.org/10.3389/fpsyg.2022.980133)

*Sharif Nia H, Froelicher ES, Hosseini L and Ashghali Farahani M (2022) Evaluation of Psychometric Properties of Hardiness Scales: A Systematic Review. *Front. Psychol.* 13:840187. doi: [10.3389/fpsyg.2022.840187](https://doi.org/10.3389/fpsyg.2022.840187)​

*Shen Y, Sun F, Zhang A and Wang K (2021) The Effectiveness of Psychosocial Interventions for Elder Abuse in Community Settings: A Systematic Review and Meta-Analysis. *Front. Psychol.* 12:679541. doi: [10.3389/fpsyg.2021.679541](https://doi.org/10.3389/fpsyg.2021.679541)​

*Shen Z and Zhao S (2022) Legal Instructional Design by Deep Learning Theory Under the Background of Educational Psychology. *Front. Psychol.* 13:917174. doi: [10.3389/fpsyg.2022.917174](https://doi.org/10.3389/fpsyg.2022.917174)

*Shi J, Cheung ACK and Ni A (2022). The effectiveness of Promoting Alternative Thinking Strategies program: A meta-analysis. *Front. Psychol.* 13:1030572. [doi: 10.3389/fpsyg.2022.1030572](https://doi.org/10.3389/fpsyg.2022.1030572)

*Shi Y (2021) The Interactive Effect of EFL Teachers’ Emotions and Cognitions on Their Pedagogical Practices. *Front. Psychol.* 12:811721. doi: [10.3389/fpsyg.2021.811721](https://doi.org/10.3389/fpsyg.2021.811721)

*Shu K (2022). Teachers’ Commitment and Self-Efficacy as Predictors of Work Engagement and Well-Being. *Front. Psychol.* 13:850204. [doi: 10.3389/fpsyg.2022.850204](https://doi.org/10.3389/fpsyg.2022.850204)

*Siegel M, Assenmacher C, Meuwly N and Zemp M (2021). The Legal Vulnerability Model for Same-Sex Parent Families: A Mixed Methods Systematic Review and Theoretical Integration. *Front. Psychol.* 12:644258. [doi: 10.3389/fpsyg.2021.644258](https://doi.org/10.3389/fpsyg.2021.644258)​

*Sierra-Díaz MJ, González-Víllora S, Pastor-Vicedo JC and López-Sánchez GF (2019) Can We Motivate Students to Practice Physical Activities and Sports Through Models-Based Practice? A Systematic Review and Meta-Analysis of Psychosocial Factors Related to Physical Education. *Front. Psychol*. 10:2115. [doi:10.3389/fpsyg.2019.02115](https://doi.org/10.3389/fpsyg.2019.02115)

*Smale-Jacobse AE, Meijer A, Helms-Lorenz M and Maulana R (2019). Differentiated Instruction in Secondary Education: A Systematic Review of Research Evidence. *Front. Psychol.* 10:2366. [doi: 10.3389/fpsyg.2019.02366](https://doi.org/10.3389/fpsyg.2019.02366)

*Song C, Ge S, Xue J and Yao W (2022). Physical Exercise of Primary and Middle School Students From the Perspective of Educational Psychology and Parents’ Entrepreneurship Education. *Front. Psychol.* 12:777069. [doi: 10.3389/fpsyg.2021.777069](https://doi.org/10.3389/fpsyg.2021.777069)

*Soufi Amlashi R, Majzoobi M and Forstmeier S (2024) The Relationship Between Acculturative Stress and Psychological Outcomes in International Students: A Systematic Review and Meta-Analysis. *Front. Psychol.* 15:1403807. [doi:10.3389/fpsyg.2024.1403807](https://doi.org/10.3389/fpsyg.2024.1403807).

*Stangl FJ, Riedl R, Kiemeswenger R and Montag C (2023). Negative psychological and physiological effects of social networking site use: The example of Facebook. *Front. Psychol.* 14:1141663. [doi: 10.3389/fpsyg.2023.1141663](https://doi.org/10.3389/fpsyg.2023.1141663)

*Stapley E, Vainieri I, Li E, Merrick H, Jeffery M, Foreman S, Casey P, Ullman R and Cortina M (2021). A Scoping Review of the Factors That Influence Families’ Ability or Capacity to Provide Young People With Emotional Support Over the Transition to Adulthood. *Front. Psychol.* 12:732899. [doi: 10.3389/fpsyg.2021.732899](https://doi.org/10.3389/fpsyg.2021.732899)

*Stockman D, Haney L, Uzieblo K, Littleton H, Keygnaert I, Lemmens G and Verhofstadt L (2023) An ecological approach to understanding the impact of sexual violence: a systematic meta-review. *Front. Psychol.* 14:1032408. [doi:10.3389/fpsyg.2023.1032408](https://doi.org/10.3389/fpsyg.2023.1032408)

*Su X and Chan KL (2023). The associations of decent work with wellbeing and career capabilities: a meta-analysis. *Front. Psychol.* 14:1068599. [doi: 10.3389/fpsyg.2023.1068599](https://doi.org/10.3389/fpsyg.2023.1068599)

*Subara-Zukic E, Cole MH, McGuckian TB, Steenbergen B, Green D, Smits-Engelsman BC, Lust JM, Abdollahipour R, Domellöf E, Deconinck FJA, Blank R, and Wilson PH (2022) Behavioral and Neuroimaging Research on Developmental Coordination Disorder (DCD): A Combined Systematic Review and Meta-Analysis of Recent Findings. Front. Psychol. 13:809455. doi: [10.3389/fpsyg.2022.809455](https://doi.org/10.3389/fpsyg.2022.809455)​

*Sulla F, Monacis D and Limone P (2023) A systematic review of the role of teachers’ support in promoting socially shared regulatory strategies for learning. *Front. Psychol*. 14:1208012. [doi:10.3389/fpsyg.2023.1208012](https://doi.org/10.3389/fpsyg.2023.1208012)

*Sun X (2020) Exploration and Practice of “Internet + Maker Education” University Innovative Entrepreneurship Education Model From the Perspective of Positive Psychology. Front. Psychol. 11:891. doi: [10.3389/fpsyg.2020.00891](https://doi.org/10.3389/fpsyg.2020.00891)

*Szpunar KK, Moulton ST and Schacter DL (2013). Mind wandering and education: from the classroom to online learning. *Front. Psychol.* 4:495. [doi: 10.3389/fpsyg.2013.00495](https://doi.org/10.3389/fpsyg.2013.00495)

*Tan YT, McPherson GE, Peretz I, Berkovic SF and Wilson SJ (2014) The genetic basis of music ability. *Front. Psychol*. 5:658. doi: [10.3389/fpsyg.2014.00658](https://doi.org/10.3389/fpsyg.2014.00658)

*Tang YX and He WG (2023) Meta-analysis of the relationship between university students’ anxiety and academic performance during the coronavirus disease 2019 pandemic. Front. Psychol. 14:1018558. doi: [10.3389/fpsyg.2023.1018558](https://doi.org/10.3389/fpsyg.2023.1018558)​

*Tanious R, Gérain P, Jacquet W and Van Hoof E (2023) A scoping review of life skills development and transfer in emerging adults. *Front. Psychol.* 14:1275094. [doi:10.3389/fpsyg.2023.1275094](https://doi.org/10.3389/fpsyg.2023.1275094)

*Tebar-Yébana S, Navarro-Mateu D, Gómez-Domínguez MT and Gómez-Dominguez V (2024) Educational inclusion and satisfaction of families of students with intellectual disabilities: a bibliometric study*. Front. Psychol.* 15:1335168. [doi:10.3389/fpsyg.2024.1335168](https://doi.org/10.3389/fpsyg.2024.1335168)

*Thakur D, Martens MA, Smith DS and Roth E (2018) Williams Syndrome and Music: A Systematic Integrative Review. *Front. Psychol*. 9:2203. [doi:10.3389/fpsyg.2018.02203](https://doi.org/10.3389/fpsyg.2018.02203)

*Tian Z, Kuang K, Wilson SR, Buzzanell PM, Ye J, Mao X and Wei H (2024) Measuring resilience for Chinese-speaking populations: a systematic review of Chinese resilience scales. Front. Psychol. 15:1293857. doi: [10.3389/fpsyg.2024.1293857](https://doi.org/10.3389/fpsyg.2024.1293857)​

*Tinajero C, Mayo ME, Villar E and Martínez-López Z (2024) Classic and modern models of self-regulated learning: integrative and componential analysis. Front. Psychol. 15:1307574. doi: [10.3389/fpsyg.2024.1307574](https://doi.org/10.3389/fpsyg.2024.1307574)​

*Tlili A, Denden M, Duan A, Padilla-Zea N, Huang R, Sun T and Burgos D (2022) Game-Based Learning for Learners With Disabilities—What Is Next? A Systematic Literature Review From the Activity Theory Perspective. *Front. Psychol*. 12:814691. [doi:10.3389/fpsyg.2021.814691](https://doi.org/10.3389/fpsyg.2021.814691)

*Tokuhama-Espinosa T, Simmers K, Batchelor D, Nelson AD and Borja C (2023) A Theory of Mental Frameworks. Front. Psychol. 14:1220664. doi: [10.3389/fpsyg.2023.1220664](https://doi.org/10.3389/fpsyg.2023.1220664)

*Too EK, Chongwo E, Mabrouk A and Abubakar A (2022) Adolescent Connectedness: A Scoping Review of Available Measures and Their Psychometric Properties. Front. Psychol. 13:856621. doi: [10.3389/fpsyg.2022.856621](https://doi.org/10.3389/fpsyg.2022.856621)

*Torgersen G-E, Boe O, Magnussen LI, Olsen DS and Scordato L (2024) Innovation in the realm of the unforeseen: a review of competence needed. Front. Psychol. 15:1166878. doi: [10.3389/fpsyg.2024.1166878](https://doi.org/10.3389/fpsyg.2024.1166878)

*Torrijos-Muelas M, González-Víllora S and Bodoque-Osma AR (2021) The Persistence of Neuromyths in the Educational Settings: A Systematic Review. *Front. Psychol*. 11:591923. [doi:10.3389/fpsyg.2020.591923](https://doi.org/10.3389/fpsyg.2020.591923)

*Toyama M and Yamazaki Y (2021) Classroom Interventions and Foreign Language Anxiety: A Systematic Review With Narrative Approach. *Front. Psychol*. 12:614184. [doi:10.3389/fpsyg.2021.614184](https://doi.org/10.3389/fpsyg.2021.614184)

*Tronchoni H, Izquierdo C and Anguera MT (2022) A systematic review on lecturing in contemporary university teaching. Front. Psychol. 13:971617. doi: [10.3389/fpsyg.2022.971617](https://doi.org/10.3389/fpsyg.2022.971617)

*Trypke D, Zuk P, and Rosler M (2023). Neurodevelopmental disorders and social media use: A systematic review. *Front. Psychol.* 14:1423456. doi: [10.3389/fpsyg.2023.1267023](https://doi.org/10.3389/fpsyg.2023.1267023)

*Tullberg M (2022). Affordances of musical instruments: Conceptual consideration. *Front. Psychol.* 13:974820. [doi:10.3389/fpsyg.2022.974820](https://doi.org/10.3389/fpsyg.2022.974820)

*Ugalde L, Santiago-Garabieta M, Villarejo-Carballido B and Puigvert L (2021) Impact of Interactive Learning Environments on Learning and Cognitive Development of Children With Special Educational Needs: A Literature Review. *Front. Psychol.* 12:674033. [doi:10.3389/fpsyg.2021.674033](https://doi.org/10.3389/fpsyg.2021.674033)

*Ünal K, Myyry L and Toom A (2024) Turkish teachers’ values with rational and non-rational truth and teacher emotions in teaching. *Front. Psychol*. 15:1395920. [doi**:**10.3389/fpsyg.2024.1395920](https://doi.org/10.3389/fpsyg.2024.1395920)

*Vadillo MA, Kostopoulou O, and Shanks DR (2015). A critical review and meta-analysis of the unconscious thought effect in medical decision making. *Front. Psychol.* 6:636. [doi:10.3389/fpsyg.2015.00636](https://doi.org/10.3389/fpsyg.2015.00636)

*van der Meulen K, Granizo L and del Barrio C (2021) Emotional Peer Support Interventions for Students With SEND: A Systematic Review. *Front. Psychol*. 12:797913. [doi:10.3389/fpsyg.2021.797913](https://doi.org/10.3389/fpsyg.2021.797913)

*Varela-Moreno E, Carreira Soler M, Guzmán-Parra J, Jódar-Sánchez F, Mayoral-Cleries F and Anarte-Ortíz MT (2022) Effectiveness of eHealth-Based Psychological Interventions for Depression Treatment in Patients With Type 1 or Type 2 Diabetes Mellitus: A Systematic Review. *Front. Psychol*. 12:746217. [doi:10.3389/fpsyg.2021.746217](https://doi.org/10.3389/fpsyg.2021.746217)

*Veloso A, Vicente SG, and Filipe MG (2020). Effectiveness of cognitive training for school-aged children and adolescents with ADHD: A systematic review. *Front. Psychol.* 10:2983. doi: [10.3389/fpsyg.2019.02983](https://doi.org/10.3389/fpsyg.2019.02983)

*Velotti P, Beomonte Zobel S, Rogier G and Tambelli R (2018) Exploring Relationships: A Systematic Review on Intimate Partner Violence and Attachment. *Front. Psychol.* 9:1166. [doi:10.3389/fpsyg.2018.01166](https://doi.org/10.3389/fpsyg.2018.01166)

*Verschuren CM, Tims M, and de Lange AH (2021). Negative work behavior: Toward an integrated definition. *Front. Psychol.* 12:726973. [doi:10.3389/fpsyg.2021.726973](https://doi.org/10.3389/fpsyg.2021.726973)

*Vigdal JS and Brønnick KK (2022). Helicopter parenting and its relationship with anxiety and depression: A systematic review. *Front. Psychol.* 13:872981. [doi:10.3389/fpsyg.2022.872981](https://doi.org/10.3389/fpsyg.2022.872981)

*Vistorte AOR, Deroncele-Acosta A, Ayala JLM, Barrasa A, López-Granero C and Martí-González M (2024) Integrating artificial intelligence to assess emotions in learning environments: a systematic literature review. *Front. Psychol*. 15:1387089. [doi:10.3389/fpsyg.2024.1387089](https://doi.org/10.3389/fpsyg.2024.1387089)

*Walugembe A, Ntayi J, Olupot C and Elasu J (2022) Adaptive behaviors in education institutions before and after COVID-19: A systematic literature review. *Front. Psychol.* 13:1017321. [doi:10.3389/fpsyg.2022.1017321](https://doi.org/10.3389/fpsyg.2022.1017321)

*Wang F and Liu Y (2022). Resilience in the relationship between English learners' motivation and well-being. *Front. Psychol.* 13:915456. [doi:10.3389/fpsyg.2022.915456](https://doi.org/10.3389/fpsyg.2022.915456)

*Wang G, Wang Y and Gai X (2021) A meta-analysis of the effects of mental contrasting with implementation intentions on goal attainment. *Front. Psychol.* 12:565202. [doi: 10.3389/fpsyg.2021.565202](https://doi.org/10.3389/fpsyg.2021.565202)

*Wang H and Hall NC (2018). Teachers' causal attributions: A systematic review. *Front. Psychol.* 9:2305. [doi:10.3389/fpsyg.2018.02305](https://doi.org/10.3389/fpsyg.2018.02305)

*Wang L (2021) The role of students’ self-regulated learning, grit, and resilience in second language learning. *Front. Psychol.* 12:800488. [doi: 10.3389/fpsyg.2021.800488](https://doi.org/10.3389/fpsyg.2021.800488)

*Wang LH and Yu ZG (2023) Gender-moderated effects of academic self-concept on achievement, motivation, performance, and self-efficacy: A systematic review. *Front. Psychol.* 14:1136141. [doi:10.3389/fpsyg.2023.1136141](https://doi.org/10.3389/fpsyg.2023.1136141)

*Wang M, Wang H and Shi Y (2022) The role of English as a foreign language learners’ grit and foreign language anxiety in their willingness to communicate: Theoretical perspectives. *Front. Psychol.* 13:1002562. [doi: 10.3389/fpsyg.2022.1002562](https://doi.org/10.3389/fpsyg.2022.1002562)

*Wang R-J and Shih Y-H (2022) Improving the quality of teacher education for sustainable development of Taiwan's education system: A systematic review on the research issues of teacher education after the implementation of 12-year national basic education. *Front. Psychol.* 13:921839. [doi: 10.3389/fpsyg.2022.921839](https://doi.org/10.3389/fpsyg.2022.921839)

*Wang X and Wang WL (2024) The tenure track employment system in colleges and universities in China: A scoping review of the Chinese literature. *Front. Psychol.* 14:1271110. [doi: 10.3389/fpsyg.2023.1271110](https://doi.org/10.3389/fpsyg.2023.1271110)

*Wang X, Li D and Li S (2023) Childhood trauma and problematic internet use: A meta-analysis based on students in mainland China. *Front. Psychol.* 14:1115129. [doi: 10.3389/fpsyg.2023.1115129](https://doi.org/10.3389/fpsyg.2023.1115129)​

*Wang Y (2022) Mental health education on college students’ English vocabulary memorization from the perspective of STEAM education. *Front. Psychol.* 13:944465. [doi: 10.3389/fpsyg.2022.944465](https://doi.org/10.3389/fpsyg.2022.944465)​

*Wang Y and Wang Y (2022) The interrelationship between emotional intelligence, self-efficacy, and burnout among foreign language teachers: A meta-analytic review. *Front. Psychol.* 13:913638. [doi: 10.3389/fpsyg.2022.913638](https://doi.org/10.3389/fpsyg.2022.913638)

*Ware AT, Kirkovski M, and Lum JAG (2020) Meta-analysis reveals a bilingual advantage that is dependent on task and age. *Front. Psychol.* 11:1458. [doi: 10.3389/fpsyg.2020.01458](https://doi.org/10.3389/fpsyg.2020.01458)​

*Willis S, Neil R, Mellick MC, and Wasley D (2019) The relationship between occupational demands and well-being of performing artists: A systematic review. *Front. Psychol.* 10:393. [doi: 10.3389/fpsyg.2019.00393](https://doi.org/10.3389/fpsyg.2019.00393)

*Wisniewski B, Zierer K, and Hattie J (2020) The power of feedback revisited: A meta-analysis of educational feedback research. *Front. Psychol.* 10:3087. [doi: 10.3389/fpsyg.2019.03087](https://doi.org/10.3389/fpsyg.2019.03087)​

*Wu C-L, Lin T-J, Chiou G-L, Lee C-Y, Luan H, Tsai M-J, Potvin P, and Tsai C-C (2021) A systematic review of MRI neuroimaging for education research. *Front. Psychol.* 12:617599. [doi: 10.3389/fpsyg.2021.617599](https://doi.org/10.3389/fpsyg.2021.617599)

*Wu M and Sarker MNI (2022) Assessment of multiple subjects' synergetic governance in vocational education. *Front. Psychol.* 13:947665. [doi: 10.3389/fpsyg.2022.947665](https://doi.org/10.3389/fpsyg.2022.947665)

*WuC-L, Huang S-Y, Chen P-Z and Chen H-C (2020) A Systematic Review of Creativity-Related Studies Applying the Remote Associates Test From 2000 to 2019. *Front. Psychol*. 11:573432. [doi:10.3389/fpsyg.2020.573432](https://doi.org/10.3389/fpsyg.2020.573432)

*Xie C, Wang M, and Hu H (2018) Effects of constructivist and transmission instructional models on mathematics achievement in mainland China: A meta-analysis. *Front. Psychol.* 9:1923. [doi: 10.3389/fpsyg.2018.01923](https://doi.org/10.3389/fpsyg.2018.01923)​

*Xie F and Derakhshan A (2021) A conceptual review of positive teacher interpersonal communication behaviors in the instructional context. *Front. Psychol.* 12:708490. [doi: 10.3389/fpsyg.2021.708490](https://doi.org/10.3389/fpsyg.2021.708490)

*Xie H, Peng J, Qin M, Huang X, Tian F and Zhou Z (2018) Can Touchscreen Devices be Used to Facilitate Young Children’s Learning? A Meta-Analysis of Touchscreen Learning Effect. *Front. Psychol*. 9:2580. [doi:10.3389/fpsyg.2018.02580](https://doi.org/10.3389/fpsyg.2018.02580)

*Xu C and Zhang Z (2021) The effect of law students in entrepreneurial psychology under the artificial intelligence technology. *Front. Psychol.* 12:731713. [doi: 10.3389/fpsyg.2021.731713](https://doi.org/10.3389/fpsyg.2021.731713)​

*Xu L and Zhu X (2022) The predictive role of Chinese English as a foreign language teachers' psychological capital in their job commitment and academic optimism. *Front. Psychol.* 13:916433. [doi: 10.3389/fpsyg.2022.916433](https://doi.org/10.3389/fpsyg.2022.916433)​

*Xu M and Zeng S (2023) Optimal timing of treatment for errors in second language learning – A systematic review of corrective feedback timing. *Front. Psychol.* 14:1026174. [doi: 10.3389/fpsyg.2023.1026174](https://doi.org/10.3389/fpsyg.2023.1026174)

*Xu T and Wang H (2023) High prevalence of anxiety, depression, and stress among remote learning students during the COVID-19 pandemic: Evidence from a meta-analysis. *Front. Psychol.* 13:1103925. [doi: 10.3389/fpsyg.2022.1103925](https://doi.org/10.3389/fpsyg.2022.1103925)

*Xu T and Xue L (2023) Satisfaction with online education among students, faculty, and parents before and after the COVID-19 outbreak: Evidence from a meta-analysis. *Front. Psychol.* 14:1128034. [doi: 10.3389/fpsyg.2023.1128034](https://doi.org/10.3389/fpsyg.2023.1128034)​

*Xuan D, Zhu D, and Xu W (2021) The teaching pattern of law majors using artificial intelligence and deep neural network under educational psychology. *Front. Psychol.* 12:711520. [doi: 10.3389/fpsyg.2021.711520](https://doi.org/10.3389/fpsyg.2021.711520)​

*Xuan Q, Cheung A, and Sun D (2022) The effectiveness of formative assessment for enhancing reading achievement in K-12 classrooms: A meta-analysis. *Front. Psychol.* 13:990196. [doi: 10.3389/fpsyg.2022.990196](https://doi.org/10.3389/fpsyg.2022.990196)​

*Yang D, Chen P, Wang H, Wang K, and Huang R (2022) Teachers’ autonomy support and student engagement: A systematic literature review of longitudinal studies. *Front. Psychol.* 13:925955. [doi: 10.3389/fpsyg.2022.925955](https://doi.org/10.3389/fpsyg.2022.925955)

*Yang H-m (2022) English as a foreign language teachers’ well-being, their apprehension, and stress: The mediating role of hope and optimism. *Front. Psychol.* 13:855282. [doi: 10.3389/fpsyg.2022.855282](https://doi.org/10.3389/fpsyg.2022.855282)​

*Yang W, Liu H, Chen N, Xu P, and Lin X (2020) Is early spatial skills training effective? A meta-analysis. *Front. Psychol.* 11:1938. [doi: 10.3389/fpsyg.2020.01938](https://doi.org/10.3389/fpsyg.2020.01938)

*Yang X, Xu XY, Guo L, Zhang Y, Wang SS, and Li Y (2022) Effect of leisure activities on cognitive aging in older adults: A systematic review and meta-analysis. *Front. Psychol.* 13:1080740. [doi: 10.3389/fpsyg.2022.1080740](https://doi.org/10.3389/fpsyg.2022.1080740)

*Yao D and Wallace MP (2021) Language assessment for immigration: A review of validation research over the last two decades. *Front. Psychol.* 12:773132. [doi: 10.3389/fpsyg.2021.773132](https://doi.org/10.3389/fpsyg.2021.773132)​

*Ye W, Teig N and Blömeke S (2024) Systematic review of protective factors related to academic resilience in children and adolescents: unpacking the interplay of operationalization, data, and research method. *Front. Psychol*. 15:1405786. [doi:10.3389/fpsyg.2024.1405786](https://doi.org/10.3389/fpsyg.2024.1405786)

*Yildiz B, Yildiz T, Ozbilgin M, and Yildiz H (2022) Counterintuitive consequences of COVID-19 on healthcare workers: A meta-analysis of the relationship between work engagement and job satisfaction. *Front. Psychol.* 13:962830. [doi: 10.3389/fpsyg.2022.962830](https://doi.org/10.3389/fpsyg.2022.962830)​

*Yilmazer E, Hamamci Z, and Türk F (2024) Effects of mindfulness on test anxiety: A meta-analysis. *Front. Psychol.* 15:1401467. [doi: 10.3389/fpsyg.2024.1401467](https://doi.org/10.3389/fpsyg.2024.1401467)​

*Yokoyama S (2019) Academic self-efficacy and academic performance in online learning: A mini review. *Front. Psychol.* 9:2794. [doi: 10.3389/fpsyg.2018.02794](https://doi.org/10.3389/fpsyg.2018.02794)​

*Yoon S, Yang Y, Ro E, Ahn W-Y, Kim J, Shin S-H, Chey J, and Choi K-H (2021) Reliability, and convergent and discriminant validity of gaming disorder scales: A meta-analysis. *Front. Psychol.* 12:764209. [doi: 10.3389/fpsyg.2021.764209](https://doi.org/10.3389/fpsyg.2021.764209)​

*Young CJ, Levine SC, and Mix KS (2018) The connection between spatial and mathematical ability across development. *Front. Psychol.* 9:755. [doi: 10.3389/fpsyg.2018.00755](https://doi.org/10.3389/fpsyg.2018.00755)​

*Yu H (2023) The neuroscience basis and educational interventions of mathematical cognitive impairment and anxiety: a systematic literature review. *Front. Psychol*. 14:1282957. [doi:10.3389/fpsyg.2023.1282957](https://doi.org/10.3389/fpsyg.2023.1282957)

*Yu M, Wang H, and Xia G (2022) The review on the role of ambiguity of tolerance and resilience on students' engagement. *Front. Psychol.* 12:828894. [doi: 10.3389/fpsyg.2021.828894](https://doi.org/10.3389/fpsyg.2021.828894)​

*Yu R and Yang L (2021) ESL/EFL learners’ responses to teacher written feedback: Reviewing a recent decade of empirical studies. *Front. Psychol.* 12:735101. [doi: 10.3389/fpsyg.2021.735101](https://doi.org/10.3389/fpsyg.2021.735101)​

*Yu S, Tian L, Wang G, and Nie S (2024) Which ERP components are effective in measuring cognitive load in multimedia learning? A meta-analysis based on relevant studies. *Front. Psychol.* 15:1401005. [doi: 10.3389/fpsyg.2024.1401005](https://doi.org/10.3389/fpsyg.2024.1401005)​

*Yu Z, Xu W, and Sukjairungwattana P (2022) Meta-analyses of differences in blended and traditional learning outcomes and students' attitudes. *Front. Psychol.* 13:926947. [doi: 10.3389/fpsyg.2022.926947](https://doi.org/10.3389/fpsyg.2022.926947)

*Zeng Y, Wang Y, and Li S (2022) The relationship between teachers’ information technology integration self-efficacy and TPACK: A meta-analysis. *Front. Psychol.* 13:1091017. [doi: 10.3389/fpsyg.2022.1091017](https://doi.org/10.3389/fpsyg.2022.1091017)​

*Zhan Z, He L, and Zhong X (2024) How does problem-solving pedagogy affect creativity? A meta-analysis of empirical studies. *Front. Psychol.* 15:1287082. [doi: 10.3389/fpsyg.2024.1287082](https://doi.org/10.3389/fpsyg.2024.1287082)​

*Zhang J (2022) The impact of positive mood and future outlook on English as a foreign language students’ academic self-concept. *Front. Psychol.* 13:846422. [doi: 10.3389/fpsyg.2022.846422](https://doi.org/10.3389/fpsyg.2022.846422)

*Zhang L and Ma Y (2023) A study of the impact of project-based learning on student learning effects: A meta-analysis study. *Front. Psychol.* 14:1202728. [doi: 10.3389/fpsyg.2023.1202728](https://doi.org/10.3389/fpsyg.2023.1202728)​

*Zhang L, Xu X, Li Z, Chen L and Feng L (2022) Interpersonal Neural Synchronization Predicting Learning Outcomes From Teaching-Learning Interaction: A Meta-Analysis. *Front. Psychol*. 13:835147. [doi:10.3389/fpsyg.2022.835147](https://doi.org/10.3389/fpsyg.2022.835147)

*Zhang X (2021) The effect of English as a foreign language teachers’ optimism and affectivity on their psychological well-being. *Front. Psychol.* 12:816204. [doi: 10.3389/fpsyg.2021.816204](https://doi.org/10.3389/fpsyg.2021.816204)​

*Zhang Y, Li G, Liu C, Chen H, Guo J and Shi Z (2023) Mixed comparison of interventions for different exercise types on students with Internet addiction: a network meta-analysis. *Front. Psychol.* 14:1111195. [doi:10.3389/fpsyg.2023.1111195](https://doi.org/10.3389/fpsyg.2023.1111195)

*Zhao L and Yu J (2021) A meta-analytic review of moral disengagement and cyberbullying. *Front. Psychol.* 12:681299. [doi: 10.3389/fpsyg.2021.681299](https://doi.org/10.3389/fpsyg.2021.681299)​

*Zhao L, Xu P, Chen Y, and Yan S (2022) A literature review of the research on students’ evaluation of teaching in higher education. *Front. Psychol.* 13:1004487. [doi: 10.3389/fpsyg.2022.1004487](https://doi.org/10.3389/fpsyg.2022.1004487)​

*Zhao X and Wang D (2023) Grit in second language acquisition: A systematic review from 2017 to 2022. *Front. Psychol.* 14:1238788. [doi: 10.3389/fpsyg.2023.1238788](https://doi.org/10.3389/fpsyg.2023.1238788)

*Zheng H, Miao X, Dong Y, and Yuan D-C (2023) The relationship between grammatical knowledge and reading comprehension: A meta-analysis. *Front. Psychol.* 14:1098568. [doi: 10.3389/fpsyg.2023.1098568](https://doi.org/10.3389/fpsyg.2023.1098568)​

*Zhong H, Zhao C, Zhang F, and Zhang R (2022) Application of Educational Psychology-Based Dance Therapy in College Students’ Life Education. *Front. Psychol.* 13:784568. [doi: 10.3389/fpsyg.2022.784568](https://doi.org/10.3389/fpsyg.2022.784568)​

*Zhong Z, Feng Y, and Xu Y (2024) The impact of boarding school on student development in primary and secondary schools: A meta-analysis. *Front. Psychol.* 15:1359626. [doi: 10.3389/fpsyg.2024.1359626](https://doi.org/10.3389/fpsyg.2024.1359626)​

*Zhou K, Lu L, Hu L, and Wang Y (2022) Associations between two conceptualizations of materialism and subjective wellbeing in China: A meta-analysis of studies from 1998 to 2022. *Front. Psychol.* 13:982172. [doi: 10.3389/fpsyg.2022.982172](https://doi.org/10.3389/fpsyg.2022.982172)​

*Zhou Q (2021) The impact of cross-cultural adaptation on entrepreneurial psychological factors and innovation ability for new entrepreneurs. *Front. Psychol.* 12:724544. [doi: 10.3389/fpsyg.2021.724544](https://doi.org/10.3389/fpsyg.2021.724544)​

*Zhu X and Aryadoust V (2022) A synthetic review of cognitive load in distance interpreting: Toward an explanatory model. *Front. Psychol.* 13:899718. [doi: 10.3389/fpsyg.2022.899718](https://doi.org/10.3389/fpsyg.2022.899718)​

*Zhu Y, Deng L, and Wan K (2022) The association between parent-child relationship and problematic internet use among English- and Chinese-language studies: A meta-analysis. *Front. Psychol.* 13:885819. [doi: 10.3389/fpsyg.2022.885819](https://doi.org/10.3389/fpsyg.2022.885819)​

References of Special Issue

*Bonilla-Sánchez MdR (2024) Clinical experiences of intervention of neurodevelopmental disorders and difficulties in school learning from historical-cultural neuropsychology. *Front. Educ.* 9:1291732. [doi:10.3389/feduc.2024.1291732](https://doi.org/10.3389/feduc.2024.1291732)

*Cochon Drouet O, Lentillon-Kaestner V and Margas N (2023) Effects of the Jigsaw method on student educational outcomes: systematic review and meta-analyses. *Front. Psychol.* 14:1216437. doi: [10.3389/fpsyg.2023.1216437](https://doi.org/10.3389/fpsyg.2023.1216437)

*De la Fuente J and Martínez-Vicente JM (2024) Conceptual Utility Model for the Management of Stress and Psychological Wellbeing, CMMSPW™ in a university environment: theoretical basis, structure and functionality. *Front. Psychol.* 14:1299224. doi: [10.3389/fpsyg.2023.1299224](https://doi.org/10.3389/fpsyg.2023.1299224)​

*Fan H, Feng Y and Zhang Y (2024) Parental involvement and student creativity: a three-level meta-analysis. *Front. Psychol.* 15:1407279. doi: [10.3389/fpsyg.2024.1407279](https://doi.org/10.3389/fpsyg.2024.1407279)

*Hurtado E, Rosado E, Aoiz M, Quero S and Luis EO (2024) Factors associated with the permanence of doctoral students. A scoping review. *Front. Psychol.* 15:1390784. doi: [10.3389/fpsyg.2024.1390784](https://doi.org/10.3389/fpsyg.2024.1390784)​

*Kuznetsova E, Liashenko A, Zhozhikashvili N and Arsalidou M (2024) Giftedness identification and cognitive, physiological and psychological characteristics of gifted children: a systematic review. *Front. Psychol*. 15:1411981. [doi: 10.3389/fpsyg.2024.1411981](https://doi.org/10.3389/fpsyg.2024.1411981)

*López Martínez O, Lorca Garrido AJ and de Vicente-Yagüe Jara MI (2024) Indicators of verbal creative thinking: results of a Delphi panel. *Front. Psychol.* 15:1397861. doi: [10.3389/fpsyg.2024.1397861](https://doi.org/10.3389/fpsyg.2024.1397861)

*Romero-González M, Lavigne-Cerván R, Gamboa-Ternero S, Rodríguez-Infante G, Juárez-Ruiz de Mier R and Romero-Pérez JF (2023) Active Home Literacy Environment: parents’ and teachers’ expectations of its influence on affective relationships at home, reading performance, and reading motivation in children aged 6 to 8 years. *Front. Psychol.* 14:1261662. doi: [10.3389/fpsyg.2023.1261662](https://doi.org/10.3389/fpsyg.2023.1261662)

*Shao J, Chen Y, Wei X, Li X and Li Y (2023) Effects of regulated learning scaffolding on regulation strategies and academic performance: A meta-analysis. *Front. Psychol.* 14:1110086. doi: [10.3389/fpsyg.2023.1110086](https://doi.org/10.3389/fpsyg.2023.1110086)

*Stoltz T, Weger U and da Veiga M (2024). Consciousness and education: contributions by Piaget, Vygotsky and Steiner. *Front. Psychol.* 15:1411415. [doi: 10.3389/fpsyg.2024.1411415](https://doi.org/10.3389/fpsyg.2024.1411415)

*Ünal K, Myyry L and Toom A (2024) Turkish teachers’ values with rational and non-rational truth and teacher emotions in teaching. *Front. Psychol*. 15:1395920. [doi:10.3389/fpsyg.2024.1395920](https://doi.org/10.3389/fpsyg.2024.1395920)

*Wang Y and Li W (2023). The impostor phenomenon among doctoral students: A scoping review. *Front. Psychol.* 14:1233434. [doi:10.3389/fpsyg.2023.1233434](https://doi.org/10.3389/fpsyg.2023.1233434)

*Wang D and Li Y (2024). Career construction theory: Tools, interventions, and future trends. *Front. Psychol.* 15:1381233. [doi:10.3389/fpsyg.2024.1381233](https://doi.org/10.3389/fpsyg.2024.1381233)

*Wang S, Okada T and Takagi K (2023) How to effectively overcome fixation: A systematic review of fixation and defixation studies on the basis of fixation source and problem type. *Front. Educ.* 8:1183025. [doi: 10.3389/feduc.2023.1183025](https://doi.org/10.3389/feduc.2023.1183025)

*Wang T, Xu H, Li C, Zhang F and Wang J (2024) Dynamic insights into research trends and trajectories in early reading: An analytical exploration via dynamic topic modeling. *Front. Psychol.* 15:1326494. [doi: 10.3389/fpsyg.2024.1326494](https://doi.org/10.3389/fpsyg.2024.1326494)

*Wu J, Kuan G, Lou H, Hu X, Masri MN, Sabo A, and Kueh YC (2023) The impact of COVID-19 on students’ anxiety and its clarification: A systematic review. *Front. Psychol.* 14:1134703. [doi: 10.3389/fpsyg.2023.1134703](https://doi.org/10.3389/fpsyg.2023.1134703)

*Yu Q (2024) Foreign language anxiety research in System between 2004 and 2023: Looking back and looking forward. *Front. Psychol.* 15:1373290. [doi: 10.3389/fpsyg.2024.1373290](https://doi.org/10.3389/fpsyg.2024.1373290)​

*Zheng J, Lajoie S, and Li S (2023) Emotions in self-regulated learning: A critical literature review and meta-analysis. *Front. Psychol.* 14:1137010. [doi: 10.3389/fpsyg.2023.1137010](https://doi.org/10.3389/fpsyg.2023.1137010)​
